# Curated and harmonised transcriptomics datasets of interstitial lung diseases

**DOI:** 10.1016/j.dib.2025.112139

**Published:** 2025-10-14

**Authors:** Simo Inkala, Antonio Federico, Angela Serra, Dario Greco

**Affiliations:** a Finnish Hub for Development and Validation of Integrated Approaches (FHAIVE), Faculty of Medicine and Health Technology, Tampere University, 33100, Tampere, Finland; b Tampere Institute for Advanced Study, Tampere University, 33100, Tampere, Finland; c Division of Pharmaceutical Biosciences, Faculty of Pharmacy, University of Helsinki, 00100, Helsinki, Finland; d Institute of Biotechnology, University of Helsinki, 00100, Helsinki, Finland

**Keywords:** Data fairification, Harmonisation, Interstitial lung disease, Lung fibrosis, Network analysis, Omics data

## Abstract

This study provides manually curated and homogenised transcriptomics data of interstitial lung disease (ILD) patients retrieved from the NCBI Gene Expression Omnibus and European Nucleotide Archive repositories. The compendium includes 30 transcriptomics datasets generated with DNA microarrays and RNA sequencing (RNA-seq) technologies for a total of 1371 samples. All the datasets underwent metadata curation and harmonisation, data quality check, and preprocessing with standardised procedures. Furthermore, a robust data model was developed to standardise phenotypic data, thereby enhancing comparability across heterogeneous datasets. Gene expression data and lists of differentially expressed genes computed between ILD and healthy samples are provided. Among the ILDs included in this study, idiopathic pulmonary fibrosis (IPF) is the most represented worldwide. Co-expression networks of IPF and healthy samples were inferred, which are also included in this study. This study enhances the Findability, Accessibility, Interoperability, and Reusability (FAIR) of publicly available transcriptomic datasets related to ILDs. The resulting resource provides a integrated platform for the implementation and validation of systems biology and pharmacology approaches, facilitating the development of novel diagnostic and therapeutic strategies for ILDs.

Specifications Table

**Table:** 

Subject	Health Sciences, Medical Sciences & Pharmacology
Specific subject area	Transcriptomics, systems biology.
Type of data	Raw and processed gene expression estimates. Harmonised metadata. Differential gene expression analysis results. Gene co-expression networks.
Data collection	DNA microarray data was collected from Gene Expression Omnibus, while RNA-Seq data was collected from the European Nucleotide Archive. Several datasets were excluded from the collection. For more details see Supplementary File 1.
Data source location	Faculty of Medicine and Health Technology, Tampere University, Finland. (Latitude: 61.50683665811257, Longitude: 23.82389156932559)
Data accessibility	Repository name: Zenodo Data identification number: https://doi.org/10.5281/zenodo.10692129 Direct URL to data: https://zenodo.org/records/10692129
Related research article	None

## 1 Value of the Data

• The dataset enables the identification and characterization of ILD subtypes or endotypes. While ILDs often show similar clinical manifestations, they arise from heterogeneous molecular mechanisms; this molecular-level resolution of the data facilitates stratification of patients into biologically meaningful subgroups.
• Facilitating molecular patient stratification, this dataset fosters diagnostic innovation beyond traditional phenotype-based assessments, posing the roots for precision medicine approaches in ILD.
• The dataset serves as a resource for biomarker discovery. Although some biomarkers for IPF and ILDs are already known, identifying novel molecular vulnerabilities can enhance diagnostic capabilities and expand the pool of therapeutic targets.
• The data provide a robust foundation for systems pharmacology applications, which model diseases as complex molecular interaction networks. Using the included gene co-expression networks of IPF patients, researchers can model disease complexity, uncover regulatory pathways, and prioritize drug candidates with potentially improved efficacy and safety.
• The integrative and harmonized nature of the dataset, together with the included molecular interactomes, makes it particularly valuable for drug discovery applications, enabling compound in silico screening, target prioritization and validation of therapeutic hypotheses specific to different ILD phenotypes.

## 2 Background

ILDs encompass a spectrum of disorders characterised by chronic inflammation and scarring of the lung tissue, leading to a progressive impairment of the respiratory function. The most represented ILD in the global population is IPF. IPF is a chronic and progressive lung disease that has unknown origin. IPF is irreversible and usually lethal. To date, only a few treatment options with limited efficacy are available [1].

Transcriptomics technologies are established tools for elucidating the molecular intricacies underlying complex diseases, including ILDs. In addition, the analysis of molecular networks represents a well-established approach that leverages systems biology to investigate transcriptional relationships, define functionally related gene communities and pinpointing pivotal regulatory elements governing disease phenotypes [2]. Despite the wealth of transcriptomics data available for ILDs, these datasets are scattered across repositories, hindering comprehensive analysis and interpretation.

Here, we provide a comprehensive, harmonised, and “ready-to-use” FAIR collection of transcriptomics data of IPF patients, along with harmonised metadata. The data allow to elucidate the molecular mechanisms underlying ILDs through a wide range of possible applications. The scientific community will benefit from using these data by applying integrative methodologies (i.e. gene expression meta-analysis) across studies, technologies, and platforms to extrapolate robust gene expression signatures that underlie the clinical manifestations of the disease and its heterogeneity across patients. In a previous effort [3], we presented a curated collection of transcriptomics datasets of patients affected by inflammatory dermatological diseases, such as psoriasis and atopic dermatitis. Our work posed the roots for a thorough exploitation of the data that resulted in high-impact scientific investigations of these two skin diseases [4,5].

**Fig. 1:**
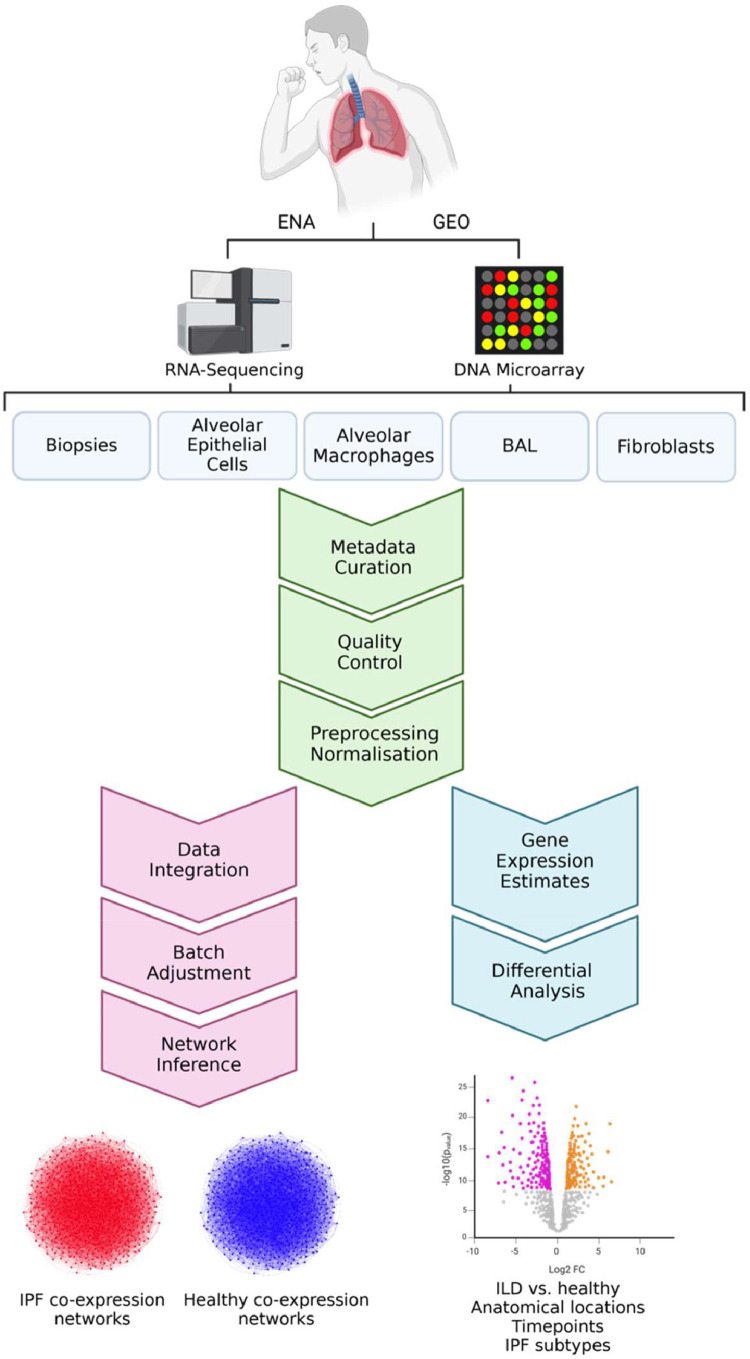
Analytical pipeline of the study. Created in BioRender: https://BioRender.com/cyj2oij.

In the same way, by consolidating and standardising ILD datasets, this work aims to catalyse future research endeavours, foster collaboration, and expedite the discovery of novel insights and therapeutic strategies. The analytical pipeline of the study is shown in Fig. 1.

## 3 Data Description

The preprocessed microarray datasets provided in this study were collected from the NCBI Gene Expression Omnibus (GEO) repository (https://www.ncbi.nlm.nih.gov/geo/), while the RNA-seq datasets were retrieved from the European Nucleotide Archive (ENA) (https://www.ebi.ac.uk/ena/browser/). Overall, 14 microarray datasets were collected, for a total of 471 samples, 297 of which were from patients affected by ILD and 174 samples from healthy individuals. These datasets were generated with commercially available Affymetrix and Agilent platforms (Table 1). Sixteen RNA-seq datasets were also retrieved, for a total of 900 samples, 589 of which were from patients affected by ILD and 311 samples from healthy individuals (Table 2). RNA-seq data were produced through either Illumina or Ion Torrent platforms.

**Table 1:** Microarray datasets included in the study.

GEO dataset ID	Platform	Citation	Number of disease samples included	Number of healthy samples included	Cell type
GSE53845	GPL6480	[6]	IPF: 40	8	Biopsy
GSE110147	GPL6244	[7]	IPF: 22 NSIP: 10 Mixed IPF-NSIP: 5	11	Biopsy
GSE21369	GPL570	[8]	UIP/IPF: 11 COP:2 HP: 2 NSIP:5 RB-ILD: 2 UF:1	6	Biopsy
GSE24206	GPL570	[9]	IPF: 17	6	Biopsy
GSE72073	GPL17586	[10]	IPF: 5	3	Biopsy
GSE10667	GPL4133	[11]	UIP: 31	15	Biopsy
GSE11196	GPL6732	[12]	IPF:12	12	Fibroblast
GSE129164	GPL17586	[13]	IPF: 10	10	Fibroblast
GSE44723	GPL570	[14]	IPF:10	4	Fibroblast
GSE144338	GPL22321	[15]	IPF:4 Lung Adenocarcinoma:4	4	Fibroblast
GSE40839	GPL96	[16]	UIP: 3 *Sc*-ILD: 8	10	Fibroblast
GSE70866	GPL14550 GPL17077	[17]	IPF: 62	20	BAL
GSE49072	GPL96	[18]	IPF: 15 Familial IPF: 8	61	Macrophage
GSE90010	GPL17692	[19]	IPF: 4 RB-ILD: 4	MDM: 4	Macrophage

Abbreviations; IPF: Idiopathic Pulmonary Fibrosis, NSIP: Nonspecific Interstitial Pneumonia, Mixe*d* IPF-NSIP: Mixed Idiopathic Pulmonary Fibrosis Nonspecific Intersitital Pneumonia, COP: Cryptogenic Organizing Pneumonia, HP: Hypersensitivity Pneumotitis, RB-ILD: Respiratory Bronchiolitis-Interstitial Lung Disease, UF: Uncharacterized Fibrosis, UIP: Usual Interstitial Pneumonia, *Sc*-ILD: Scleroderma- Associated Interstitial Lung Disease, MDM: Monocyte Derived Macrophage.

**Table 2:** RNA-seq dataset included in the study.

GEO dataset ID	Platform	Citation	Number of disease samples included	Number of healthy samples included	Cell type
GSE150910	GPL24676	[20]	IPF: 103 HP: 82	103	Biopsy
GSE213001	GPL21290	NA	IPF: 62 CLAD: 10 ILD:26	41	Biopsy
GSE124685	GPL17303	[21]	IPF: 49	35	Biopsy
GSE199949	GPL20301	NA	IPF: 26	16	Biopsy
GSE92592	GPL11154	[22]	IPF: 20	19	Biopsy
GSE199152	GPL16791	NA	IPF: 20 RA-UIP: 3	4	Biopsy
GSE166036	GPL20301	[23]	IPF: 10 *Sc*-ILD: 3	4	Biopsy
GSE184316	GPL17303	NA	IPF: 40 HP:36	24	Biopsy
GSE169500	GPL17301	[24]	IPF: 20	10	Biopsy
GSE99621	GPL16791	[25]	IPF: 18	8	Biopsy
GSE138283	GPL21697	[26]	IPF: 12	5	Biopsy
GSE185492	GPL24676	NA	IPF: 12	12	Fibroblast
GSE97038	GPL11154	[27]	IPF: 8	8	Fibroblast
GSE180415	GPL20301	[28]	IPF: 5	4	Fibroblast
GSE166036	GPL20301	[23]	IPF: 8 *Sc*-ILD: 2	4	BAL
GSE151673	GPL18573	[29]	IPF: 5	5	Epithelial
GSE205525	GPL24676	[30]	IPF: 9	9	Epithelial

Abbreviations; IPF: Idiopathic Pulmonary Fibrosis, HP: Hypersensitivity Pneumotitis, CLAD: Chronic Lung Allograft Dysfunction, ILD: Interstitial Lung Disease, RA-UIP: Rheumatoid Arthritis Associated Usual Interstitial Pneumonia, *Sc*-ILD: Scleroderma-Associated Interstitial Lung Disease.

**Fig. 2:**
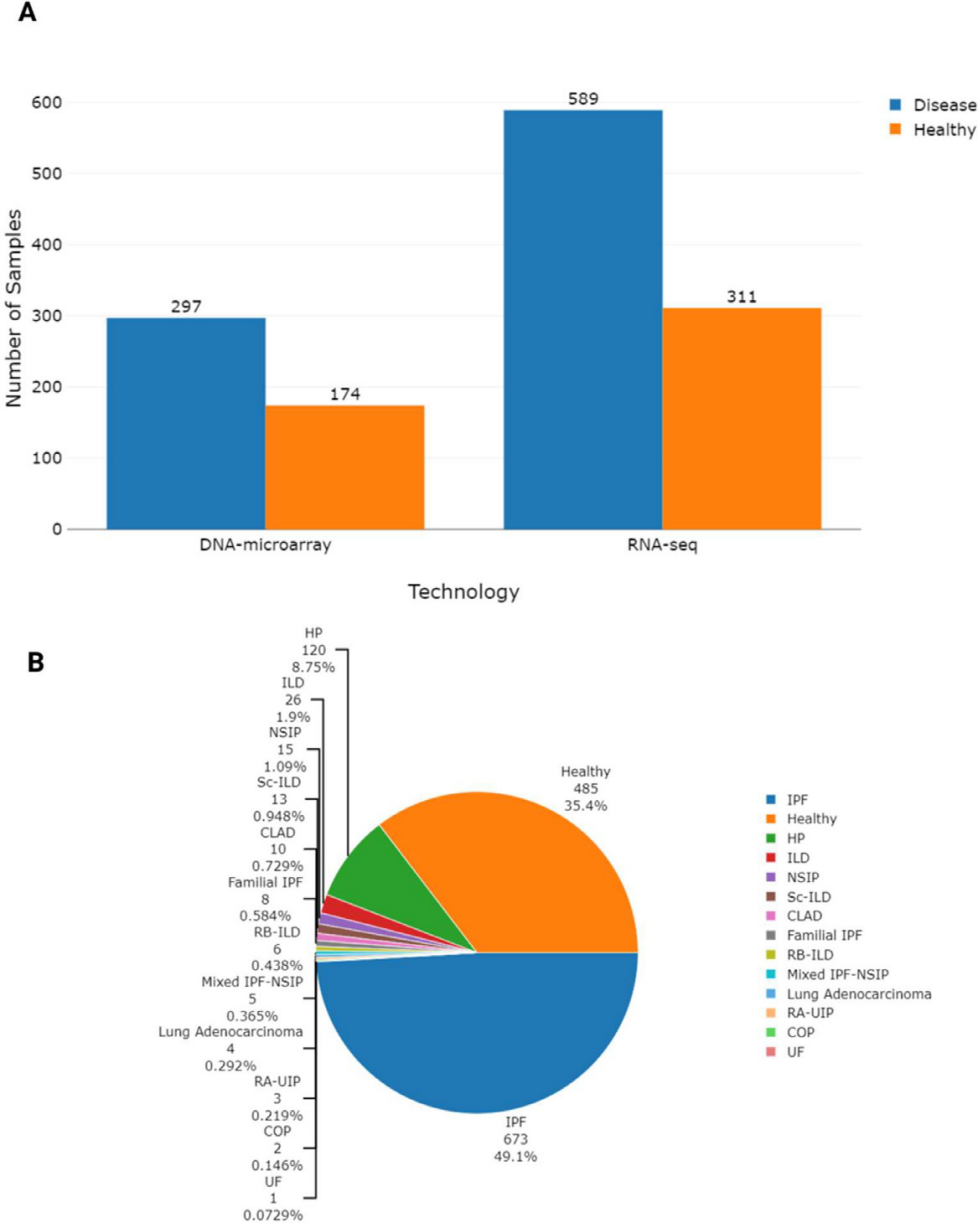
Distribution of samples across technologies and disease types. Panel A: Bar plot showing the number of samples stratified by platform type (DNA microarray and RNA-seq). Each bar is further divided to represent the number of samples derived from ILD patients and healthy individuals. Panel B: Pie chart showing the relative distribution of disease types across all ILD patient samples in the compendium. IPF is the most represented condition, accounting for 49.1 % of all ILD samples. This visualisation highlights both the technological diversity and disease heterogeneity present in the dataset collection. Created in BioRender: https://BioRender.com/z293vp2.

The distribution of samples across the DNA microarray and RNA-Seq technologies are showed in Fig. 2A. IPF was the most represented disease across all the collected samples (49.1 %), followed by hypersensitivity pneumonitis (8.75 %, Fig. 2B). Collected datasets encompass lung biopsy, fibroblast, macrophage, alveolar epithelial, and bronchoalveolar lavage (BAL) samples.

**Fig. 3:**
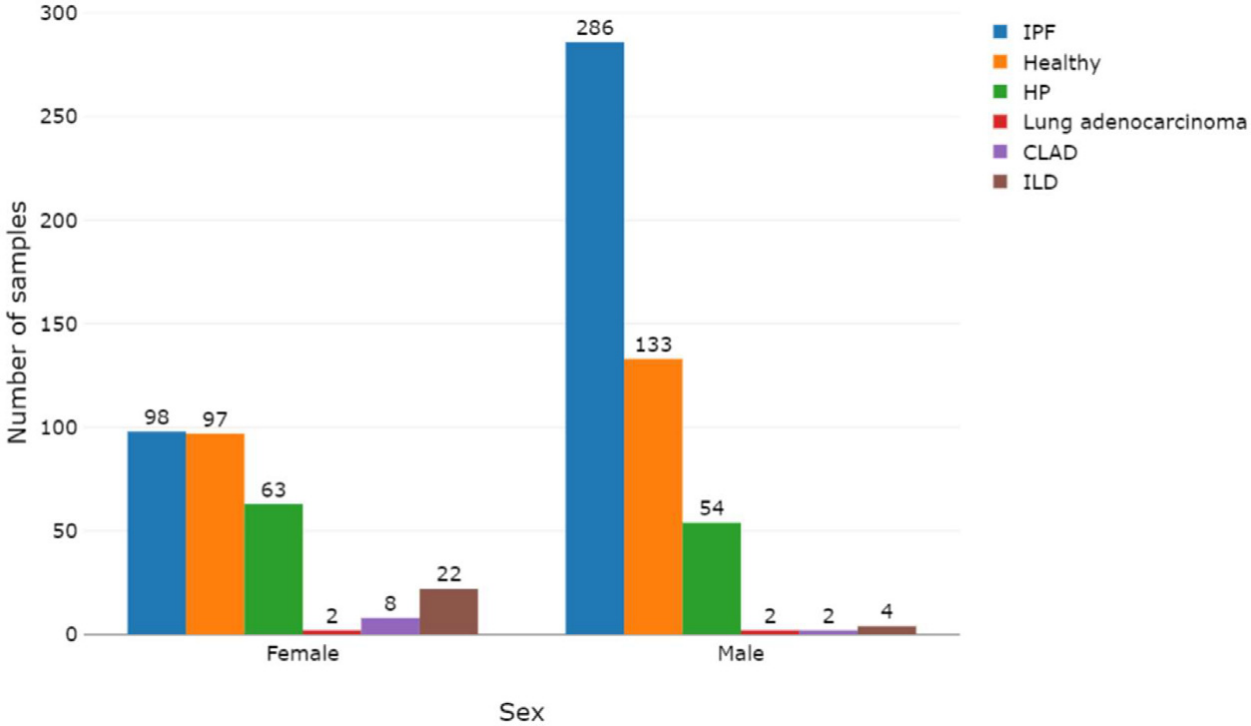
Sample distribution across sexes and conditions when the information was available. Created in BioRender: https://BioRender.com/3767wks.

The progression of IPF is tightly correlated with the sex, with males experiencing higher mortality and earlier hospitalization and being more frequently represented in the IPF population compared to females [31,32]. Advanced age has also been found to be associated with poor prognosis in IPF [31]. Fig. 3 illustrates the distribution of samples among sexes across various ILDs and healthy counterparts when data was accessible. The figure highlights a predominant representation of males in the dataset, particularly evident in IPF cases.

**Fig. 4:**
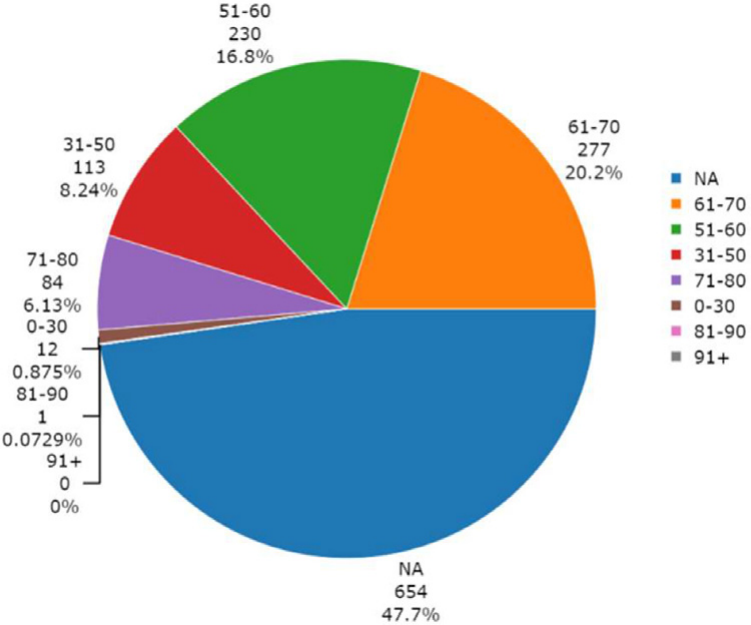
Distribution of samples across age groups. NA indicates samples where the information was not available. Created in BioRender: https://BioRender.com/vlcz7n4.

Based on the available data, Fig. 4 displays the distribution of samples among age groups across different ILDs. The figure emphasizes that while the prevalence of IPF increases with age, higher mortality rates result in lower prevalence among the most advanced age groups.

The complexity of the disease makes IPF well-suited for network-based modelling by systems biology and pharmacology approaches that comprehend the intricate interactions of biomolecules. By integrating large-scale biological data and considering the interconnectedness of various signalling cascades, these methodologies offer a holistic understanding of disease pathogenesis, facilitating the identification of potential therapeutic targets and the development of more effective treatment strategies. Our recent integrative network analysis, showcased in [33], shows the potential of integrating DNA microarray and RNA-seq data in a unified meta-analysis. In this analysis, the topological properties of the co-expression networks of biopsies from IPF patients and healthy counterparts, as reported in the present work, are exploited to identify functionally meaningful gene modules, to reveal both known and novel disease mechanisms. On one hand, the resulting gene modules highlight known dysregulated pathways in IPF, such as antigen presentation and processing, emphasizing the role of immune system activation in fibrosis, and platelet activation, neutrophil degranulation, and extracellular compartment alterations, suggesting a strong inflammatory response and extracellular matrix remodelling, both widely associated with disease development. These components provide structural support for cells, ensuring mechanical stability and tissue elasticity essential for normal lung function. On the other hand, novel functionalities emerged from the analysis of the integrated network modules. For instance, mitochondrial dysfunction, previously underappreciated, emerged as a key determinant of pulmonary fibrosis severity, aligning with recent findings linking mitochondrial quality to disease progression. Additional pathways identified include interleukin signaling (notably IL-4 and IL-13), interferon responses, CD28 activation, and nonsense-mediated mRNA decay (NMD), implicating RNA surveillance in disease mechanisms. For more information about the results of this analysis, please refer to [33]. These results demonstrate that the co-expression network models developed in this study are a robust tool for investigating the molecular landscape of IPF, enabling the identification of candidate biomarkers for disease diagnosis, progression, and treatment response. Additionally, they shed light on the intricate regulatory circuits and offer promising avenues for therapeutic intervention and dysregulated pathways underlying IPF pathogenesis, facilitating the discovery of novel therapeutic targets and the development of targeted therapies.

## 4 Experimental Design, Materials and Methods

### 4.1 Metadata harmonisation

The curation and harmonisation of metadata were performed with the ESPERANTO software [34]. ESPERANTO is designed for efficient semi-supervised curation tasks on omics metadata. The user actively participates in the decision process while harmonising data within a consistent framework and enhancing data FAIRness. This approach combines the benefits of both automated and manual curation methods. The data models, to which the raw metadata were mapped, are reported in the data dictionary file (enclosed with the preprocessed data). The data dictionary describes all the allowed variables, variable synonyms, allowed features, and feature synonyms reported in the final metadata tables. At the same time, this work aims to homogenise the preprocessing procedures to improve the comparability of gene expression data across different studies and platforms. Metadata visualization in Fig. 2, Fig. 3, Fig. 4 was created using the plotly 4.10.1 R-package [35].

### 4.2 DNA microarray data

*Data collection and homogenization*. DNA microarray generated transcriptomics data were retrieved from NCBI GEO repository by using the GEOquery [36] R package. For each dataset, a metadata table specifying the phenotype (i.e. IPF, healthy) and the biological system (i.e. biopsy, fibroblast, macrophage, epithelial, BAL), along with other phenotypic information, was also retrieved. The DNA microarray datasets with the GEO identifiers are reported in Table 1. The samples from patients with UIP were included in the IPF group, as these two diseases are often used synonymously.

*Data quality check.* The retrieved datasets were evaluated by visual inspection of the quality check reports and multi-dimensional scaling (MDS) plots using the eUTOPIA software [37]. Furthermore, in the case of Affymetrix datasets, identification of outlier samples were identified by using the Normalized Unscaled Standard Error (NUSE) and the Relative Log Expression using the affyPLM v1.78.0 [38] R package and the RNA degradation curves (RNADeg) through the affy v1.76.0 R-package [39]. The Agilent quality control report was generated by using arrayQualityMetrics v3.54.0 [40].

*Normalisation.* Data normalisation was carried out by using the eUTOPIA software. The normalisation for Affymetrix-based studies was performed by using the *justRMA* from the R affy v1.76.0 package [41]. Normalisation of Agilent-based studies was performed with the *normalizeQuantiles* function from the limma v3.54.0 package [42].

*Surrogate variable analysis.* To investigate the effect of unknown technical variables that might conceal biological variability, Surrogate Variable Analysis (SVA) was performed with the eUTOPIA software, which implements the sva v3.46.0 R package [43]. The analysis was performed by using the disease as a variable of interest. The other biological variables were used as covariates if present and not confounded with the variable of interest.

*Probe annotation*. In eUTOPIA, the analysis was performed with the raw data for Agilent datasets and the expression matrix was aggregated by computing the median of the expression of the Agilent probes mapping to the same probe name. For Affymetrix-based microarrays custom annotation files (CDF files) were downloaded from Brainarray (http://brainarray.mbni.med.umich.edu/Brainarray/Database/

CustomCDF/25.0.0/ensg.asp). Probe annotation was performed using biomaRt v.2.40.1. The probe names in expression matrices and differential gene expression tables were annotated to the Ensembl gene IDs and Gene Symbols using R. Gene names were grouped based on unique values with dplyr 1.0.2 package. Subsequently, the median of the expression value for each sample was calculated within each gene. This resulted in the creation of a new data frame, where each row represents a unique gene, and the columns contain the computed median values for the respective expression value associated with each gene. The analytical steps for processing Affymetrix DNA microarrays are illustrated in Supplementary Fig. 4, while the analytical processing steps for Agilent DNA microarrays are represented in Supplementary Fig. 5. Supplementary Figs. 4–7 are created with Biorender.com.

### 4.3 RNA-seq data

*Data collection and homogenization*. Raw files in “*.fastq*” format were obtained from the European Nucleotide Archive (ENA - https://www.ebi.ac.uk/ena/browser/home). In case the raw data were unavailable, we retrieved the normalised expression matrix from GEO, ensuring that the read alignment and normalisation were compatible with our pipeline. In addition to the raw data files, the metadata tables containing the sample-wise clinical features for each dataset were also collected using the GEOquery R package. RNA-seq datasets with their GEO identifiers are reported in Table 2. As with the DNA microarray data, the samples from patients with usual UIP were included in the IPF group, as these two diseases are often used synonymously.

*Quality control.* Quality assessments were conducted on RNA-Seq datasets utilizing FastQC v0.11.7 (https://www.bioinformatics.babraham.ac.uk/projects/fastqc/). Trimming of reads for low-quality ends in addition to adapter removal was performed by Cutadapt version 3.7. In particular, the Phred score threshold for trimming was set to 20, and the minimum read length for 60 nucleotides. The adapter-clipped trimmed raw files were further quality-checked with FastQC v0.11.7.

*Read alignment.* Subsequently, RNA-seq reads were aligned against the human reference genome assembly GRCh38. The alignment was performed using the HISAT2 algorithm [44] utilising the genome indexes built for usage with HISAT2 (retrieved from https://ccb.jhu.edu/software/hisat2/manual.shtml).

*Read counts extraction.* Transcript quantification was performed by using the *featureCounts* function from the Rsubread v1.34.4 R package [45]. To accomplish this task, the Ensembl version 108 annotation was downloaded from http://www.ensembl.org and then utilised for read count extraction.

*Low counts filtering.* To exclude the transcripts with low expression levels across all samples within each dataset, the proportion test strategy was employed as implemented in the function *filtered.data* of the R package NOISeq [46].

*Gene annotation:* The Ensembl gene IDs were annotated to gene symbols using biomaRt v.2.40.1. Gene names were grouped based on unique values with dplyr 1.0.2 package. As with the DNA microarray datasets, the median of the expression value was calculated for each sample within each gene. This process resulted in a new data frame, where each row represents a unique gene, and the columns contain the computed median values for the respective expression value associated with each gene.

### 4.4 Differential gene expression analysis

***DNA microarrays.*** Differential gene expression analysis of DNA microarray platforms was performed with eUTOPIA, which applies linear model implementation from the R-package limma. The lmFit function from the limma R-package fits gene-wise linear models to the microarray data. The variable of interest in the model was the diagnosis (disease vs. healthy), and other relevant biological and technical variables (if present and if not confounded with the variable of interest) were used as covariates. Also, comparisons between different lung locations, disease severity states and disease subtypes were performed if data were available. eUTOPIA applies eBayes function to assess differential expression by using the fitted model with the contrast coefficients. The p-value adjustment was carried out with the “Benjamini & Hochberg” method. The differential gene expression results were filtered by considering adjusted p-values less than or equal to 0.01 and absolute log-fold changes greater than or equal to 0.58.

***RNA-seq.*** Normalisation and differential gene expression analysis of RNA-seq data was carried out by using DESeq2 1.24.0 [47]. The variable of interest in the model was the diagnosis (disease vs. healthy) and other relevant biological and technical variables were used as covariates when available and not confounded with the variable of interest. Moreover, comparisons between distinct lung locations and disease severity states as well as ILD subtypes were conducted when the data was present. The differential gene expression results underwent a similar filtering process as the microarray results, involving the consideration of adjusted p-values less than or equal to 0.01 and absolute log-fold changes greater than or equal to 0.58. The preprocessing pipeline of the RNA-seq data is illustrated in Supplementary Fig. 6.

### 4.5 Gene co-expression networks

*Dataset integration and batch effect mitigation:* In order to infer co-expression networks representing each of the IPF and healthy biological systems (biopsies, BAL, fibroblasts, macrophages, epithelial cells), datasets were aggregated by selecting common genes across the platforms. Batch effect deriving from the different origin of the datasets was mitigated through the pamR package.

*Network inference:* To infer the networks from the batch-adjusted expression matrices, INfORM functions were utilised for the network inference [48]. The “*get_ranked_consensus_matrix*” function was applied to determine the correlations between genes, based on their expression levels, for both RNA-seq and microarray and for disease and healthy samples independently. The CLR algorithm was carried out for network inference and Pearson correlation was employed as the correlation method. To set a threshold on the edges to be included in the networks, the “*parse_edge_rank_matrix*” function. This function ranks the edges based on their weight (represented by Pearson correlation measures) and then systematically adds edges from the top of the rank until all the nodes within the network are connected. The networks were then converted to igraph objects using the *“get_iGraph”* function. For each network, centrality measures were calculated, including degree, betweenness, closeness, clustering coefficient and eigenvector. Subsequently, such centrality measures were aggregated through the Borda function from the TopKLists package and a global centrality-based gene rank was computed. The analytical steps of network inference is represented in Supplementary Fig. 7.

## Limitations

All the datasets included in this study encompass metadata files, reporting sample-wise technical and clinical information such as technology, platform, sampling site, gender, age or disease state. Metadata were remarkably heterogeneous across the datasets. This is a common problem when dealing with high-dimensional omics data, that hampers their integrability. In this study, the compliance of the data with FAIR principles was increased, facilitating their usage in future efforts of the scientific community. Moreover, batch effect can extensively affect the results of omics data, leading to artifacts in the results and their interpretation. This effect can derive from protocols, reagents, equipment, laboratory conditions, sample collection, storage, preparation methods, and time. Since gene expression datasets presented in this manuscript derive from different studies, it is essential to mitigate the batch effect to accurately compute differentially expressed genes and infer network relationships. Therefore, a rigorous mitigation of batch effect was carried out by considering the diverse sources of gene expression datasets as a batch variable, with the aim to integrate datasets produced with the same technology and platform to ultimately infer network relationships. PCA plots illustrating sample distribution pre- and post-batch effect mitigation are provided in supplementary materials.

## Ethics Statement

The authors have read and follow the ethical requirements for publication in Data in Brief and confirm that the current work does not involve human subjects, animal experiments, or any data collected from social media platforms.

## CRediT authorship contribution statement

**Simo Inkala:** Data curation, Conceptualization, Writing – original draft, Writing – review & editing. **Antonio Federico:** Supervision, Conceptualization, Writing – original draft, Writing – review & editing. **Angela Serra:** Methodology, Writing – original draft, Writing – review & editing. **Dario Greco:** Supervision, Conceptualization, Funding acquisition.

## Data Availability

ZenodoCurated and harmonised transcriptomics datasets of interstitial lung disease patients (Reference data)

## Appendix Supplementary materials

Image, application 1
