# Supplementary material for: Curated and harmonised transcriptomics datasets of interstitial lung diseases
Source: Data Brief. 2025 Oct 14;63:112139. doi: 10.1016/j.dib.2025.112139 (PMC12581653; doi:10.1016/j.dib.2025.112139)
Supplement: Supplementary file 1 [file mmc1.zip › Supplementary_material/DNA-microarray/GSE21369/GSE21369_eUTOPIA_Affymetrix_QC_Report_2024-02-09.pdf]

eUTOPIA Affymetrix QC Report

eUTOPIA

Contents

1 Outliers Table **1**
- 1.1 Outliers (All Methods) . . . 1
- 1.2 Outliers (At Least One Method) . . . 1

2 RNA Degradation **2**
- 2.1 Summarized Mean QC . . . 2
- 2.2 Discrete QC Plots . . . 3

3 Relative Log Expression **4**
- 3.1 Summarized Median QC . . . 4
- 3.2 Discrete QC Plots . . . 5

4 Normalized Unscaled Standard Errors **6**
- 4.1 Summarized Median QC . . . 6
- 4.2 Discrete QC Plots . . . 7

5 YAQC Plots **8**

1 Outliers Table

	RLE	NUSE	DEG	SUM
ILD_4	0	0	1	1
ILD_5	0	0	1	1
ILD_13	0	0	1	1
ILD_16	0	0	1	1
ILD_17	0	0	1	1
ILD_18	0	0	1	1
ILD_23	0	0	1	1
Control_5	1	0	0	1
ILD_1	1	1	0	2
ILD_2	1	0	0	1
ILD_7	1	1	0	2
ILD_9	1	1	0	2

1.1 Outliers (All Methods)

Outliers overall
ILD_1
ILD_7
ILD_9

1.2 Outliers (At Least One Method)

Outliers at least 1
ILD_4
ILD_5
ILD_13
ILD_16
ILD_17
ILD_18
ILD_23
Control_5
ILD_1
ILD_2
ILD_7
ILD_9

2 RNA Degradation

2.1 Summarized Mean QC

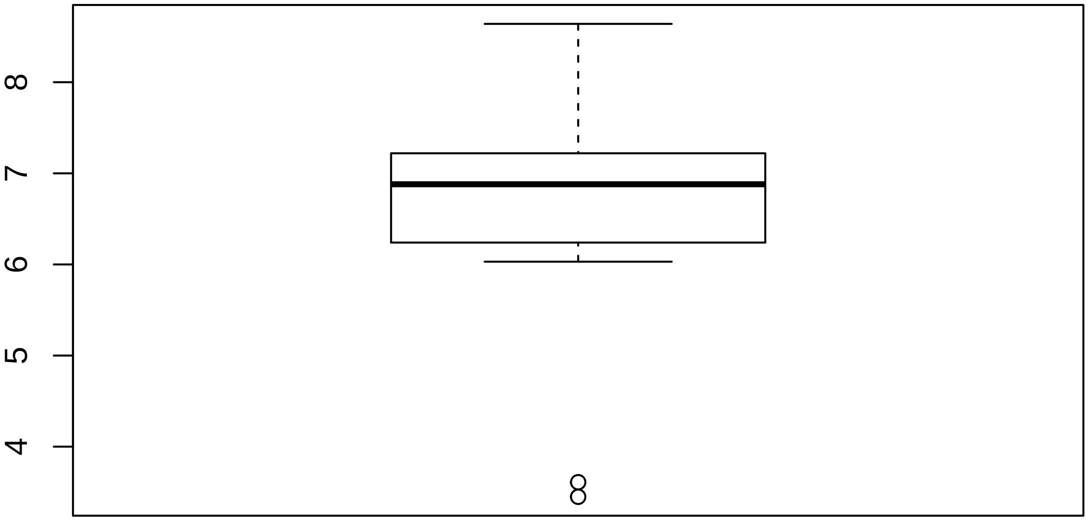

2.2 Discrete QC Plots

Sample Group [1]

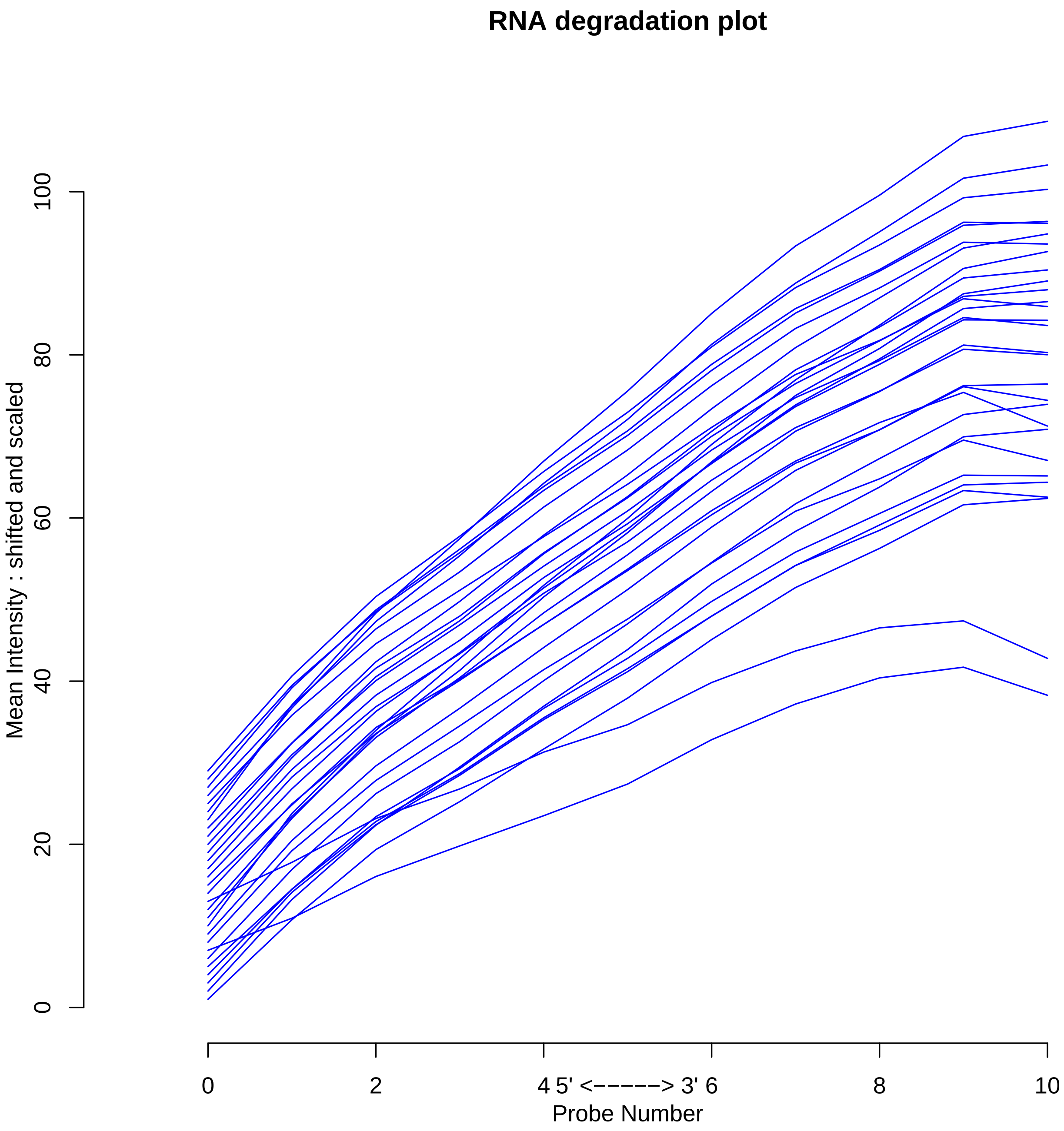

3 Relative Log Expression

3.1 Summarized Median QC

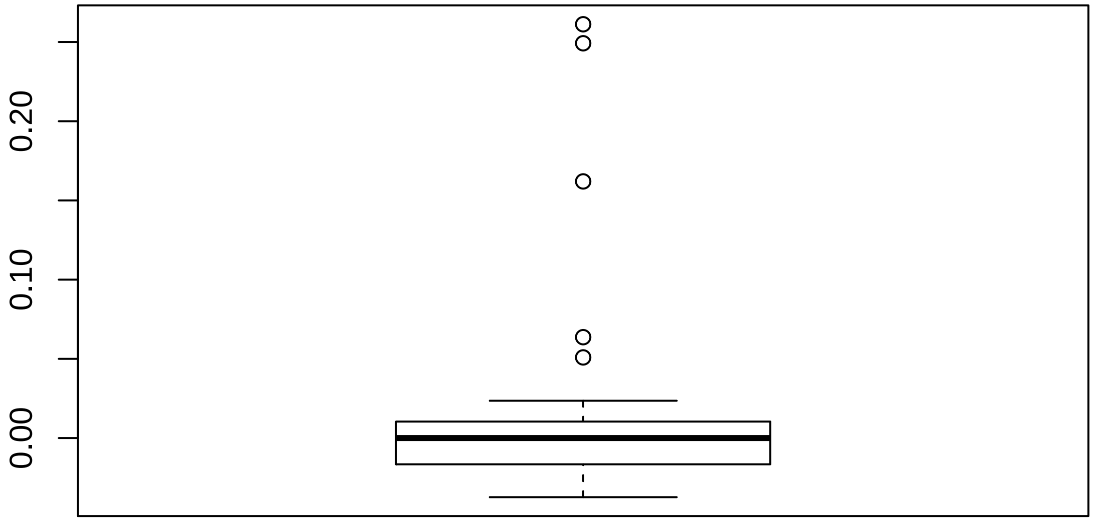

3.2 Discrete QC Plots

Sample Group [1]

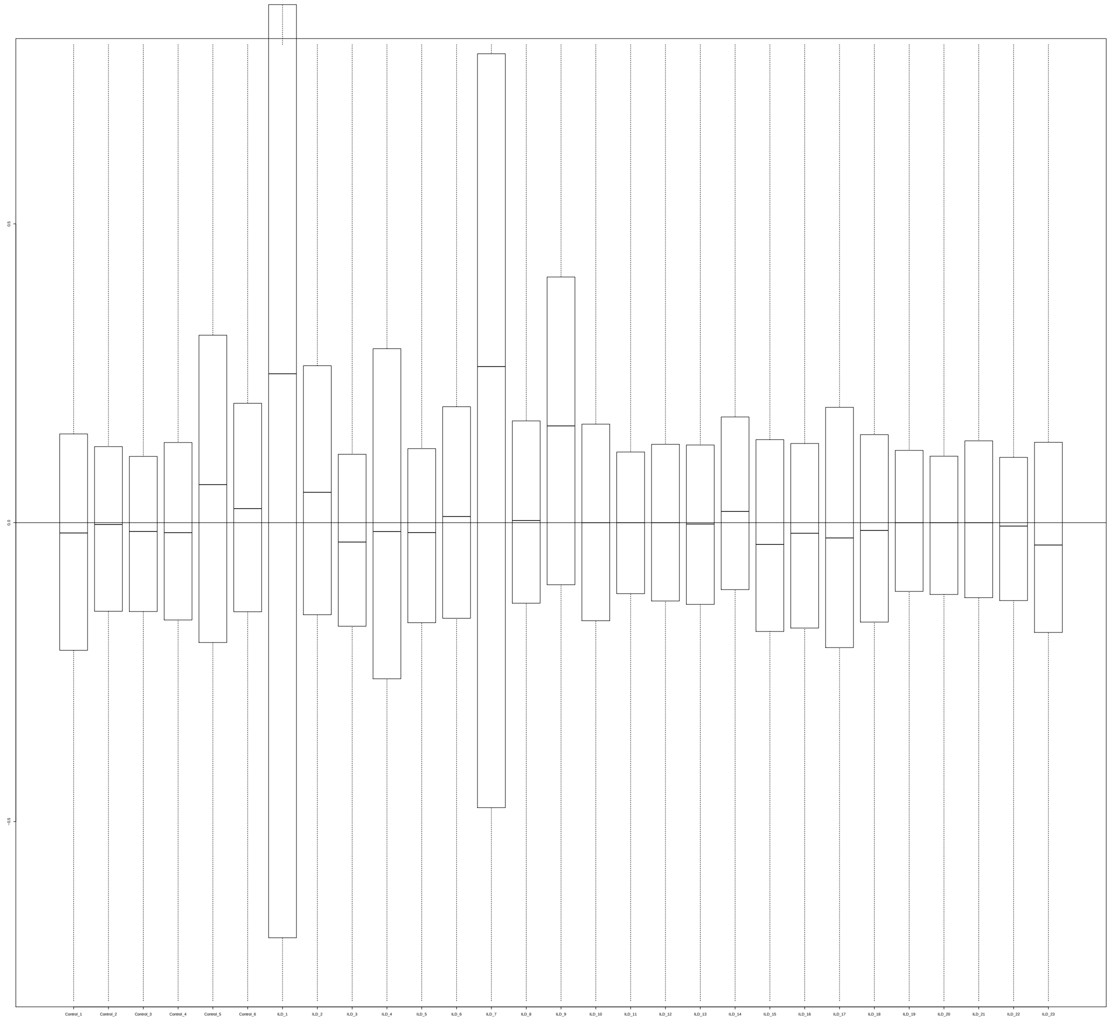

4 Normalized Unscaled Standard Errors

4.1 Summarized Median QC

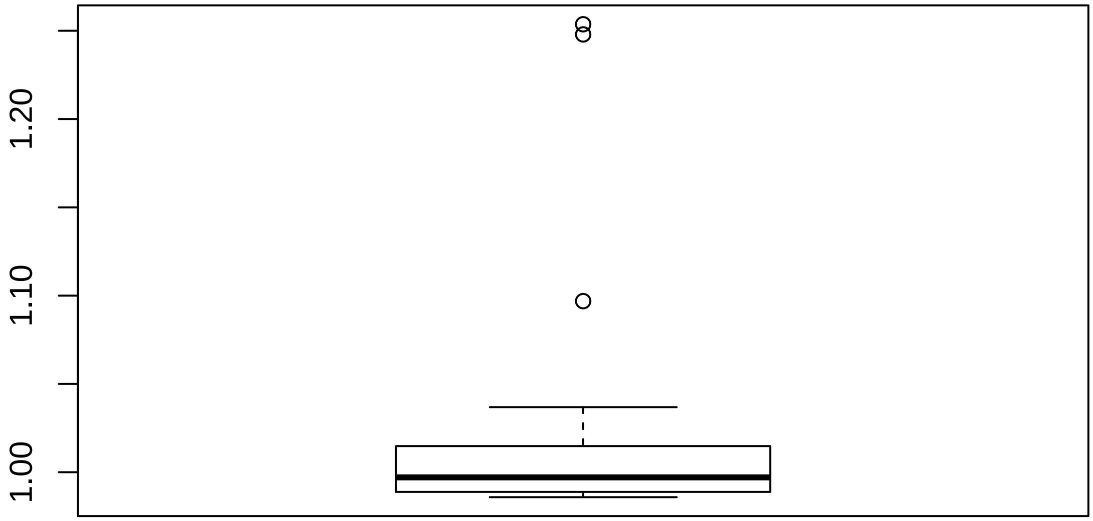

4.2 Discrete QC Plots

Sample Group [1]

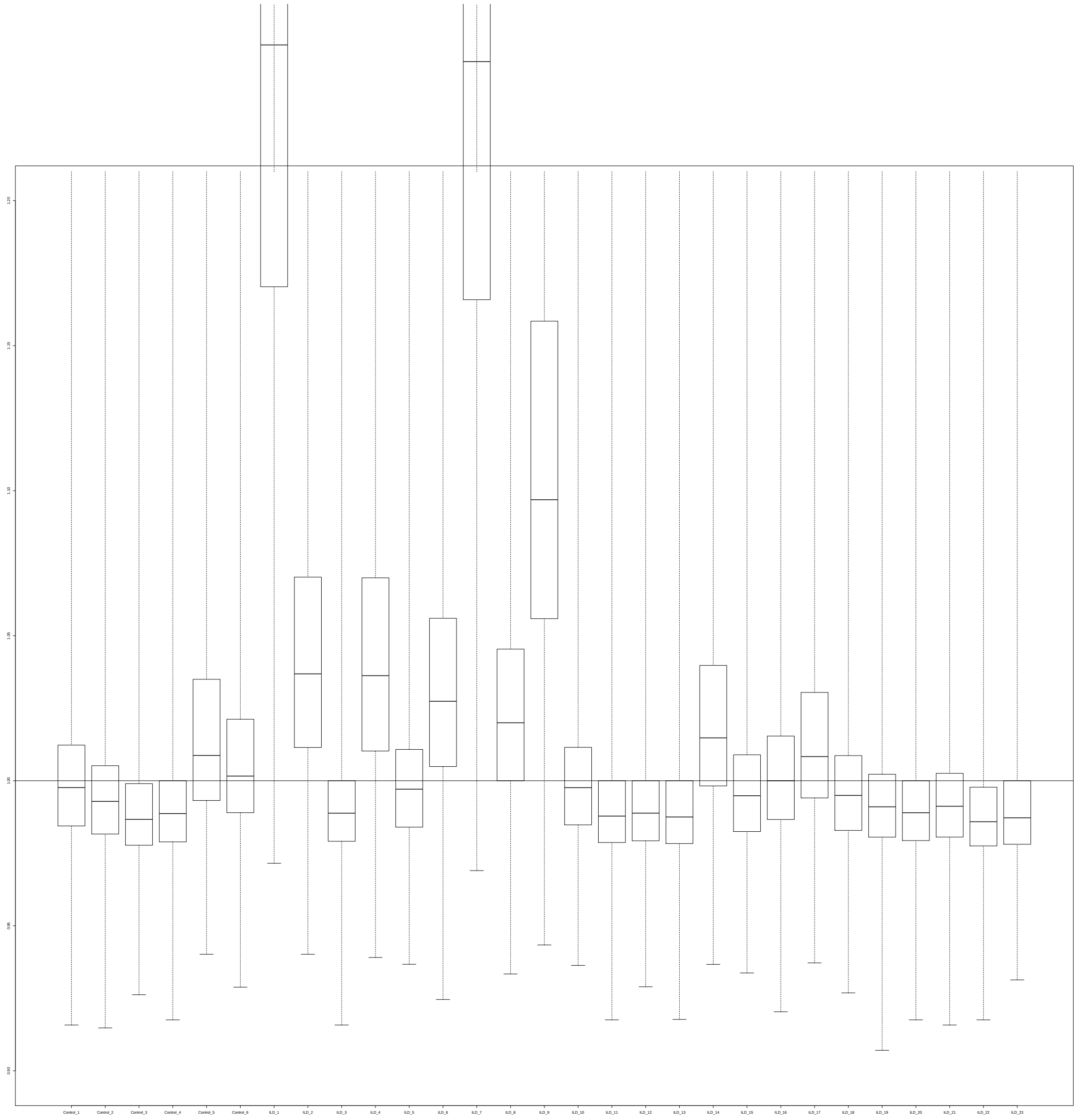

5 YAQC Plots

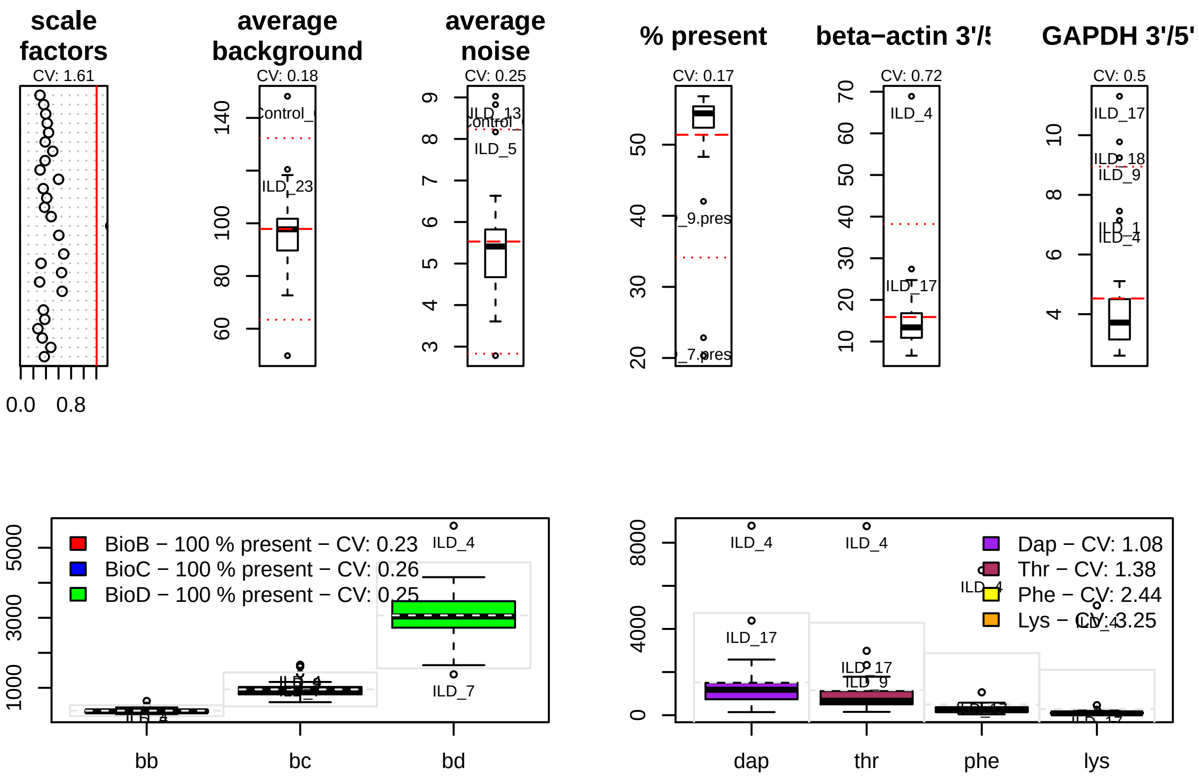
scale factors
CV: 1.61
average background
CV: 0.18
average noise
CV: 0.25
% present
CV: 0.17
beta−actin 3'/5'
CV: 0.72
GAPDH 3'/5'
CV: 0.5
BioB − 100 % present − CV: 0.23
BioC − 100 % present − CV: 0.26
BioD − 100 % present − CV: 0.25
bb
bc
bd
Dap − CV: 1.08
Thr − CV: 1.38
Phe − CV: 2.44
Lys − CV: 3.25
dap
thr
phe
lys
