# Supplementary material for: Curated and harmonised transcriptomics datasets of interstitial lung diseases
Source: Data Brief. 2025 Oct 14;63:112139. doi: 10.1016/j.dib.2025.112139 (PMC12581653; doi:10.1016/j.dib.2025.112139)
Supplement: Supplementary file 1 [file mmc1.zip › Supplementary_material/DNA-microarray/GSE11196/GSE11196_eUTOPIA_Affymetrix_QC_Report_2024-02-12.pdf]

eUTOPIA Affymetrix QC Report

eUTOPIA

Contents

1 Outliers Table **1**
- 1.1 Outliers (All Methods) 1
- 1.2 Outliers (At Least One Method) 2

2 RNA Degradation **2**
- 2.1 Summarized Mean QC 2
- 2.2 Discrete QC Plots 3

3 Relative Log Expression **4**
- 3.1 Summarized Median QC 4
- 3.2 Discrete QC Plots 5

4 Normalized Unscaled Standard Errors **6**
- 4.1 Summarized Median QC 6
- 4.2 Discrete QC Plots 7

5 YAQC Plots **8**

1 Outliers Table

	RLE	NUSE	DEG	SUM
Control__(Control128)__Contractile__Heavy__polyribosomal__RNA	0	0	1	1
IPF__(IPF129)__Contractile__Heavy__polyribosomal__RNA	0	0	1	1
IPF__(IPF75)__Contractile__Heavy__polyribosomal__RNA	0	0	1	1
Control__(Control128)__Non__contractile__Heavy__polyribosomal__RNA	0	0	1	1
Control__(Control106)__Contractile__Total__RNA	0	0	1	1
Control__(Control128)__Contractile__Total__RNA	0	0	1	1
IPF__(IPF75)__Contractile__Total__RNA	0	0	1	1
Control__(Control106)__Non__contractile__Total__RNA	0	0	1	1
Control__(Control128)__Non__contractile__Total__RNA	0	0	1	1
Control__(Control54)__Non__contractile__Total__RNA	0	0	1	1
IPF__(IPF129)__Non__contractile__Total__RNA	0	0	1	1
IPF__(IPF75)__Non__contractile__Total__RNA	0	0	1	1
IPF__(IPF12)__Contractile__Heavy__polyribosomal__RNA	1	0	0	1
IPF__(IPF14)__Contractile__Heavy__polyribosomal__RNA	1	0	0	1
Control__(Control54)__Non__contractile__Heavy__polyribosomal__RNA	1	0	0	1
IPF__(IPF12)__Non__contractile__Heavy__polyribosomal__RNA	0	1	0	1
Control__(Control89)__Contractile__Total__RNA	0	1	0	1
IPF__(IPF12)__Contractile__Total__RNA	0	1	0	1

1.1 Outliers (All Methods)

Outliers overall
NA

1.2 Outliers (At Least One Method)

Outliers at least 1
Control_(Control128)_Contractile_Heavy_polyribosomal_RNA
IPF_(IPF129)_Contractile_Heavy_polyribosomal_RNA
IPF_(IPF75)_Contractile_Heavy_polyribosomal_RNA
Control_(Control128)_Non_contractile_Heavy_polyribosomal_RNA
Control_(Control106)_Contractile_Total_RNA
Control_(Control128)_Contractile_Total_RNA
IPF_(IPF75)_Contractile_Total_RNA
Control_(Control106)_Non_contractile_Total_RNA
Control_(Control128)_Non_contractile_Total_RNA
Control_(Control54)_Non_contractile_Total_RNA
IPF_(IPF129)_Non_contractile_Total_RNA
IPF_(IPF75)_Non_contractile_Total_RNA
IPF_(IPF12)_Contractile_Heavy_polyribosomal_RNA
IPF_(IPF14)_Contractile_Heavy_polyribosomal_RNA
Control_(Control54)_Non_contractile_Heavy_polyribosomal_RNA
IPF_(IPF12)_Non_contractile_Heavy_polyribosomal_RNA
Control_(Control89)_Contractile_Total_RNA
IPF_(IPF12)_Contractile_Total_RNA

2 RNA Degradation

2.1 Summarized Mean QC

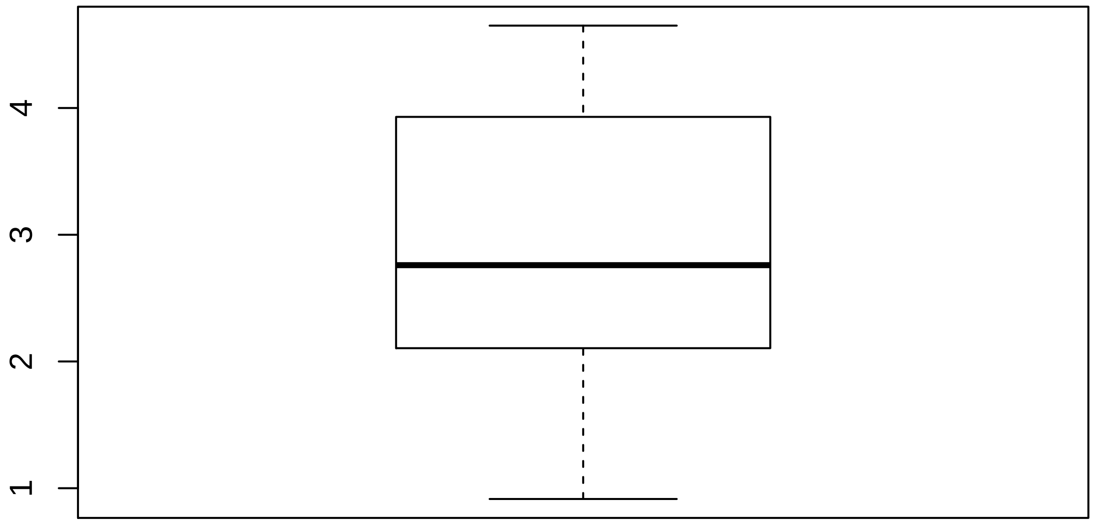

2.2 Discrete QC Plots

Sample Group [1]

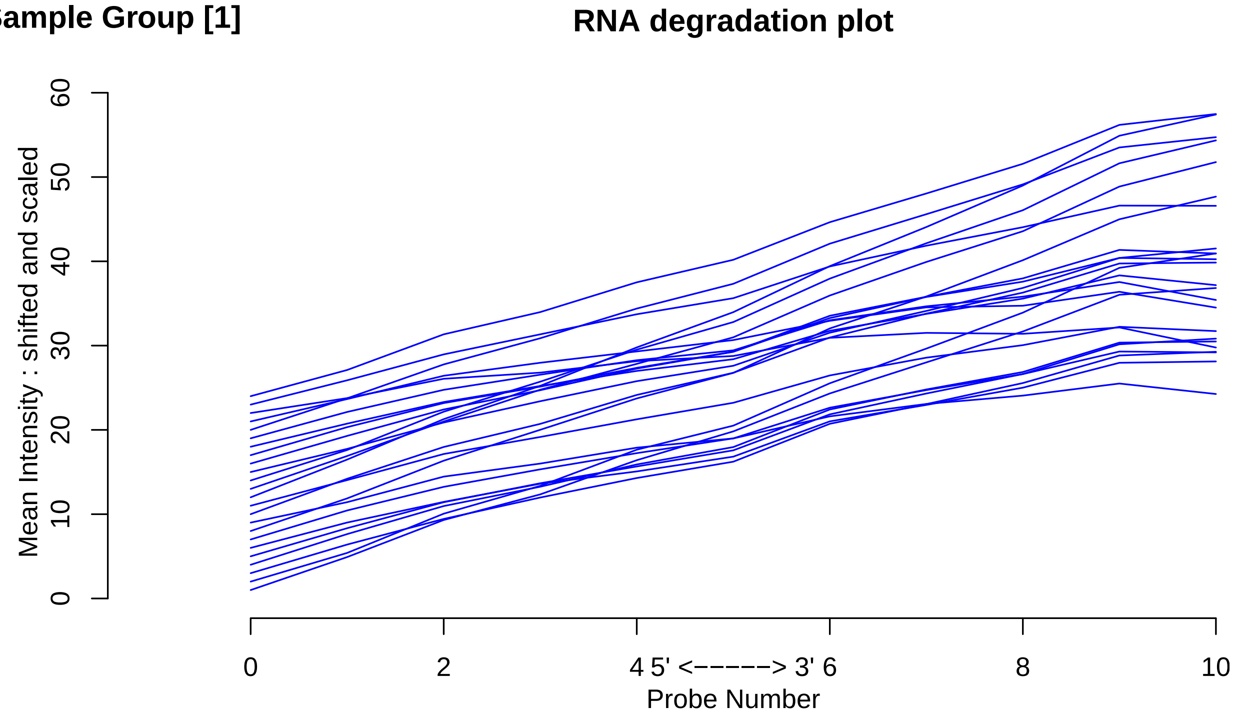

Sample Group [2]

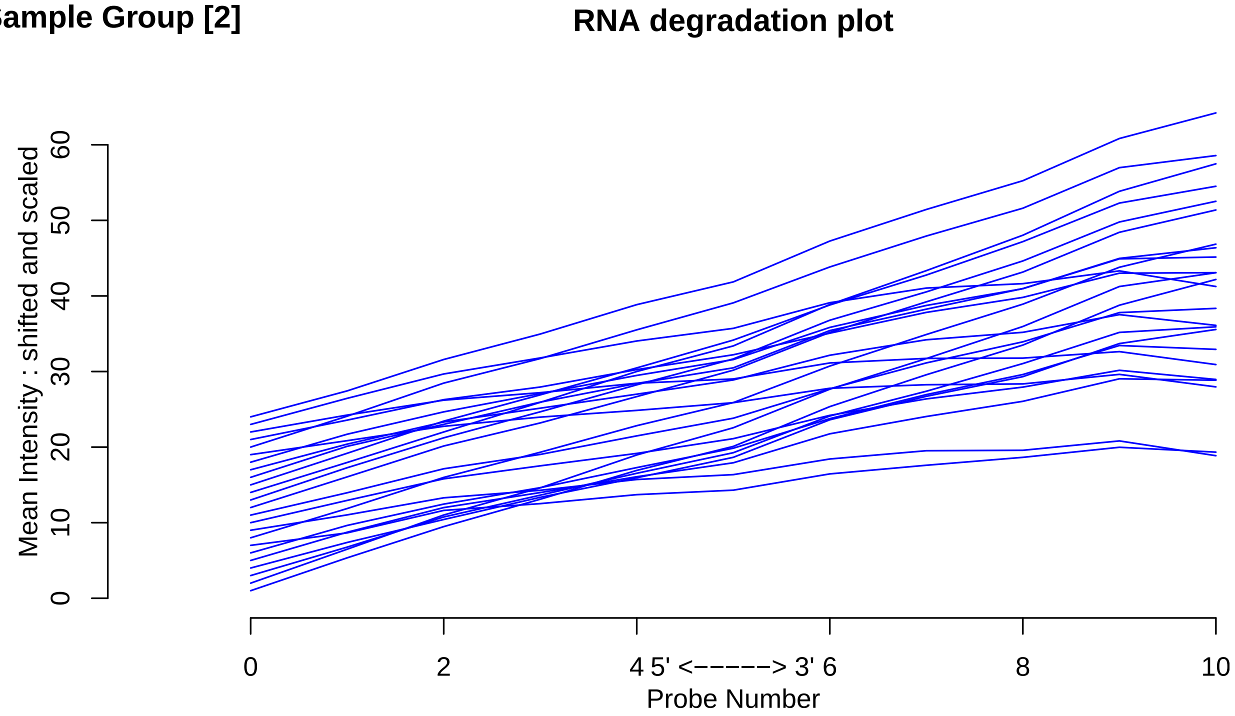

3 Relative Log Expression

3.1 Summarized Median QC

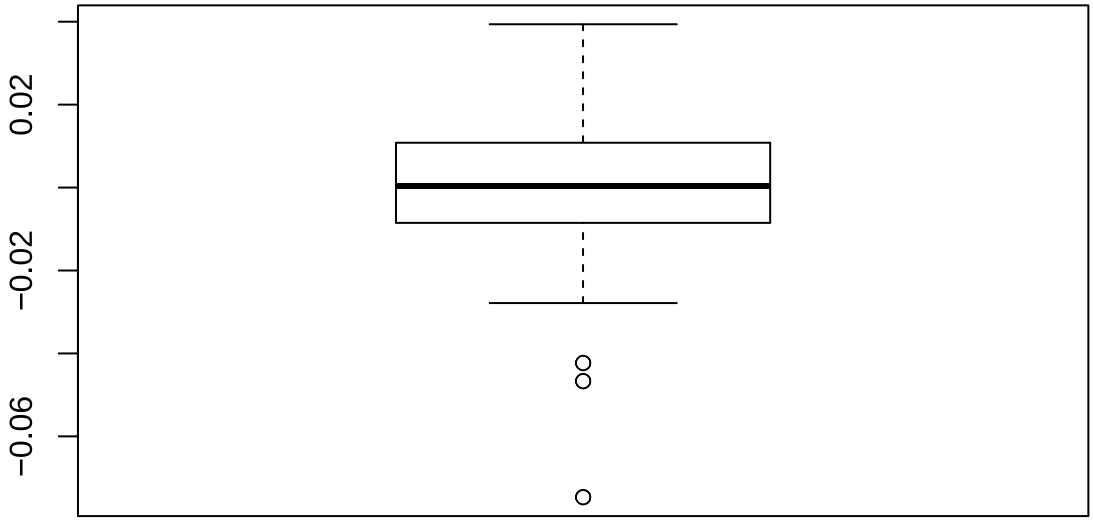

3.2 Discrete QC Plots

Sample Group [1]

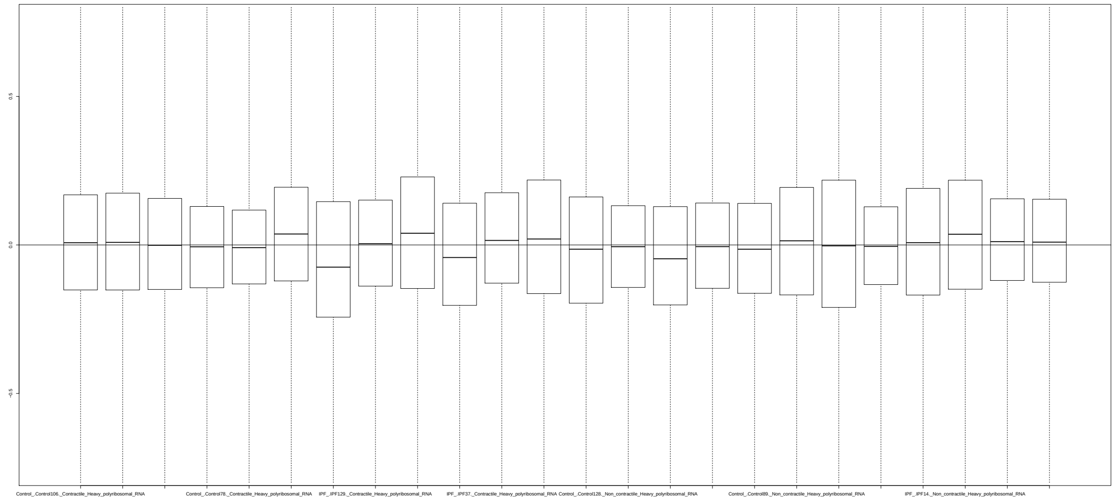

Sample Group [2]

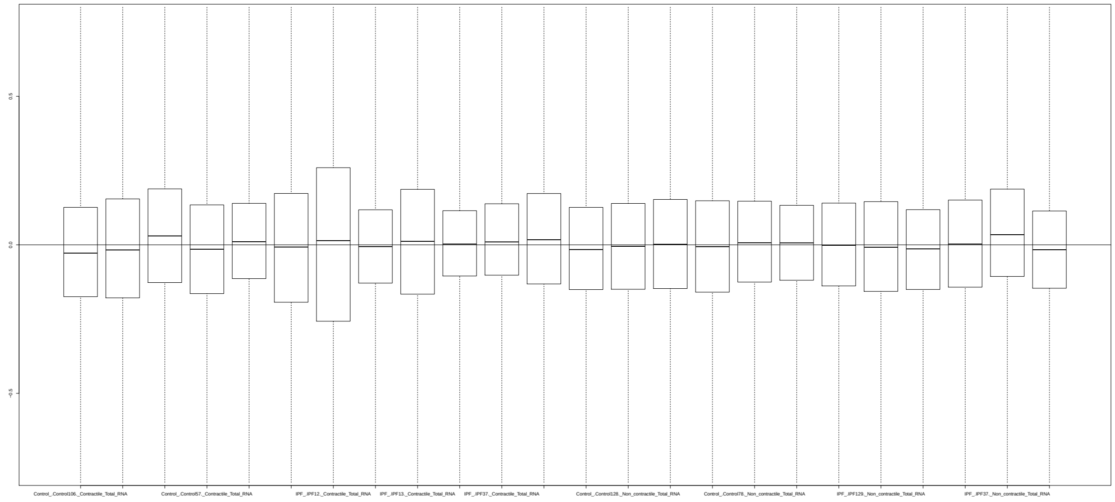

4 Normalized Unscaled Standard Errors

4.1 Summarized Median QC

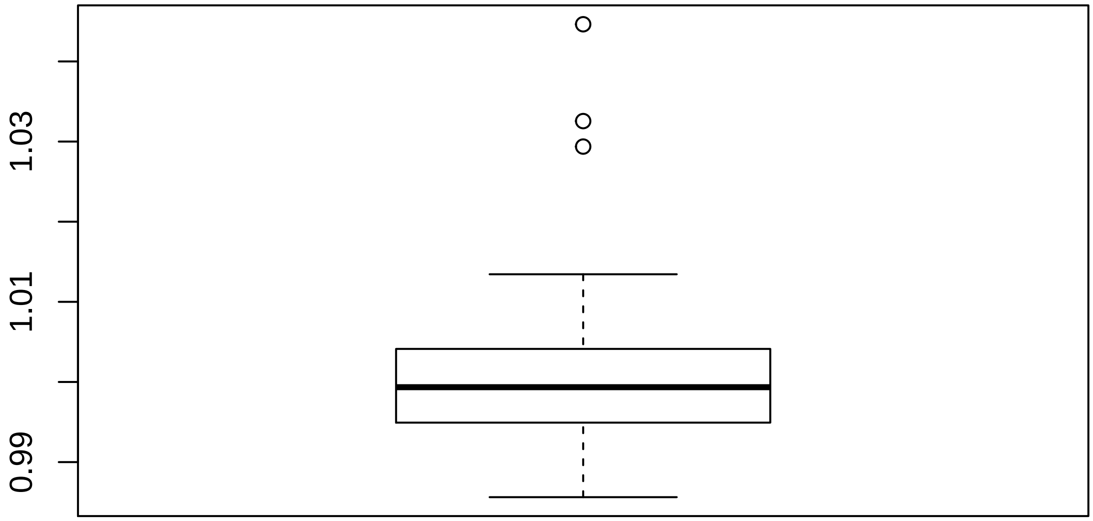

4.2 Discrete QC Plots

Sample Group [1]

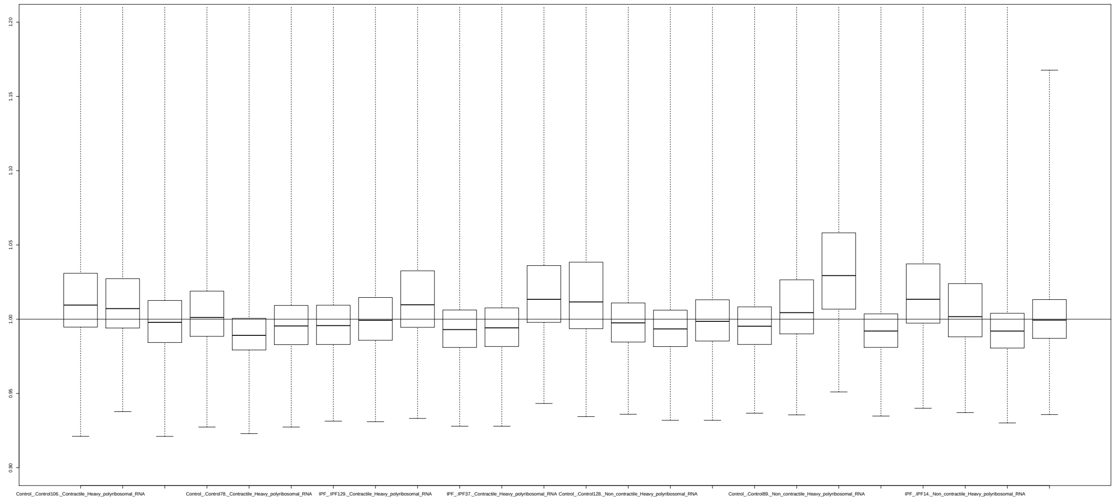

Sample Group [2]

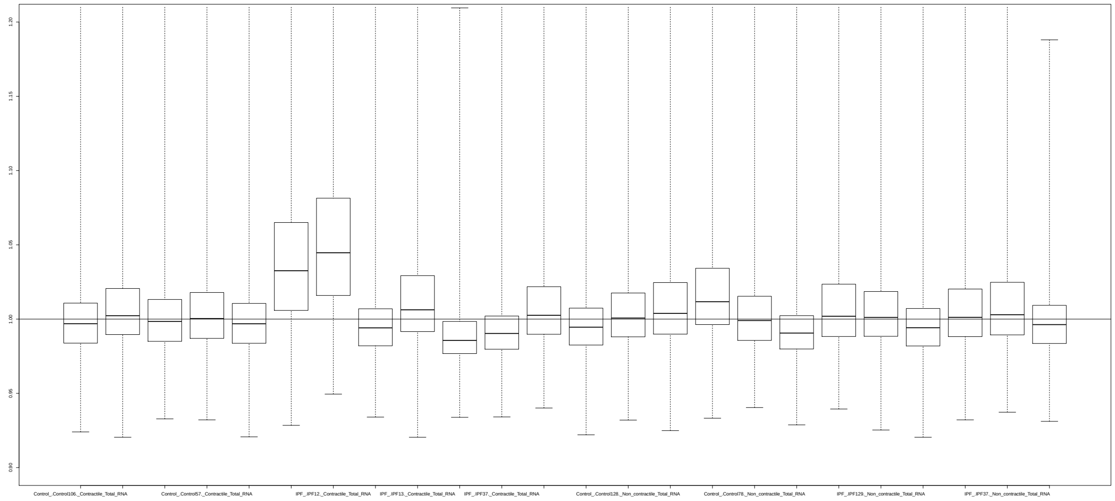

5 YAQC Plots

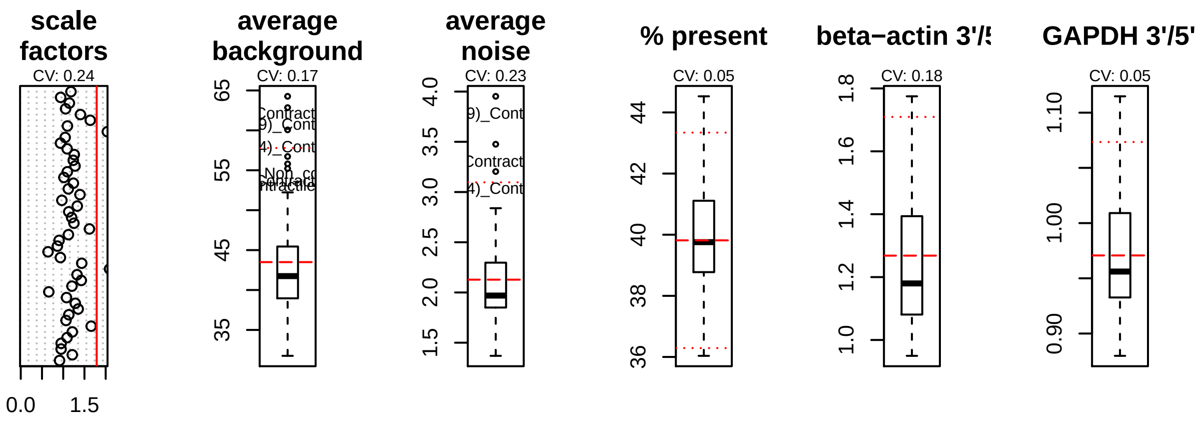
scale factors
CV: 0.24
0.0
1.5
average background
CV: 0.17
average noise
CV: 0.23
% present
CV: 0.05
beta−actin 3'/5'
CV: 0.18
GAPDH 3'/5'
CV: 0.05

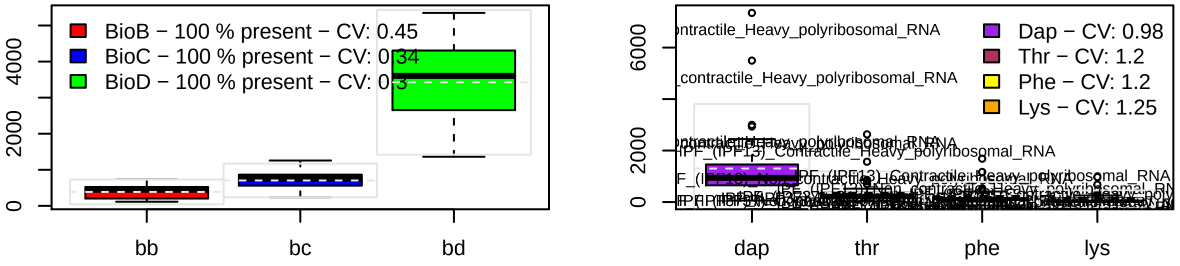
BioB − 100 % present − CV: 0.45
BioC − 100 % present − CV: 0.34
BioD − 100 % present − CV: 0.3
bb
bc
bd
Dap − CV: 0.98
Thr − CV: 1.2
Phe − CV: 1.2
Lys − CV: 1.25
dap
thr
phe
lys
