# Supplementary material for: Curated and harmonised transcriptomics datasets of interstitial lung diseases
Source: Data Brief. 2025 Oct 14;63:112139. doi: 10.1016/j.dib.2025.112139 (PMC12581653; doi:10.1016/j.dib.2025.112139)
Supplement: Supplementary file 1 [file mmc1.zip › Supplementary_material/DNA-microarray/GSE24206/GSE24206_eUTOPIA_Affymetrix_QC_Report_2024-02-12.pdf]

eUTOPIA Affymetrix QC Report

eUTOPIA

Contents

1 Outliers Table **1**
1.1 Outliers (All Methods) 1
1.2 Outliers (At Least One Method) 2

2 RNA Degradation **2**
2.1 Summarized Mean QC 2
2.2 Discrete QC Plots 3

3 Relative Log Expression **4**
3.1 Summarized Median QC 4
3.2 Discrete QC Plots 5

4 Normalized Unscaled Standard Errors **6**
4.1 Summarized Median QC 6
4.2 Discrete QC Plots 7

5 YAQC Plots **8**

1 Outliers Table

	RLE	NUSE	DEG	SUM
Early__IPF__surgical__biopsy__upper__lobe__rep140	1	0	1	2
Early__IPF__surgical__biopsy__upper__lobe__rep142	0	0	1	1
Early__IPF__surgical__biopsy__upper__lobe__rep144	0	0	1	1
Early__IPF__surgical__biopsy__upper__lobe__rep145	0	0	1	1
Advanced__IPF__explant__lower__lobe__rep146	0	0	1	1
Early__IPF__surgical__biopsy__lower__lobe__rep149	0	0	1	1
Healthy__donor__biological__replicate2	1	0	0	1
Healthy__donor__biological__replicate4	1	1	0	2
Healthy__donor__biological__replicate5	1	1	0	2
Healthy__donor__biological__replicate6	1	1	0	2
Advanced__IPF__explant__lower__lobe__rep152	1	1	0	2
Advanced__IPF__explant__lower__lobe__rep158	1	0	0	1

1.1 Outliers (All Methods)

Outliers overall
Early__IPF__surgical__biopsy__upper__lobe__rep140
Healthy__donor__biological__replicate4
Healthy__donor__biological__replicate5
Healthy__donor__biological__replicate6
Advanced__IPF__explant__lower__lobe__rep152

1.2 Outliers (At Least One Method)

Outliers at least 1
Early_IPF_surgical_biopsy_upper_lobe_rep140
Early_IPF_surgical_biopsy_upper_lobe_rep142
Early_IPF_surgical_biopsy_upper_lobe_rep144
Early_IPF_surgical_biopsy_upper_lobe_rep145
Advanced_IPF_explant_lower_lobe_rep146
Early_IPF_surgical_biopsy_lower_lobe_rep149
Healthy_donor_biological_replicate2
Healthy_donor_biological_replicate4
Healthy_donor_biological_replicate5
Healthy_donor_biological_replicate6
Advanced_IPF_explant_lower_lobe_rep152
Advanced_IPF_explant_lower_lobe_rep158

2 RNA Degradation

2.1 Summarized Mean QC

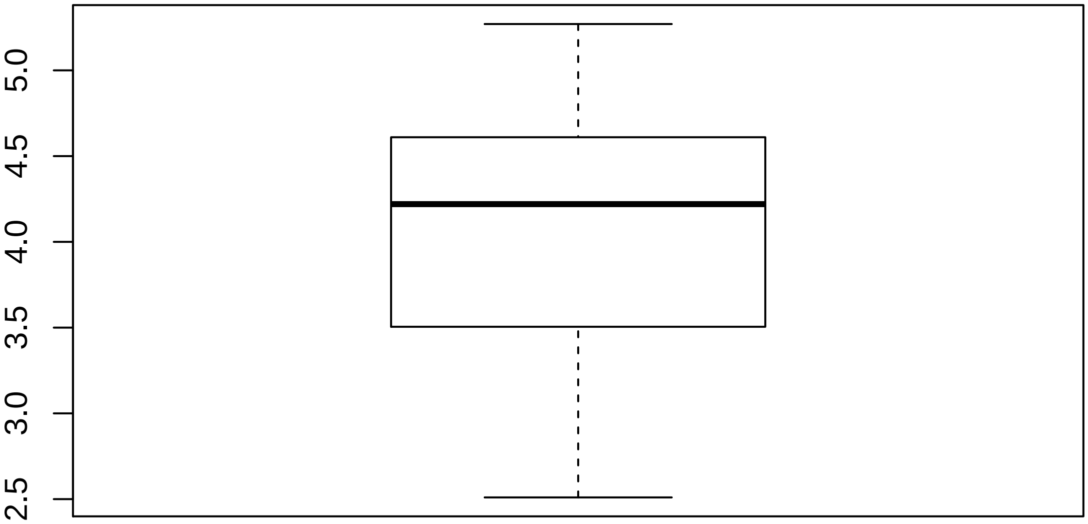

2.2 Discrete QC Plots

Sample Group [1]

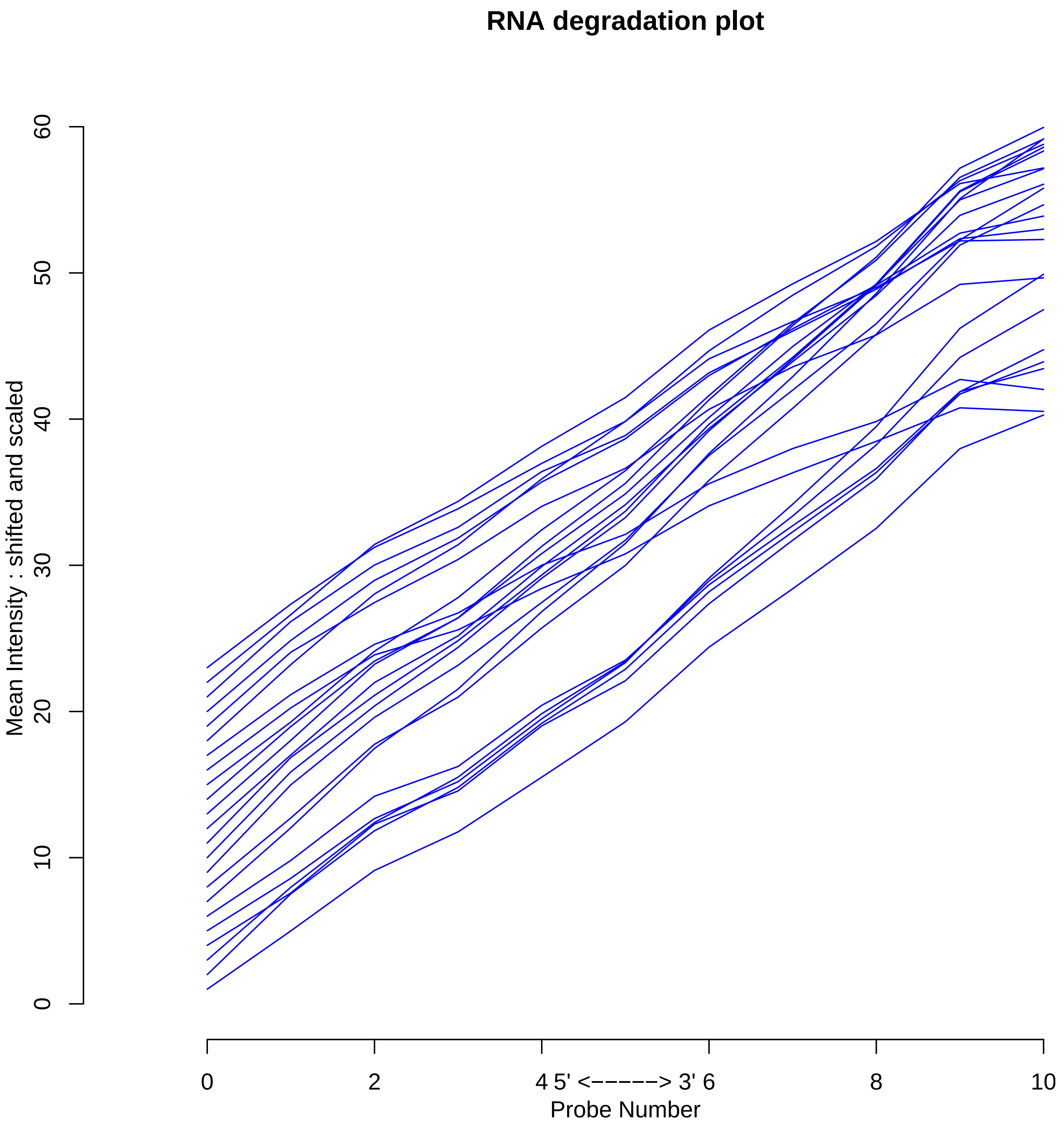

3 Relative Log Expression

3.1 Summarized Median QC

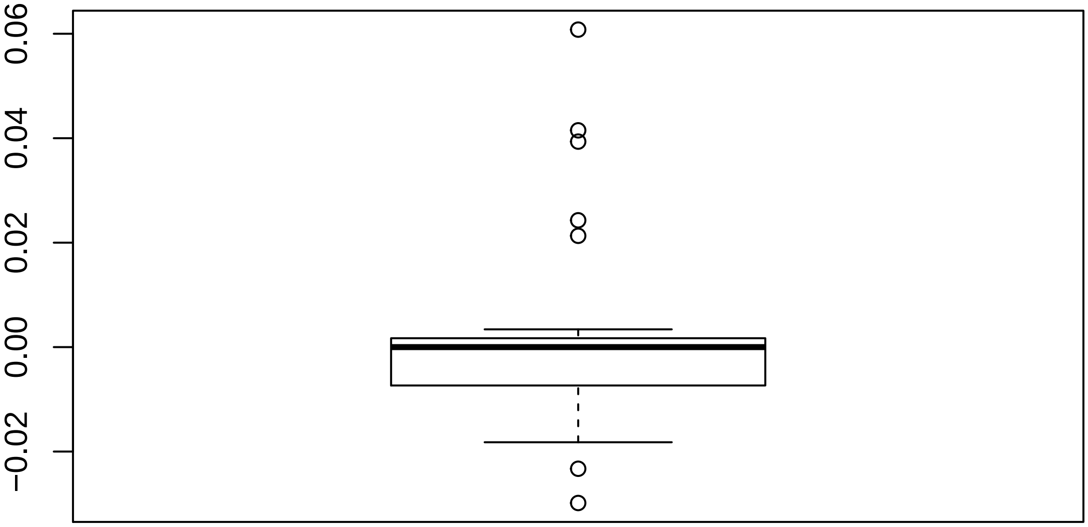

3.2 Discrete QC Plots

Sample Group [1]

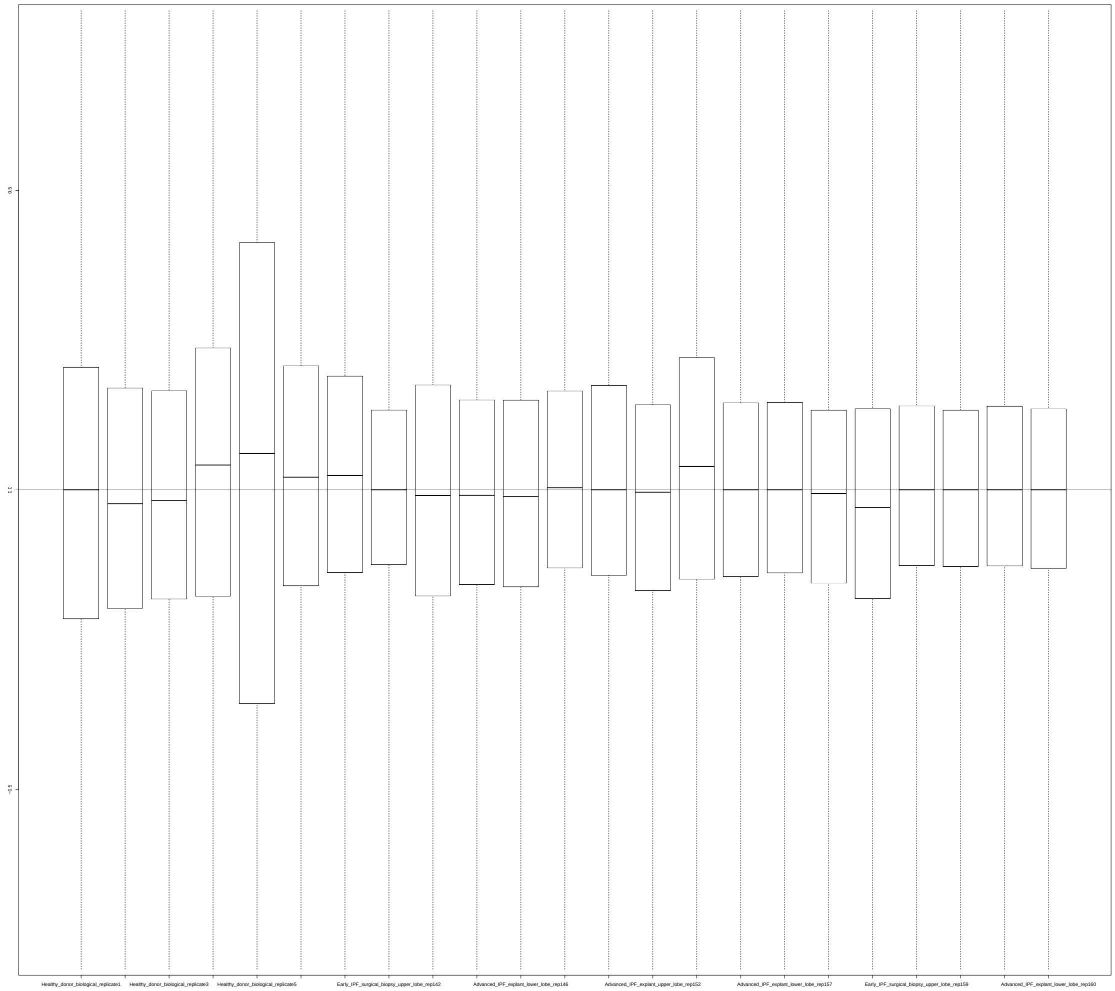

4 Normalized Unscaled Standard Errors

4.1 Summarized Median QC

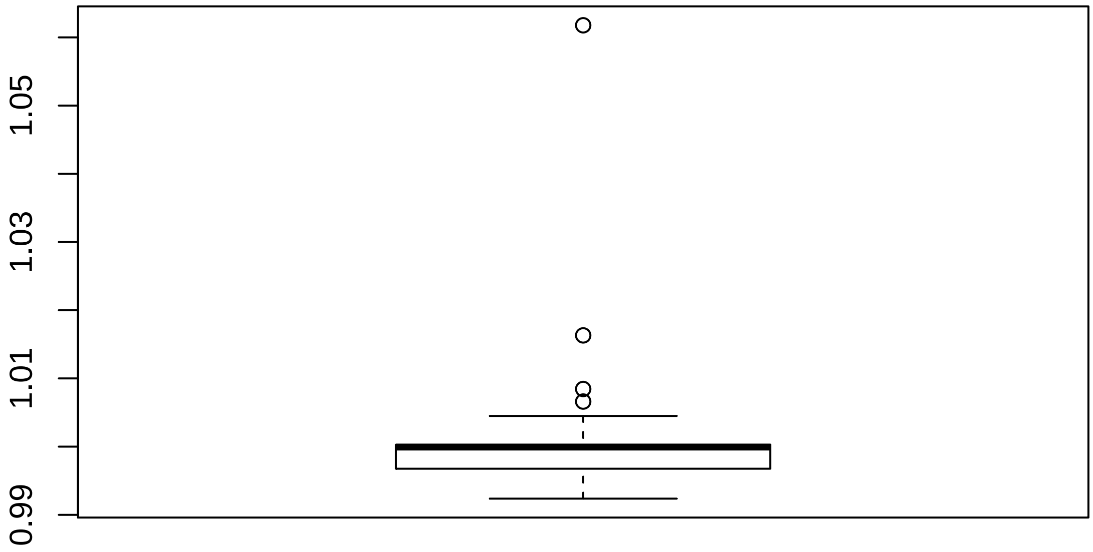

4.2 Discrete QC Plots

Sample Group [1]

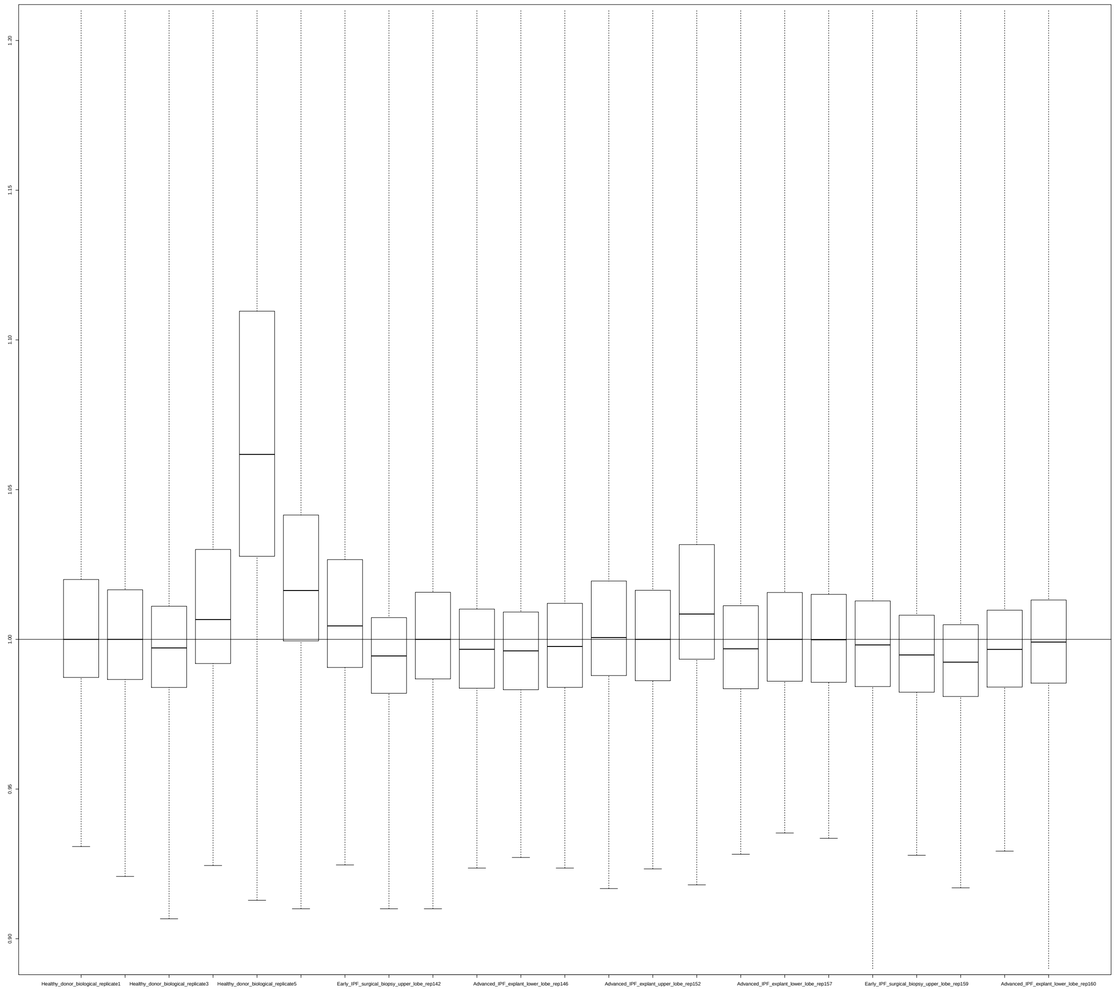

5 YAQC Plots

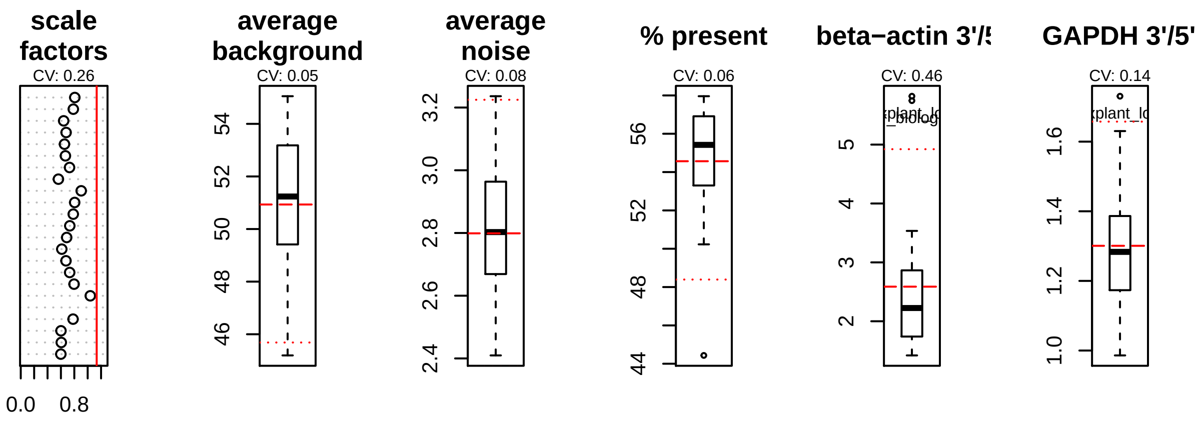
scale factors
CV: 0.26
0.0
0.8
average background
CV: 0.05
average noise
CV: 0.08
% present
CV: 0.06
beta−actin 3'/5'
CV: 0.46
GAPDH 3'/5'
CV: 0.14

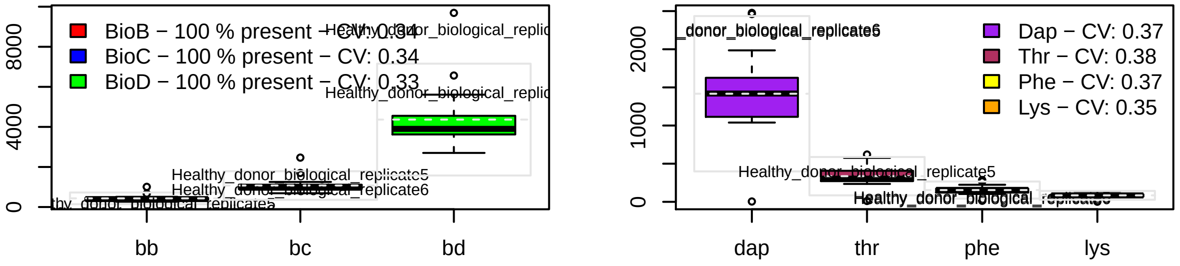
BioB − 100 % present − CV: 0.34
BioC − 100 % present − CV: 0.34
BioD − 100 % present − CV: 0.33
bb
bc
bd
Dap − CV: 0.37
Thr − CV: 0.38
Phe − CV: 0.37
Lys − CV: 0.35
dap
thr
phe
lys
