# Supplementary material for: Curated and harmonised transcriptomics datasets of interstitial lung diseases
Source: Data Brief. 2025 Oct 14;63:112139. doi: 10.1016/j.dib.2025.112139 (PMC12581653; doi:10.1016/j.dib.2025.112139)

eUTOPIA Affymetrix QC Report

eUTOPIA

Contents

1 Outliers Table **1**

1.1 Outliers (All Methods) 2

1.2 Outliers (At Least One Method) 2

2 RNA Degradation **3**

2.1 Summarized Mean QC 3

2.2 Discrete QC Plots 4

3 Relative Log Expression **5**

3.1 Summarized Median QC 5

3.2 Discrete QC Plots 6

1 Outliers Table

	RLE	NUSE	DEG	SUM
GSM2978758	0	NA	1	1
GSM2978759	0	NA	1	1
GSM2978761	0	NA	1	1
GSM2978762	0	NA	1	1
GSM2978763	0	NA	1	1
GSM2978764	0	NA	1	1
GSM2978770	0	NA	1	1
GSM2978772	0	NA	1	1
GSM2978779	0	NA	1	1
GSM2978780	0	NA	1	1
GSM2978786	0	NA	1	1
GSM2978787	0	NA	1	1
GSM2978790	1	NA	0	1
GSM2978791	1	NA	0	1
GSM2978795	1	NA	0	1
GSM2978799	1	NA	0	1

1.1 Outliers (All Methods)

Outliers overall
NA

1.2 Outliers (At Least One Method)

Outliers at least 1
GSM2978758
GSM2978759
GSM2978761
GSM2978762
GSM2978763
GSM2978764
GSM2978770
GSM2978772
GSM2978779
GSM2978780
GSM2978786
GSM2978787
GSM2978790
GSM2978791
GSM2978795
GSM2978799

2 RNA Degradation

2.1 Summarized Mean QC

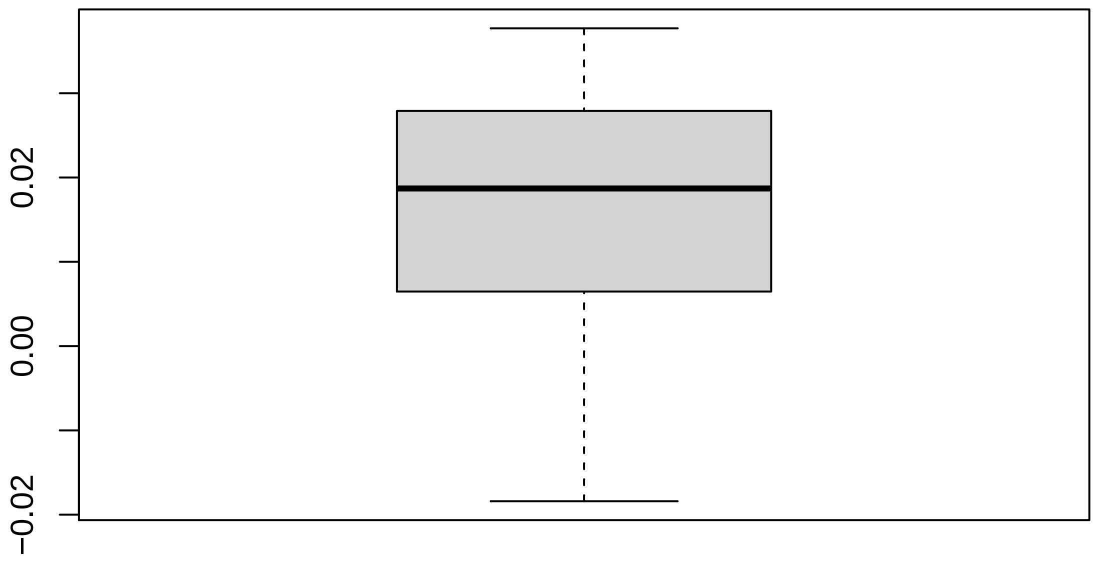

2.2 Discrete QC Plots

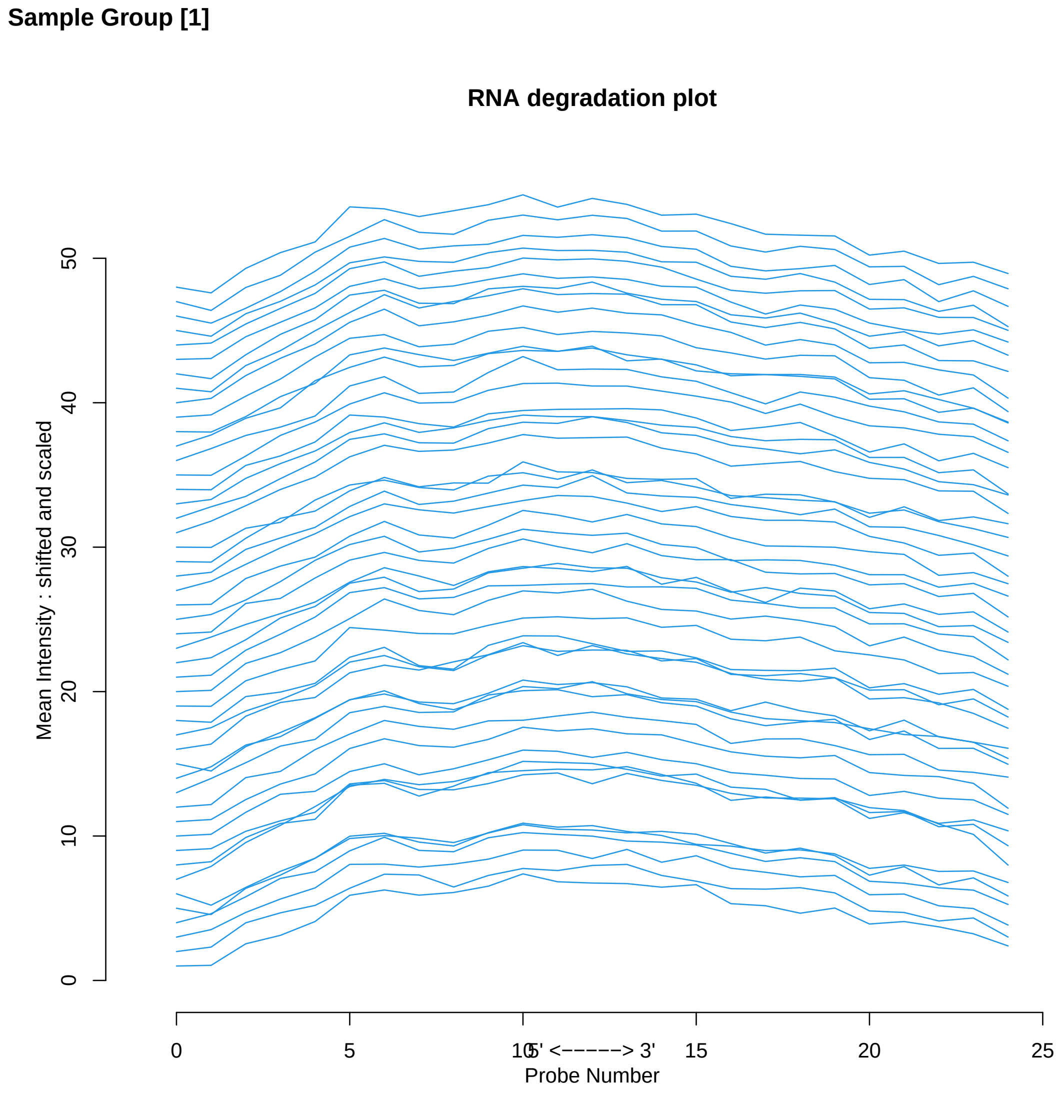

3 Relative Log Expression

3.1 Summarized Median QC

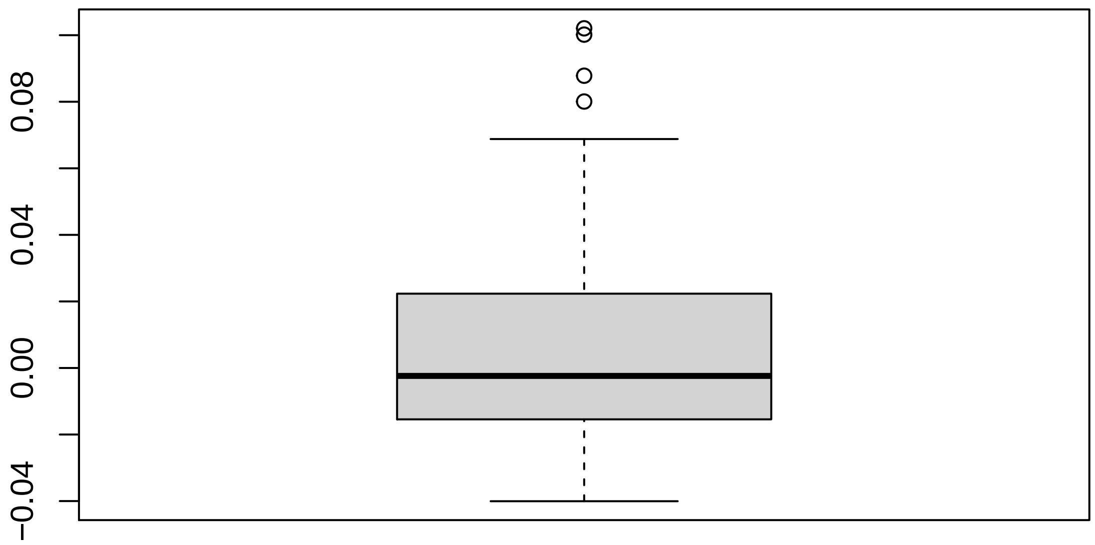

3.2 Discrete QC Plots

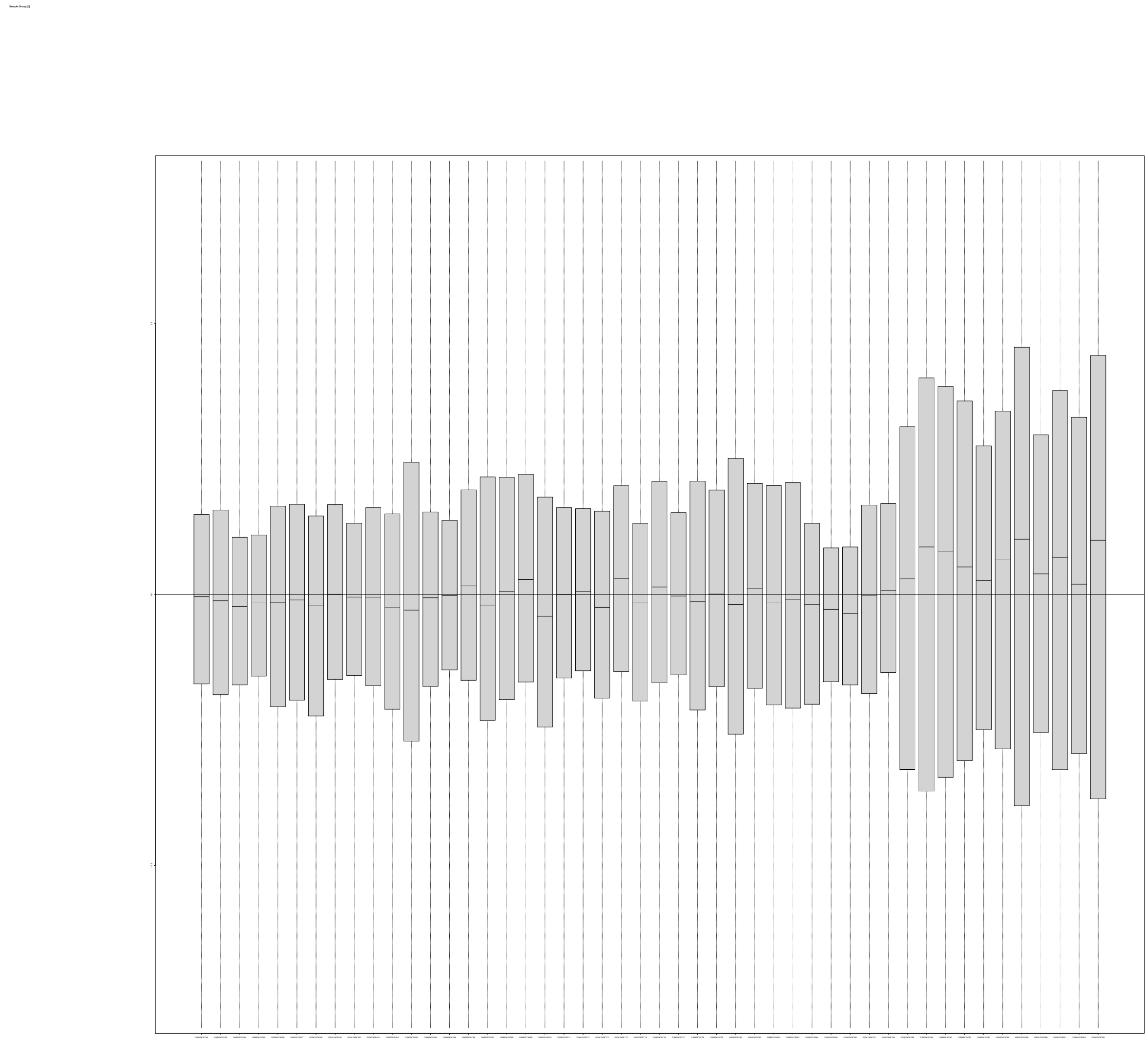

Supplement: Supplementary file 1 [file mmc1.zip › Supplementary_material/DNA-microarray/GSE110147/GSE110147_eUTOPIA_Affymetrix_QC_Report_2024-02-21.pdf]
