# Supplementary material for: Curated and harmonised transcriptomics datasets of interstitial lung diseases
Source: Data Brief. 2025 Oct 14;63:112139. doi: 10.1016/j.dib.2025.112139 (PMC12581653; doi:10.1016/j.dib.2025.112139)
Supplement: Supplementary file 1 [file mmc1.zip › Supplementary_material/DNA-microarray/GSE40839/GSE40839_eUTOPIA_Affymetrix_QC_Report_2024-02-12.pdf]

# eUTOPIA Affymetrix QC Report

*eUTOPIA*

## Contents


**1 Outliers Table** **1**
- 1.1 Outliers (All Methods) . . . . . 1
- 1.2 Outliers (At Least One Method) . . . . . 2

**2 RNA Degradation** **2**
- 2.1 Summarized Mean QC . . . . . 2
- 2.2 Discrete QC Plots . . . . . 3

**3 Relative Log Expression** **4**
- 3.1 Summarized Median QC . . . . . 4
- 3.2 Discrete QC Plots . . . . . 5

**4 Normalized Unscaled Standard Errors** **6**
- 4.1 Summarized Median QC . . . . . 6
- 4.2 Discrete QC Plots . . . . . 7

**5 YAQC Plots** **8**


# 1 Outliers Table

| | RLE | NUSE | DEG | SU |
|---|---|---|---|---|
| Pulmonary_fibroblasts_control_individual___biological_rep4 | 1 | 0 | 1 | [illegible] |
| Pulmonary_fibroblasts_control_individual___biological_rep6 | 1 | 0 | 1 | [illegible] |
| Pulmonary_fibroblasts_scleroderma_associated_interstitial_lung_disease___biological_rep7 | 0 | 0 | 1 | [illegible] |
| Pulmonary_fibroblasts_scleroderma_associated_interstitial_lung_disease___biological_rep8 | 0 | 0 | 1 | [illegible] |
| Pulmonary_fibroblasts_Usual_Interstitial_Pneumonia___biological_rep1 | 1 | 0 | 1 | [illegible] |
| Pulmonary_fibroblasts_control_individual___biological_rep5 | 1 | 0 | 0 | [illegible] |
| Pulmonary_fibroblasts_control_individual___biological_rep7 | 1 | 1 | 0 | [illegible] |
| Pulmonary_fibroblasts_control_individual___biological_rep8 | 1 | 0 | 0 | [illegible] |
| Pulmonary_fibroblasts_scleroderma_associated_interstitial_lung_disease___biological_rep1 | 1 | 0 | 0 | [illegible] |
| Pulmonary_fibroblasts_scleroderma_associated_interstitial_lung_disease___biological_rep2 | 1 | 0 | 0 | [illegible] |
| Pulmonary_fibroblasts_scleroderma_associated_interstitial_lung_disease___biological_rep4 | 1 | 0 | 0 | [illegible] |
| Pulmonary_fibroblasts_Usual_Interstitial_Pneumonia___biological_rep2 | 1 | 0 | 0 | [illegible] |

## 1.1 Outliers (All Methods)

| Outliers overall |
|---|
| Pulmonary_fibroblasts_control_individual___biological_rep4 |
| Pulmonary_fibroblasts_control_individual___biological_rep6 |
| Pulmonary_fibroblasts_Usual_Interstitial_Pneumonia___biological_rep1 |
| Pulmonary_fibroblasts_control_individual___biological_rep7 |

### 1.2 Outliers (At Least One Method)

| Outliers at least 1 |
| --- |
| Pulmonary__fibroblasts__control__individual___biological__rep4 |
| Pulmonary__fibroblasts__control__individual___biological__rep6 |
| Pulmonary__fibroblasts__scleroderma__associated__interstitial__lung__disease___biological__rep7 |
| Pulmonary__fibroblasts__scleroderma__associated__interstitial__lung__disease___biological__rep8 |
| Pulmonary__fibroblasts__Usual__Interstitial__Pneumonia___biological__rep1 |
| Pulmonary__fibroblasts__control__individual___biological__rep5 |
| Pulmonary__fibroblasts__control__individual___biological__rep7 |
| Pulmonary__fibroblasts__control__individual___biological__rep8 |
| Pulmonary__fibroblasts__scleroderma__associated__interstitial__lung__disease___biological__rep1 |
| Pulmonary__fibroblasts__scleroderma__associated__interstitial__lung__disease___biological__rep2 |
| Pulmonary__fibroblasts__scleroderma__associated__interstitial__lung__disease___biological__rep4 |
| Pulmonary__fibroblasts__Usual__Interstitial__Pneumonia___biological__rep2 |

## 2 RNA Degradation

### 2.1 Summarized Mean QC

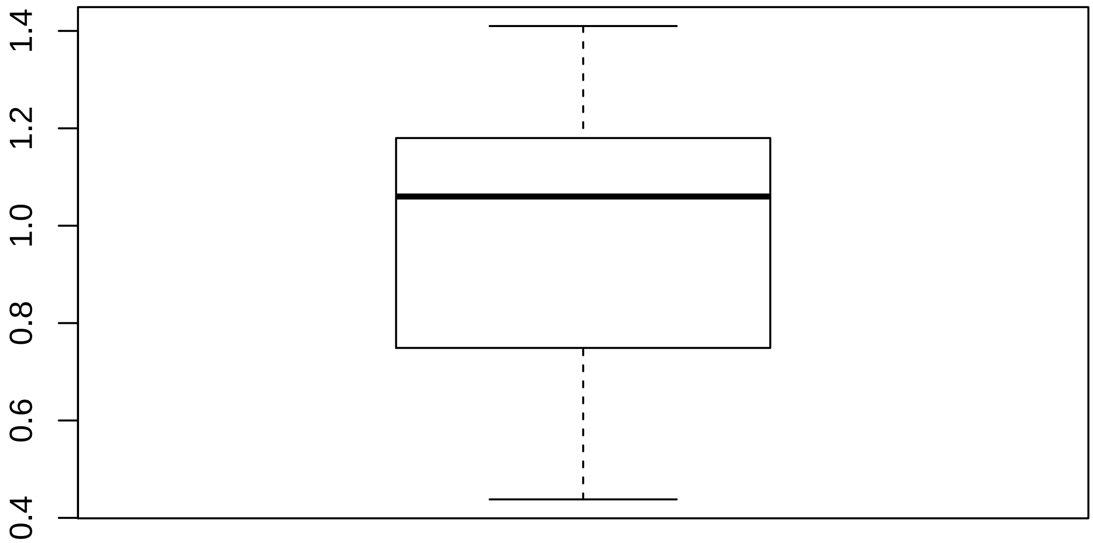

## 2.2 Discrete QC Plots

**Sample Group [1]**

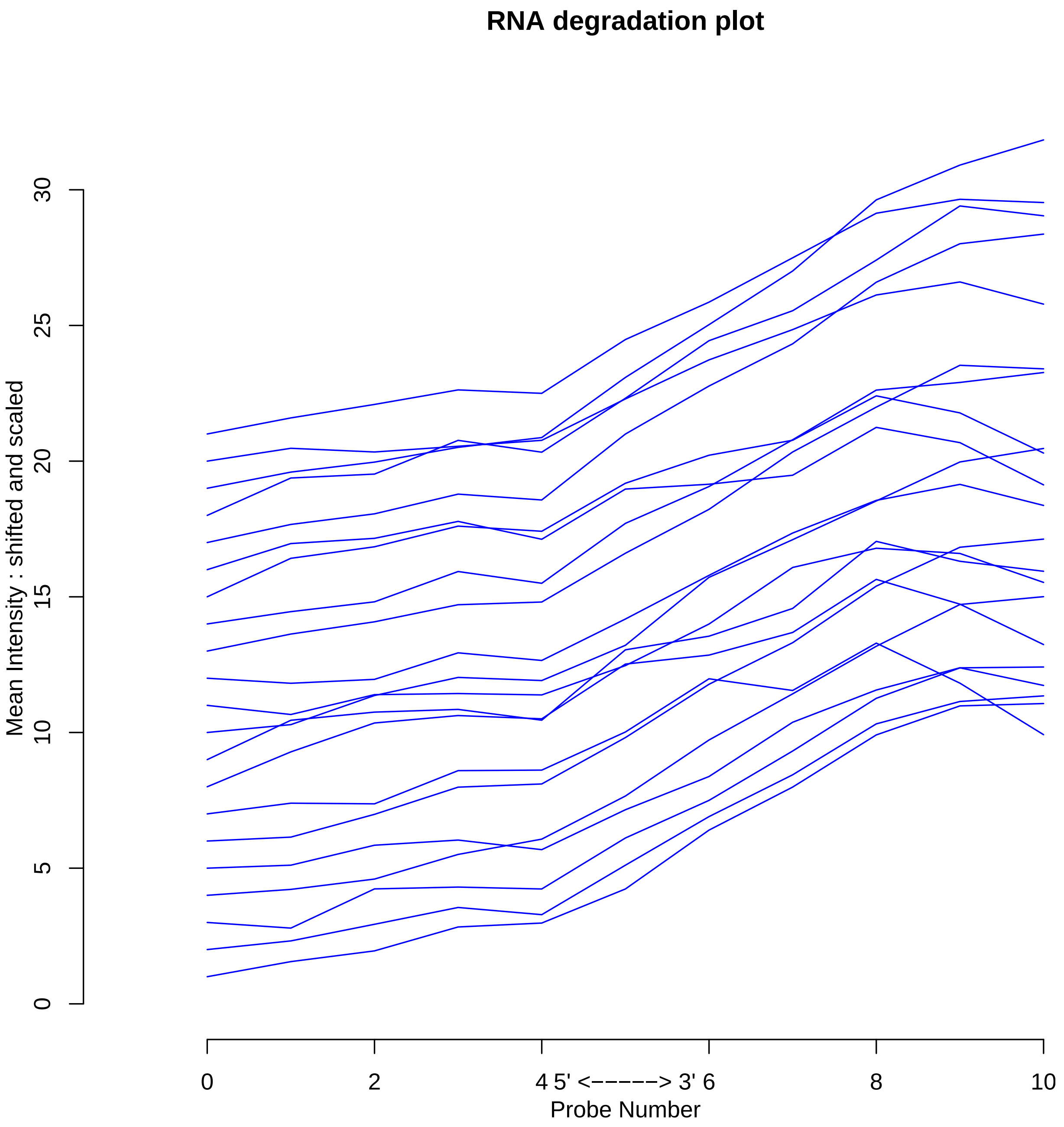

# 3 Relative Log Expression

## 3.1 Summarized Median QC

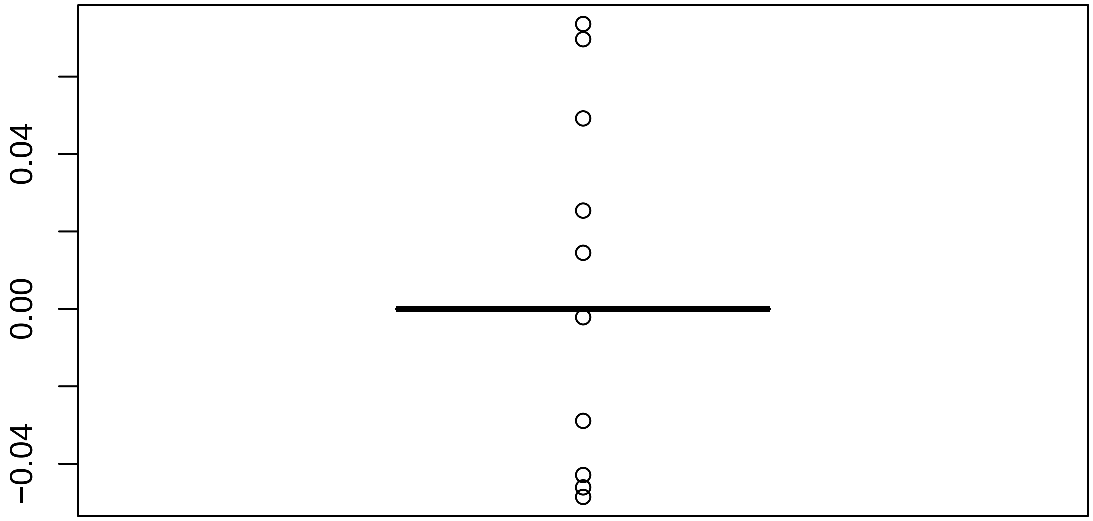

## 3.2 Discrete QC Plots

Sample Group [1]

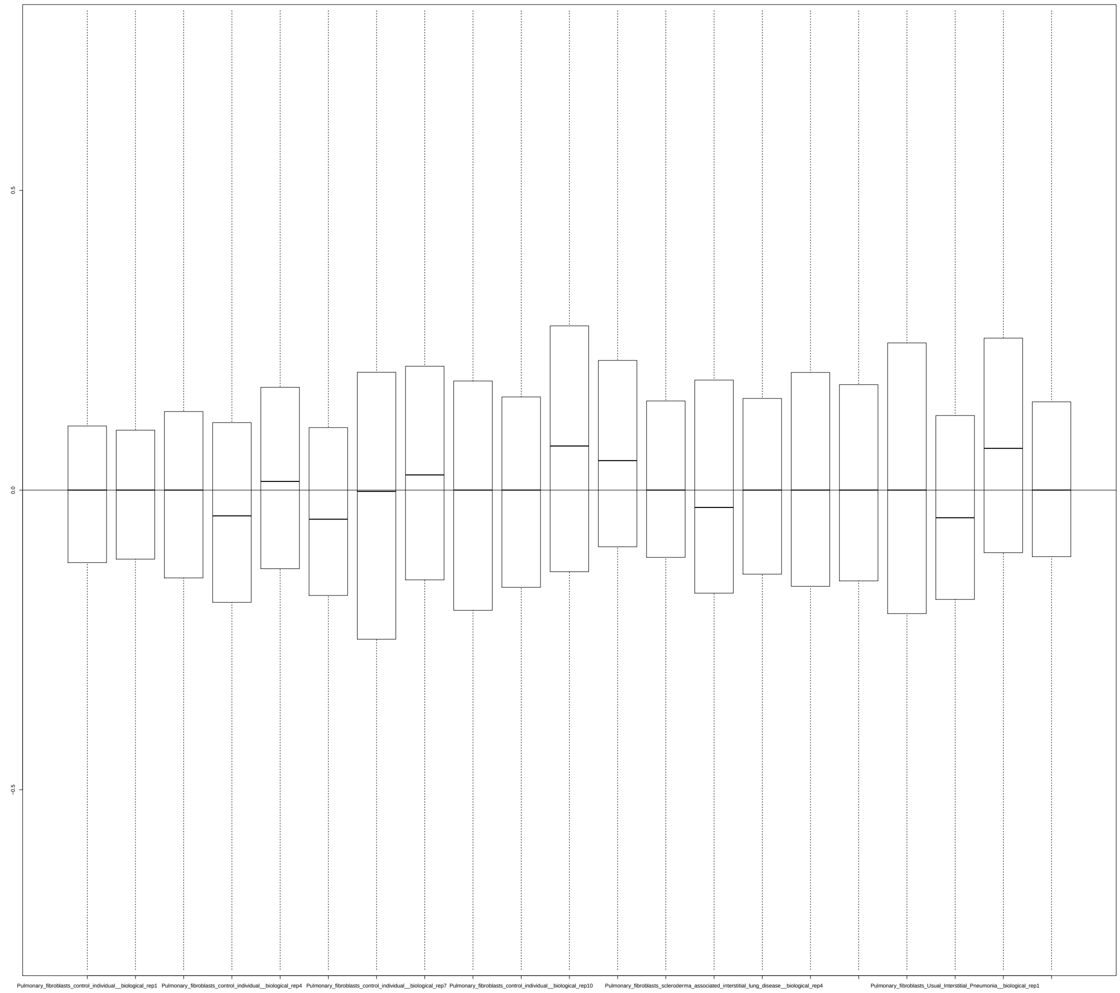

# 4 Normalized Unscaled Standard Errors

## 4.1 Summarized Median QC

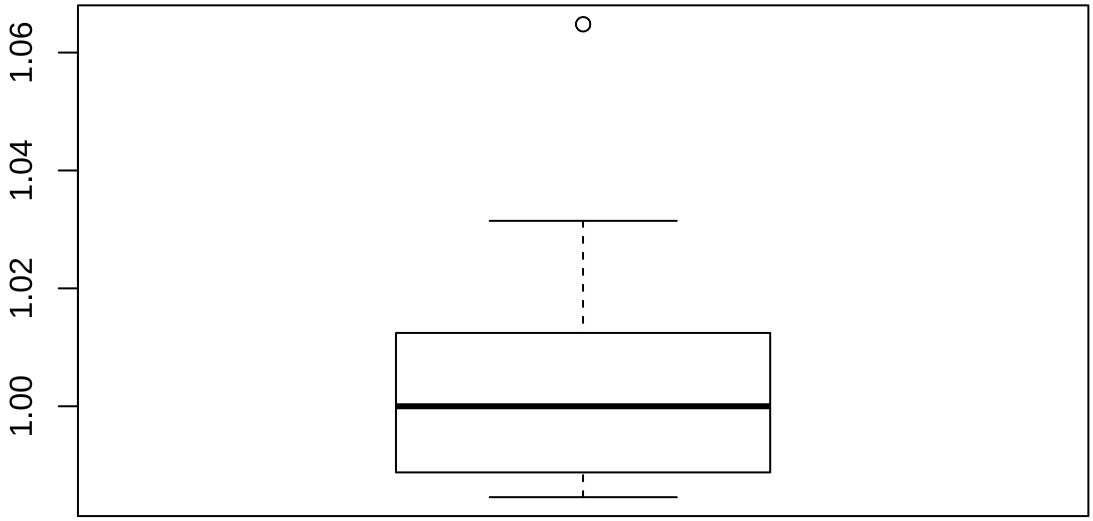

## 4.2 Discrete QC Plots

Sample Group [1]

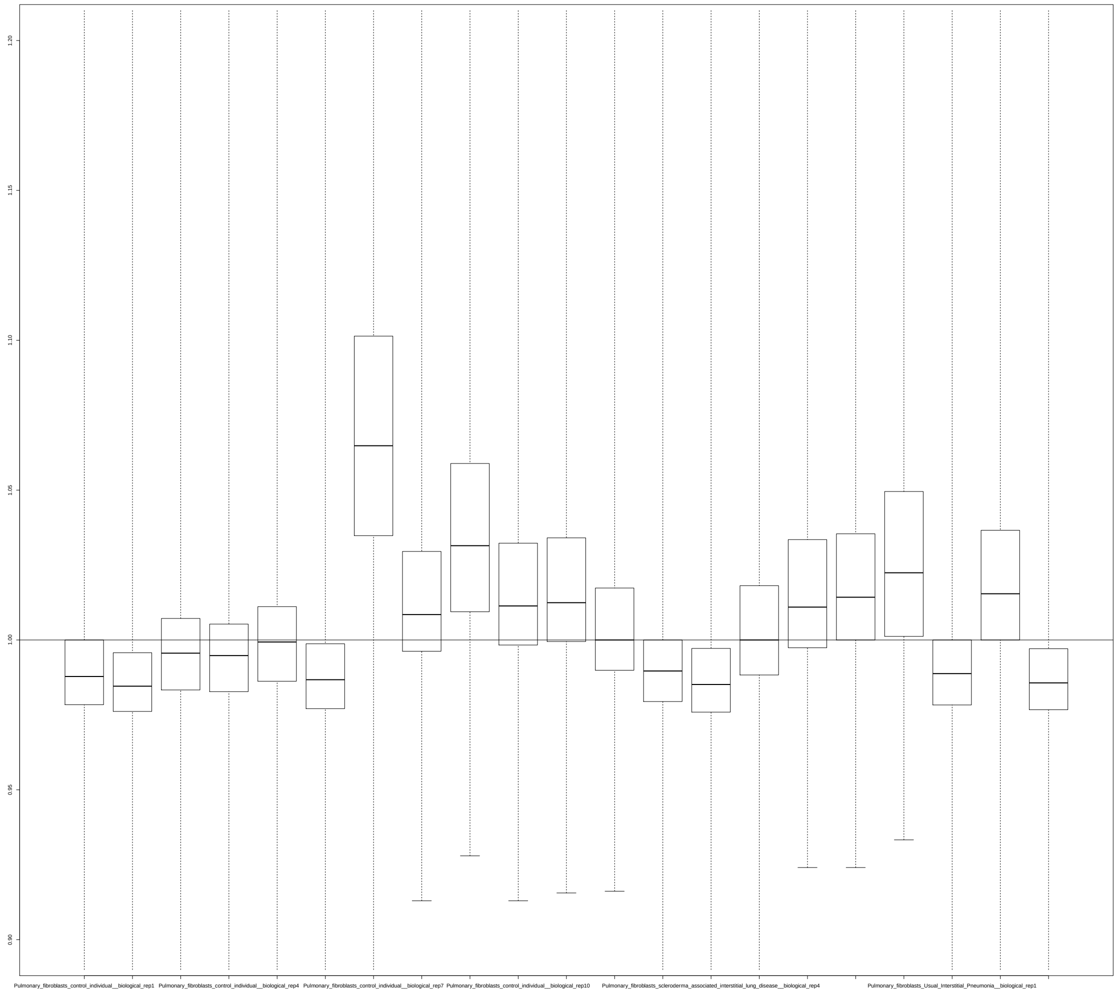

## 5 YAQC Plots

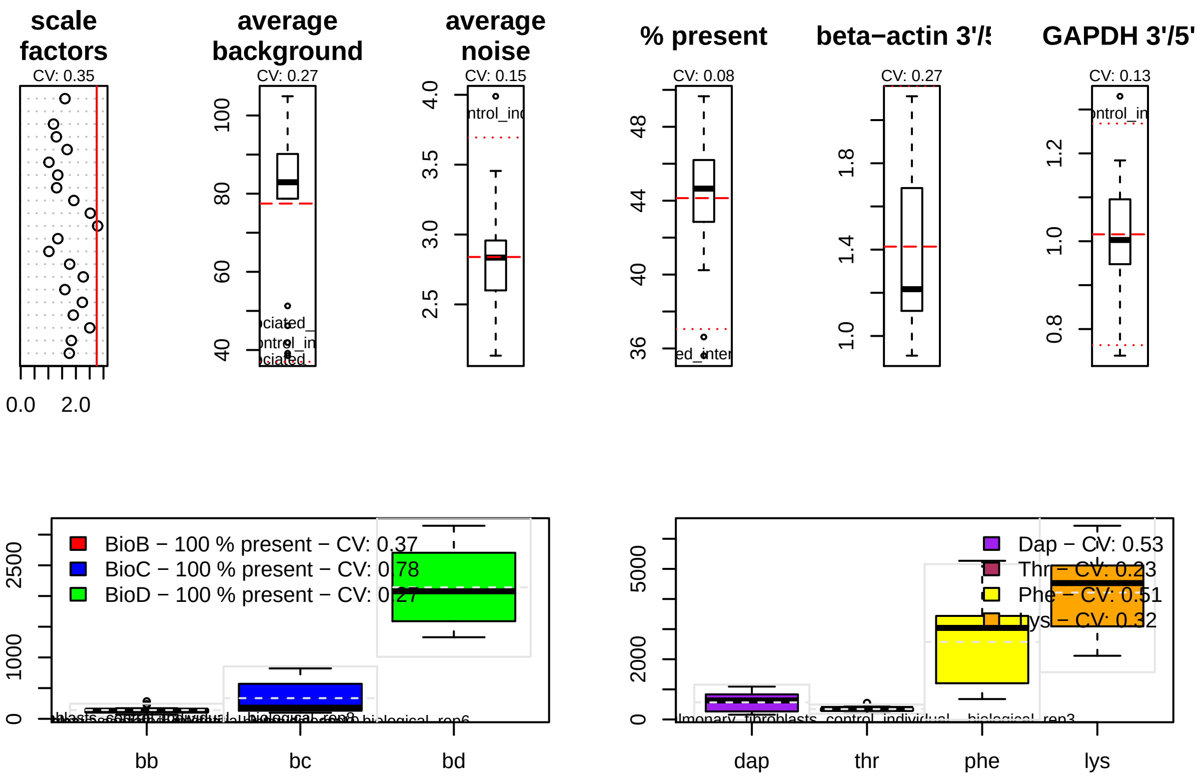
scale factors
CV: 0.35
0.0
2.0
average background
CV: 0.27
average noise
CV: 0.15
% present
CV: 0.08
beta−actin 3'/5
CV: 0.27
GAPDH 3'/5'
CV: 0.13
BioB − 100 % present − CV: 0.37
BioC − 100 % present − CV: 0.78
BioD − 100 % present − CV: 0.27
bb
bc
bd
Dap − CV: 0.53
Thr − CV: 0.23
Phe − CV: 0.51
lys − CV: 0.32
dap
thr
phe
lys
