# Supplementary material for: Curated and harmonised transcriptomics datasets of interstitial lung diseases
Source: Data Brief. 2025 Oct 14;63:112139. doi: 10.1016/j.dib.2025.112139 (PMC12581653; doi:10.1016/j.dib.2025.112139)

eUTOPIA Affymetrix QC Report

eUTOPIA

Contents

1 Outliers Table **1**
1.1 Outliers (All Methods) 1
1.2 Outliers (At Least One Method) 1

2 RNA Degradation **2**
2.1 Summarized Mean QC 2
2.2 Discrete QC Plots 3

3 Relative Log Expression **4**
3.1 Summarized Median QC 4
3.2 Discrete QC Plots 5

4 Normalized Unscaled Standard Errors **6**
4.1 Summarized Median QC 6
4.2 Discrete QC Plots 7

1 Outliers Table

	RLE	NUSE	DEG	SUM
Primary_ADC_cell_line_3	0	1	1	2
Primary_control_cell_line_3	0	0	1	1

1.1 Outliers (All Methods)

Outliers overall
Primary_ADC_cell_line_3

1.2 Outliers (At Least One Method)

Outliers at least 1
Primary_ADC_cell_line_3
Primary_control_cell_line_3

2 RNA Degradation

2.1 Summarized Mean QC

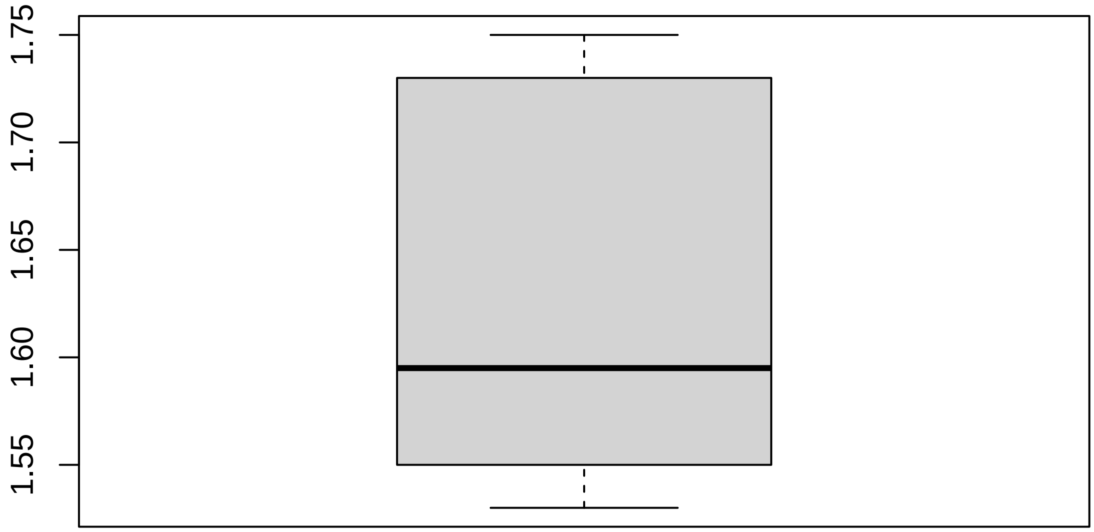

2.2 Discrete QC Plots

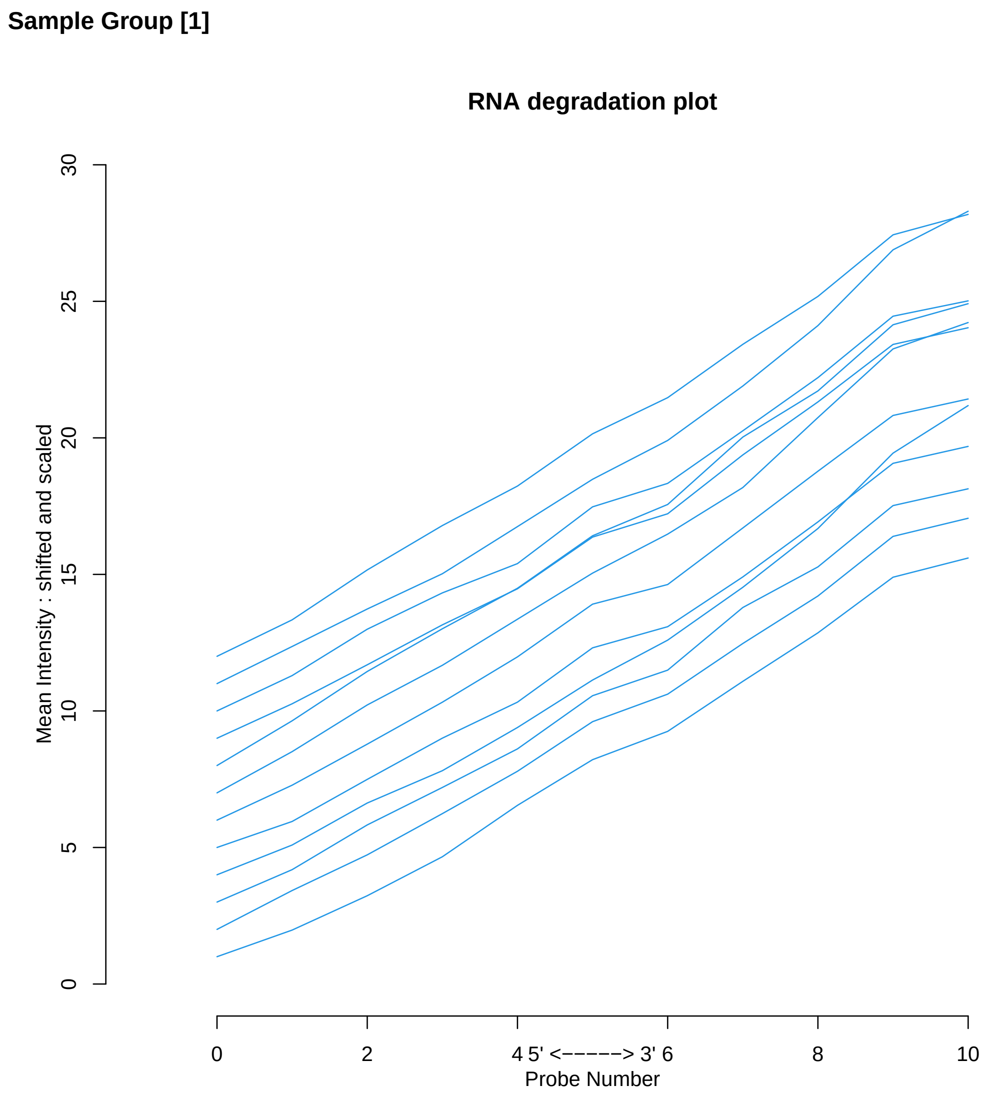

3 Relative Log Expression

3.1 Summarized Median QC

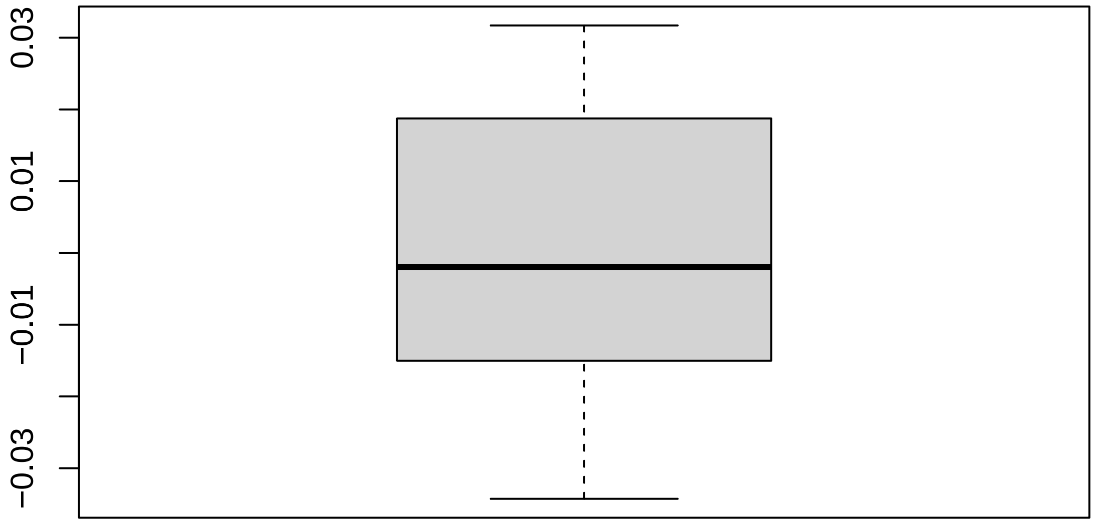

3.2 Discrete QC Plots

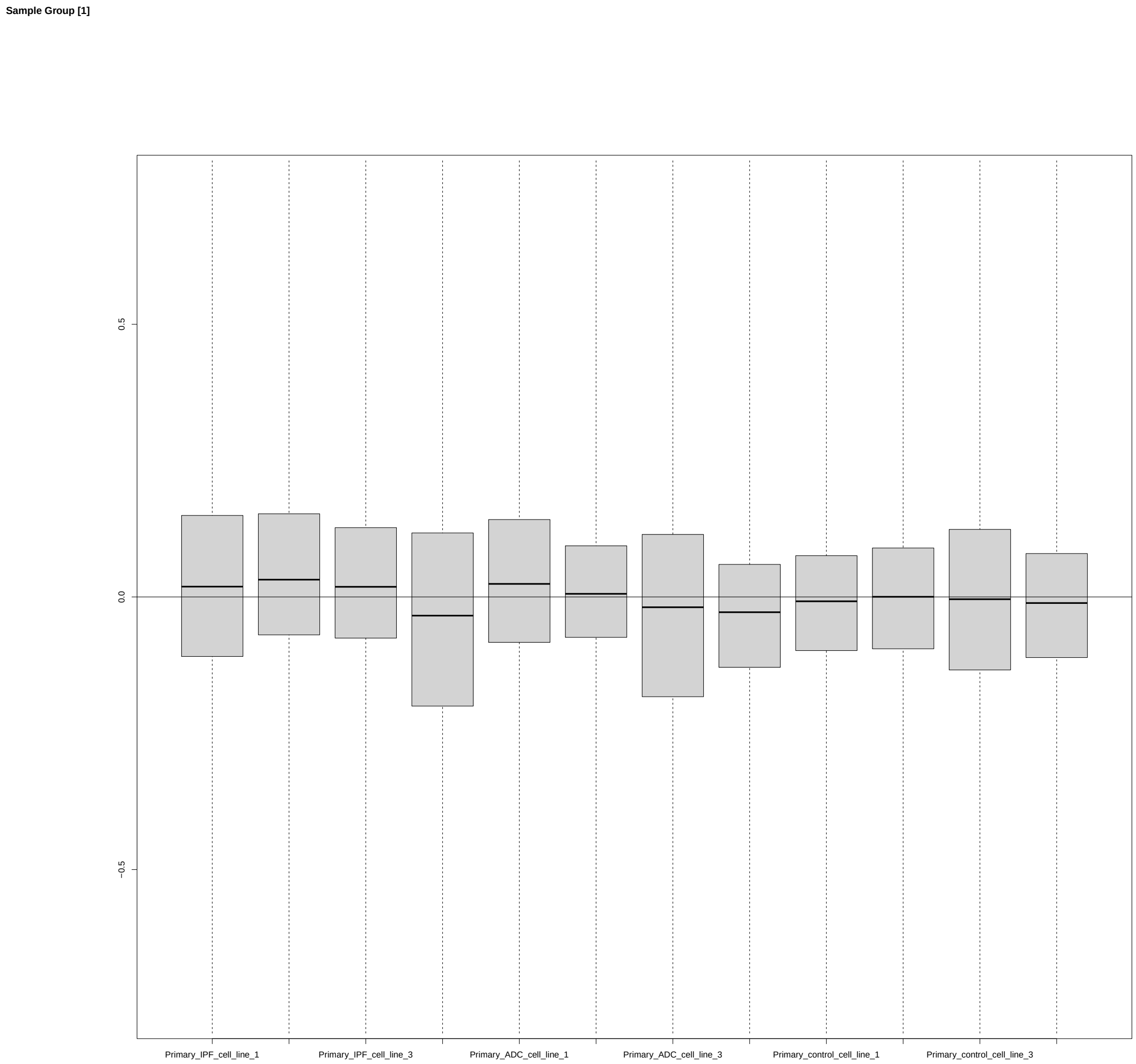

4 Normalized Unscaled Standard Errors

4.1 Summarized Median QC

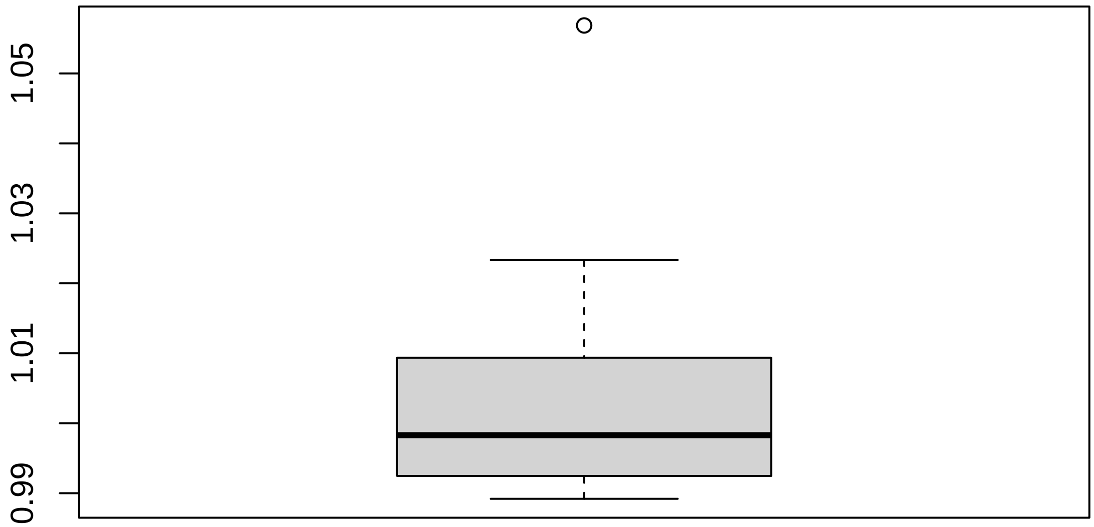

4.2 Discrete QC Plots

Sample Group [1]

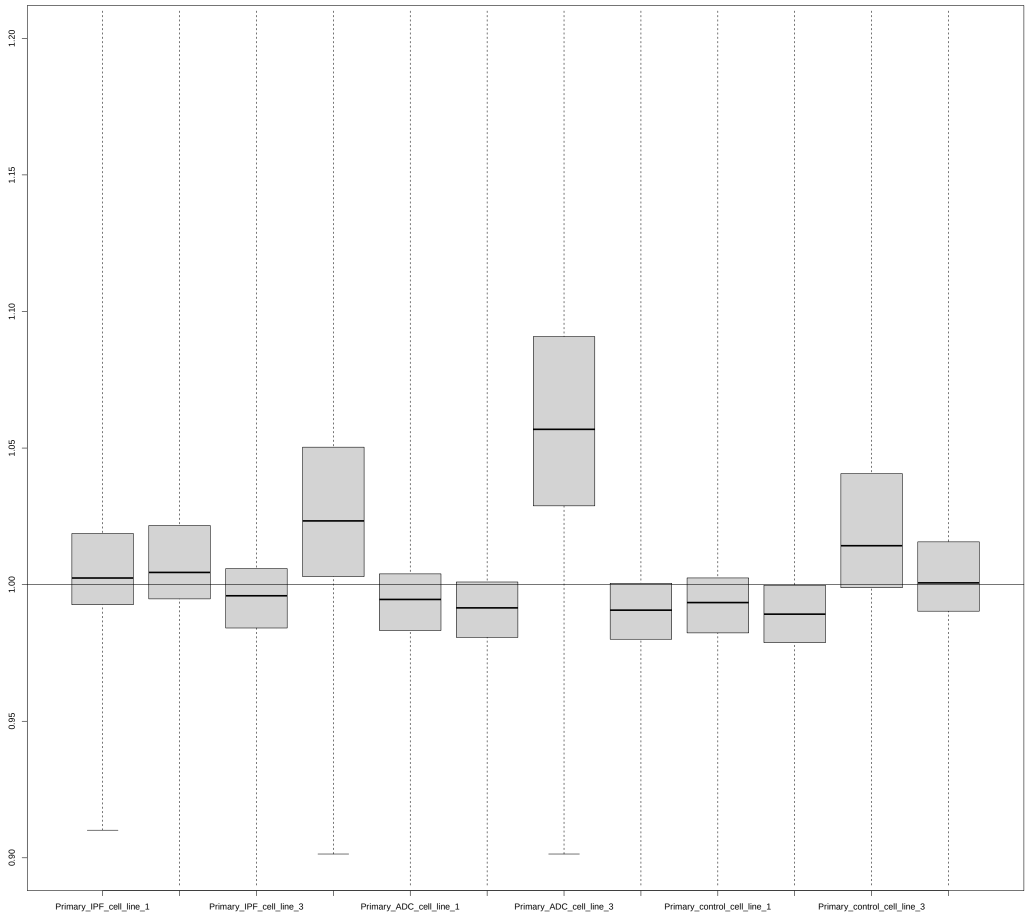

Supplement: Supplementary file 1 [file mmc1.zip › Supplementary_material/DNA-microarray/GSE144338/GSE144338_eUTOPIA_Affymetrix_QC_Report_2024-02-19.pdf]
