# Supplementary material for: Curated and harmonised transcriptomics datasets of interstitial lung diseases
Source: Data Brief. 2025 Oct 14;63:112139. doi: 10.1016/j.dib.2025.112139 (PMC12581653; doi:10.1016/j.dib.2025.112139)
Supplement: Supplementary file 1 [file mmc1.zip › Supplementary_material/DNA-microarray/GSE49072/GSE49072_eUTOPIA_Affymetrix_QC_Report_2024-02-12-4.pdf]

eUTOPIA Affymetrix QC Report

eUTOPIA

Contents

1 Outliers Table **1**
- 1.1 Outliers (All Methods) 2
- 1.2 Outliers (At Least One Method) 2

2 RNA Degradation **3**
- 2.1 Summarized Mean QC 3
- 2.2 Discrete QC Plots 4

3 Relative Log Expression **6**
- 3.1 Summarized Median QC 6
- 3.2 Discrete QC Plots 7

4 Normalized Unscaled Standard Errors **9**
- 4.1 Summarized Median QC 9
- 4.2 Discrete QC Plots 10

5 YAQC Plots **12**

1 Outliers Table

	RLE	NUSE	DEG	SUM
Normal_Volunteer,_Replicate_21	0	0	1	1
Familial_IPF,_Replicate_3	0	0	1	1
Normal_Volunteer,_Replicate_25	0	0	1	1
Spontaneous_IPF,_Replicate_11	1	0	1	2
Normal_Volunteer,_Replicate_42	1	0	1	2
Spontaneous_IPF,_Replicate_14	0	1	1	2
Normal_Volunteer,_Replicate_5	0	0	1	1
Spontaneous_IPF,_Replicate_5	0	0	1	1
Normal_Volunteer,_Replicate_28	0	0	1	1
Normal_Volunteer,_Replicate_39	0	0	1	1
Spontaneous_IPF,_Replicate_12	0	1	1	2
Familial_IPF,_Replicate_2	0	0	1	1
Spontaneous_IPF,_Replicate_9	0	0	1	1
Normal_Volunteer,_Replicate_41	0	0	1	1
Spontaneous_IPF,_Replicate_13	0	0	1	1
Normal_Volunteer,_Replicate_45	0	1	1	2
Familial_IPF,_Replicate_1	0	0	1	1
Normal_Volunteer,_Replicate_24	0	0	1	1
Spontaneous_IPF,_Replicate_6	0	0	1	1
Normal_Volunteer,_Replicate_40	0	0	1	1
Normal_Volunteer,_Replicate_43	0	0	1	1
Normal_Relative,_Replicate_9	1	1	0	2
Normal_Volunteer,_Replicate_18	1	0	0	1
Normal_Relative,_Replicate_1	1	0	0	1
Normal_Volunteer,_Replicate_12	1	0	0	1

	RLE	NUSE	DEG	SUM
Normal_Volunteer,_Replicate_15	1	0	0	1
Normal_Volunteer,_Replicate_32	1	1	0	2
Normal_Volunteer,_Replicate_35	0	1	0	1
Normal_Volunteer,_Replicate_23	0	1	0	1
Spontaneous_IPF,_Replicate_3	0	1	0	1
Familial_IPF,_Replicate_6	0	1	0	1
Spontaneous_IPF,_Replicate_15	0	1	0	1

1.1 Outliers (All Methods)

Outliers overall
Spontaneous_IPF,_Replicate_11
Normal_Volunteer,_Replicate_42
Spontaneous_IPF,_Replicate_14
Spontaneous_IPF,_Replicate_12
Normal_Volunteer,_Replicate_45
Normal_Relative,_Replicate_9
Normal_Volunteer,_Replicate_32

1.2 Outliers (At Least One Method)

Outliers at least 1
Normal_Volunteer,_Replicate_21
Familial_IPF,_Replicate_3
Normal_Volunteer,_Replicate_25
Spontaneous_IPF,_Replicate_11
Normal_Volunteer,_Replicate_42
Spontaneous_IPF,_Replicate_14
Normal_Volunteer,_Replicate_5
Spontaneous_IPF,_Replicate_5
Normal_Volunteer,_Replicate_28
Normal_Volunteer,_Replicate_39
Spontaneous_IPF,_Replicate_12
Familial_IPF,_Replicate_2
Spontaneous_IPF,_Replicate_9
Normal_Volunteer,_Replicate_41
Spontaneous_IPF,_Replicate_13
Normal_Volunteer,_Replicate_45
Familial_IPF,_Replicate_1
Normal_Volunteer,_Replicate_24
Spontaneous_IPF,_Replicate_6
Normal_Volunteer,_Replicate_40
Normal_Volunteer,_Replicate_43
Normal_Relative,_Replicate_9
Normal_Volunteer,_Replicate_18
Normal_Relative,_Replicate_1
Normal_Volunteer,_Replicate_12
Normal_Volunteer,_Replicate_15
Normal_Volunteer,_Replicate_32

Outliers at least 1
Normal_Volunteer,_Replicate_35
Normal_Volunteer,_Replicate_23
Spontaneous_IPF,_Replicate_3
Familial_IPF,_Replicate_6
Spontaneous_IPF,_Replicate_15

2 RNA Degradation

2.1 Summarized Mean QC

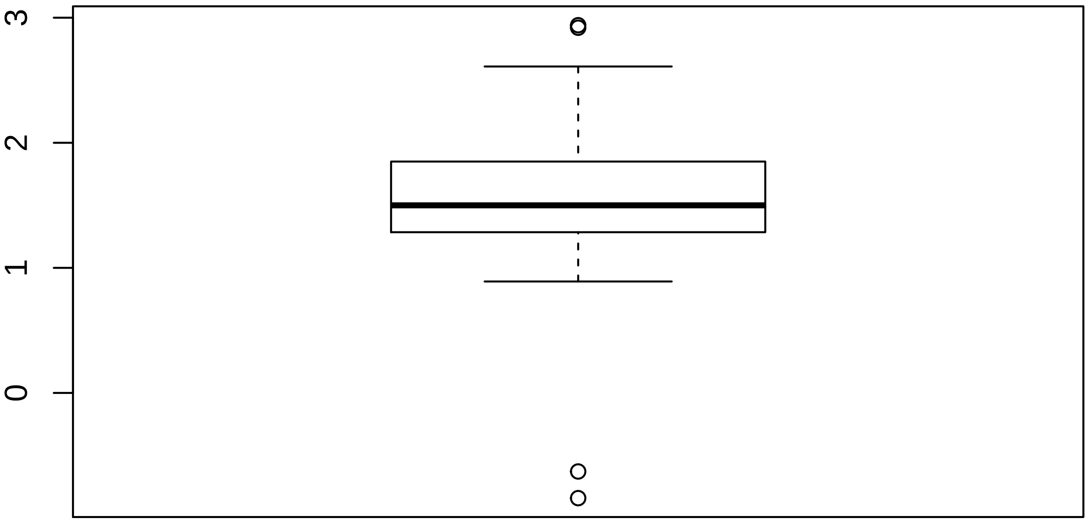

2.2 Discrete QC Plots

Sample Group [1]

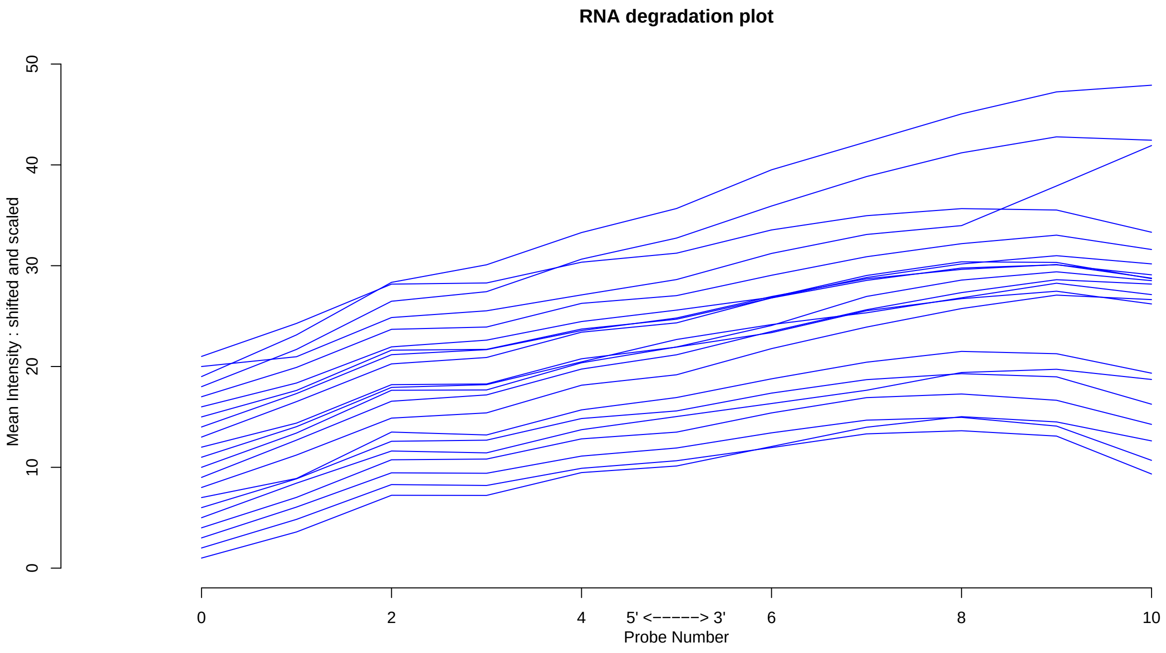

Sample Group [2]

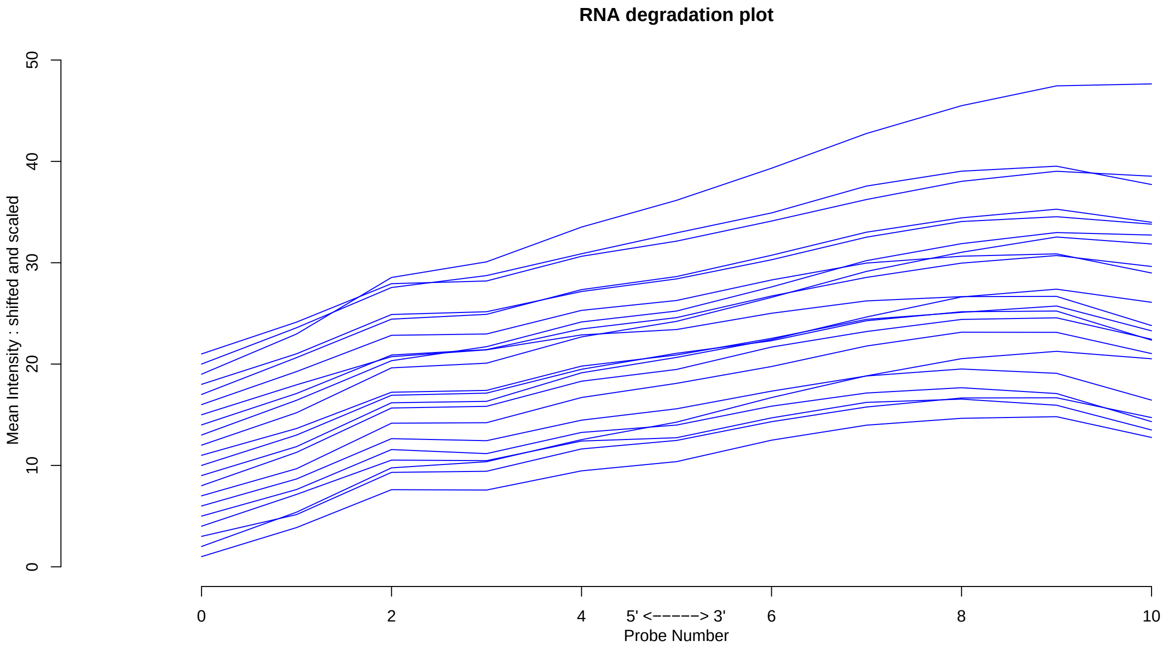

Sample Group [3]

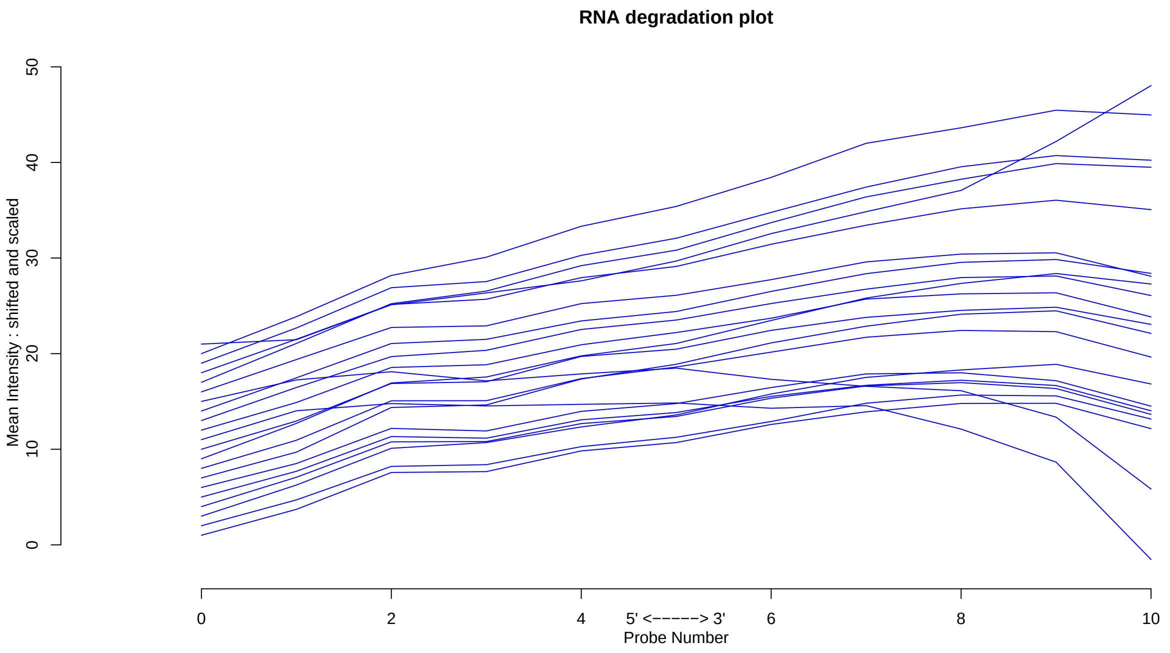

Sample Group [4]

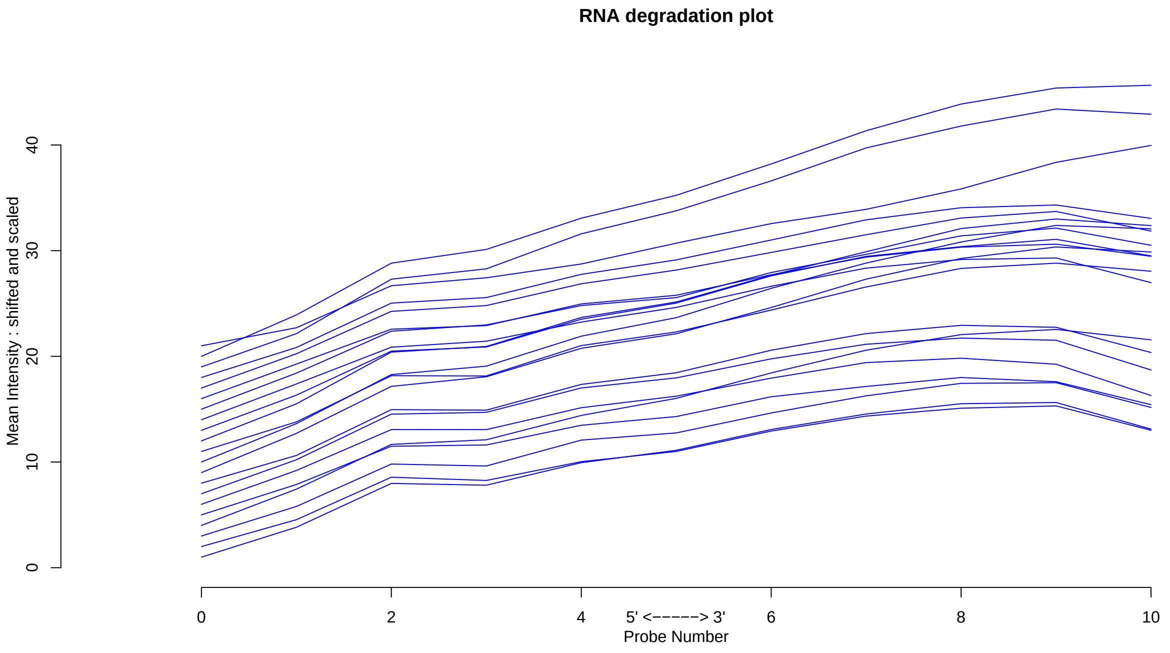

3 Relative Log Expression

3.1 Summarized Median QC

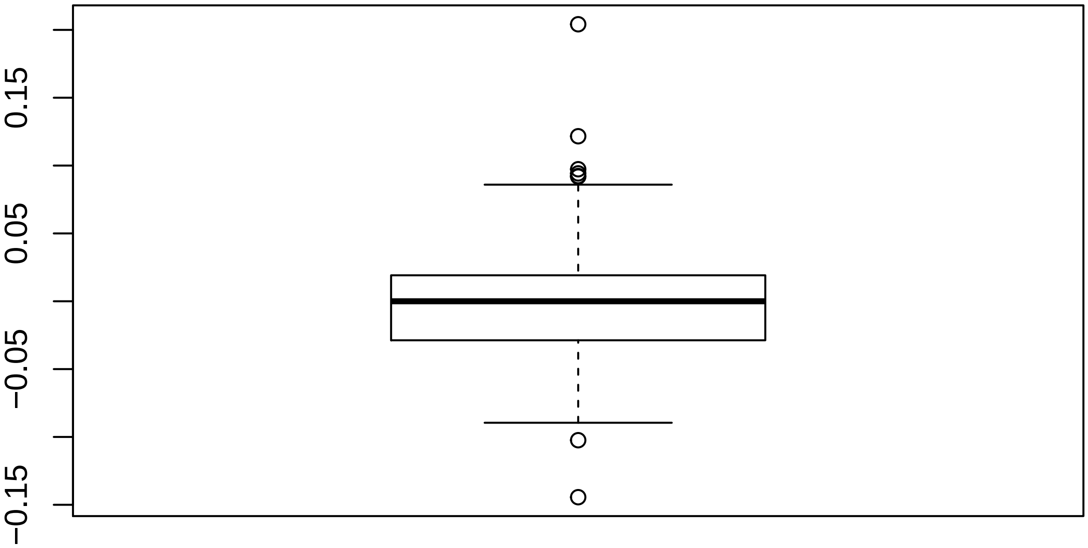

3.2 Discrete QC Plots

Sample Group [1]

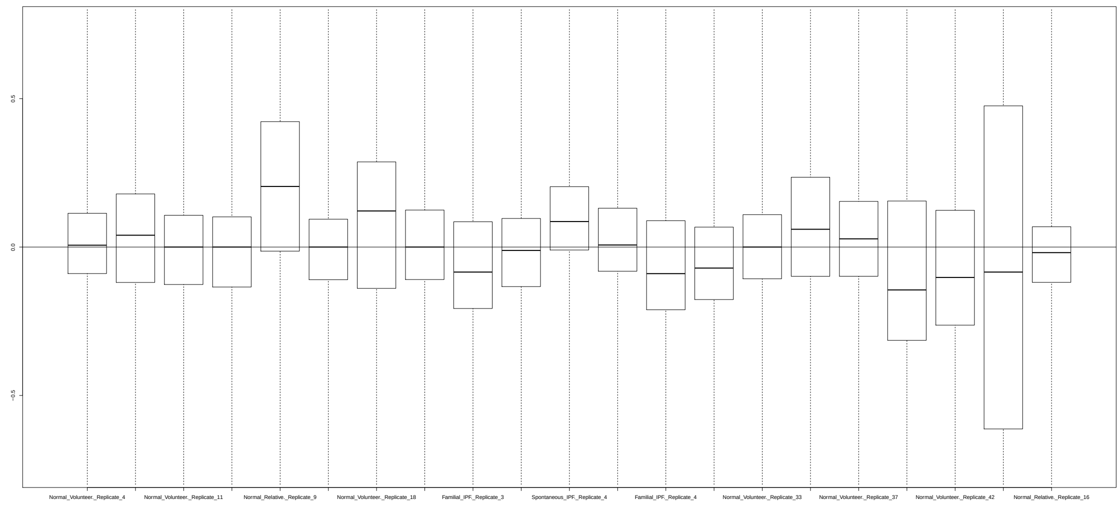

Sample Group [2]

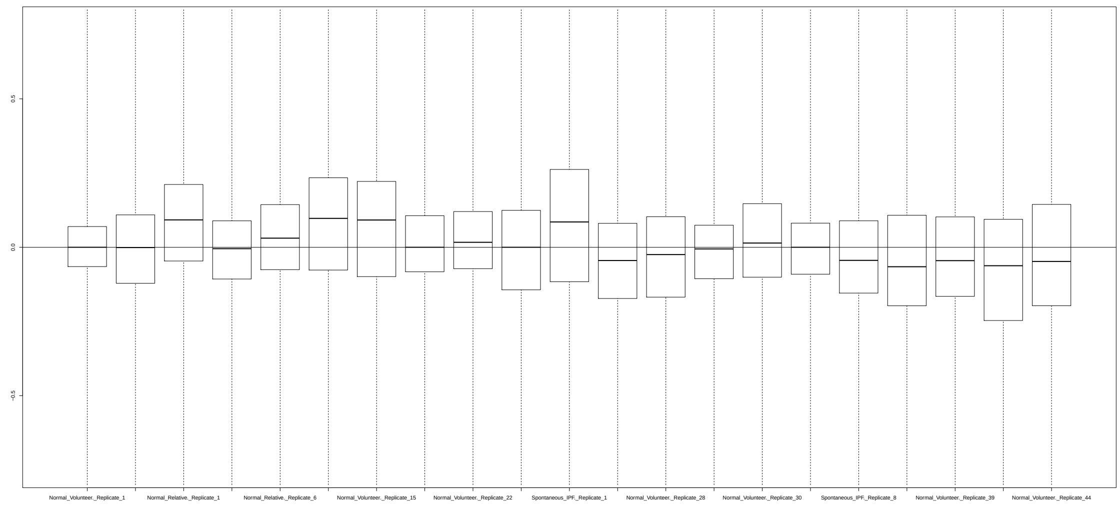

Sample Group [3]

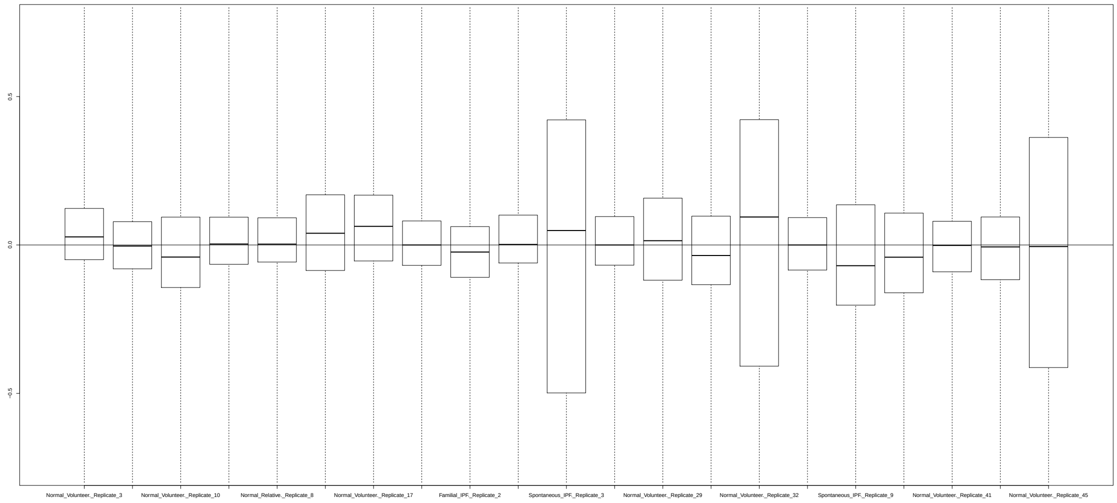

Sample Group [4]

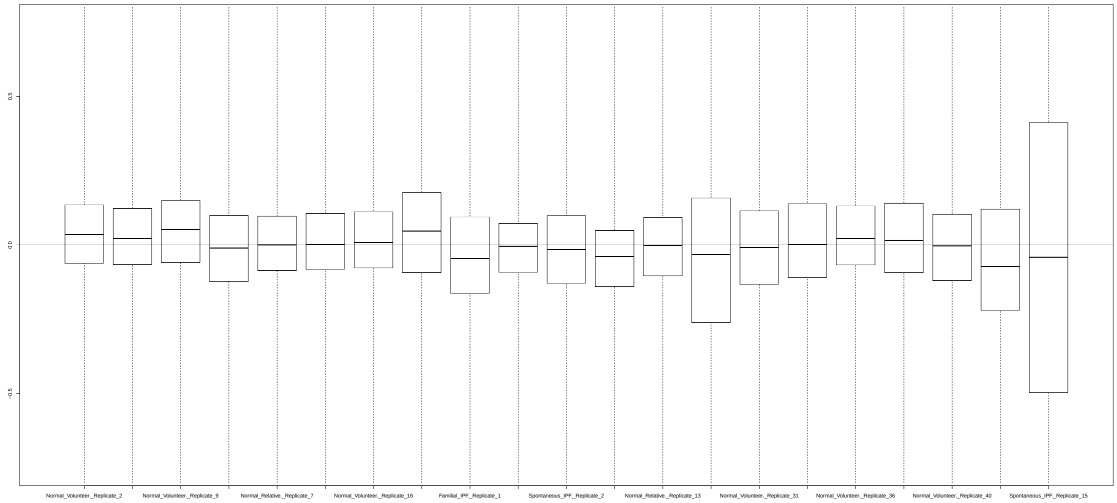

4 Normalized Unscaled Standard Errors

4.1 Summarized Median QC

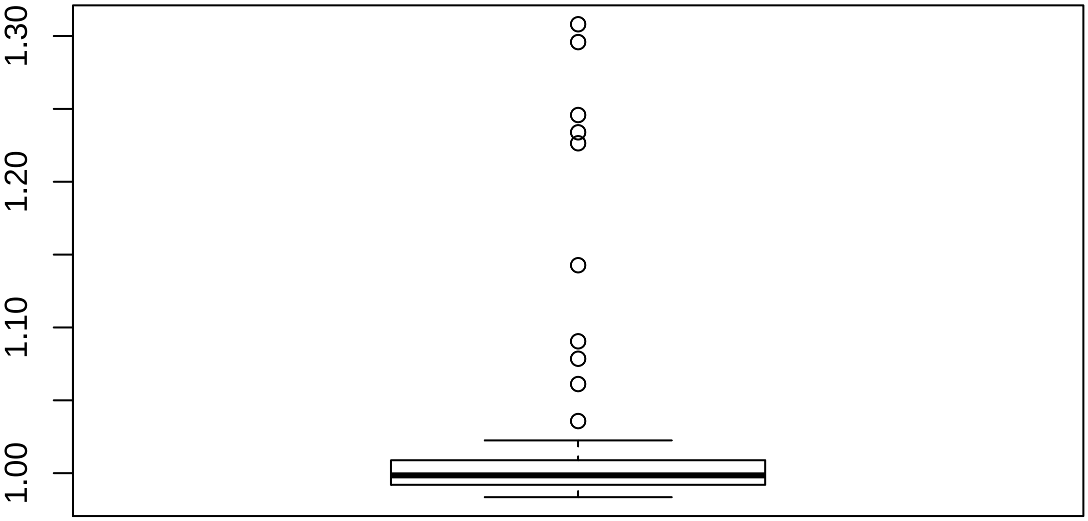

4.2 Discrete QC Plots

Sample Group [1]

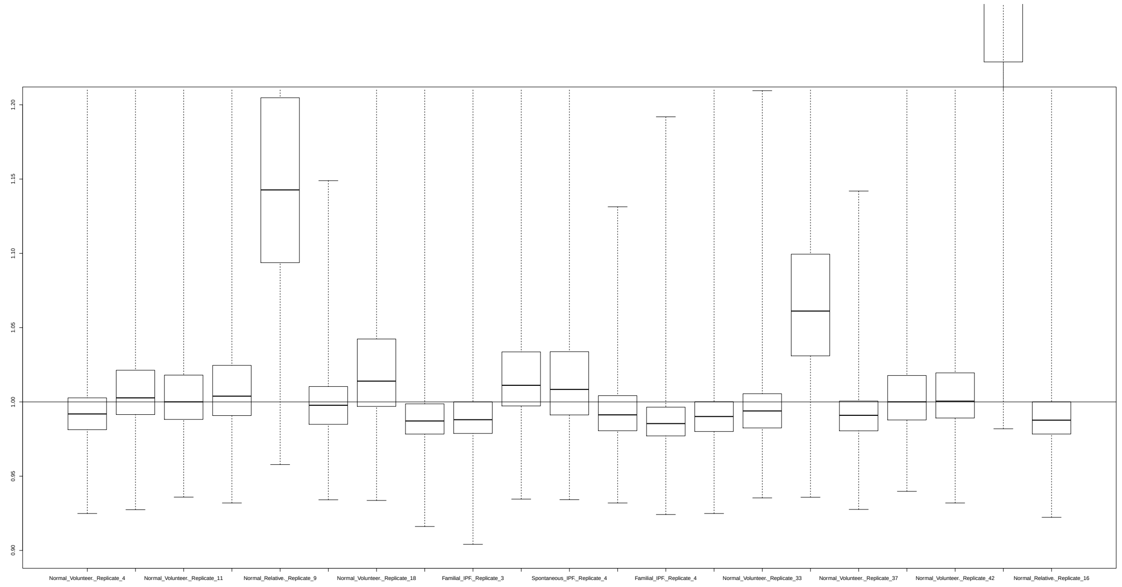

Sample Group [2]

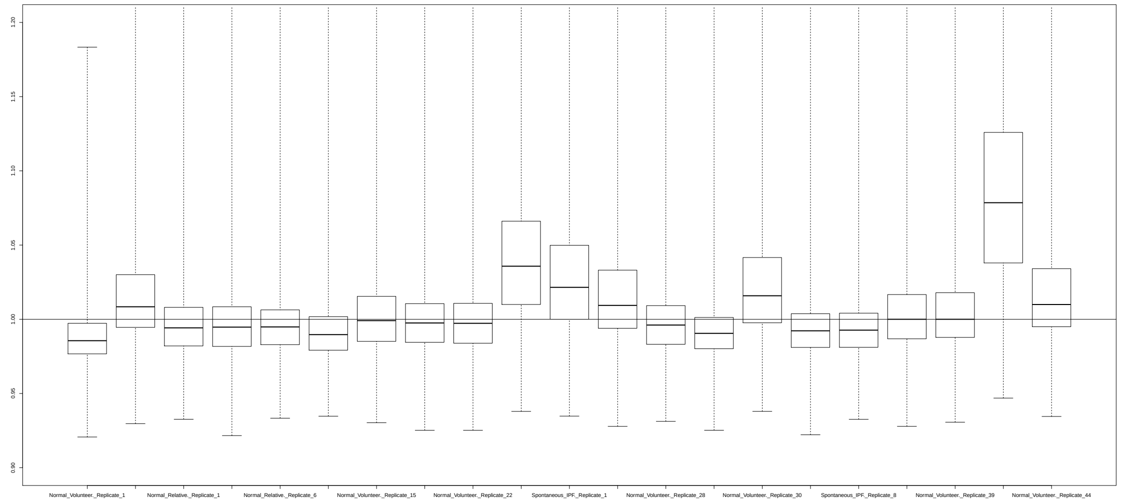

Sample Group [3]

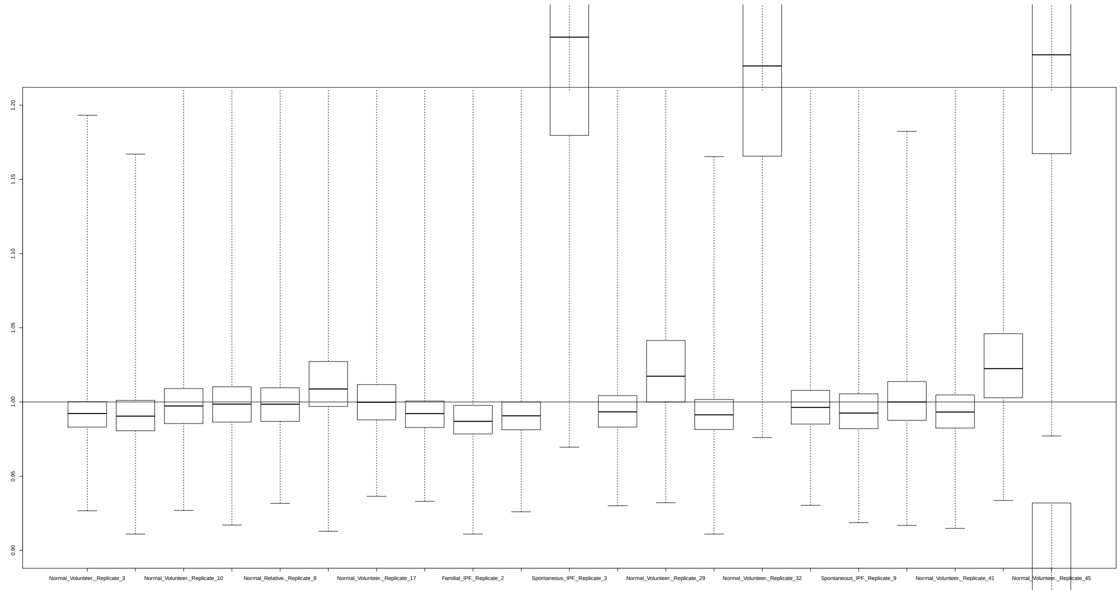

Sample Group [4]

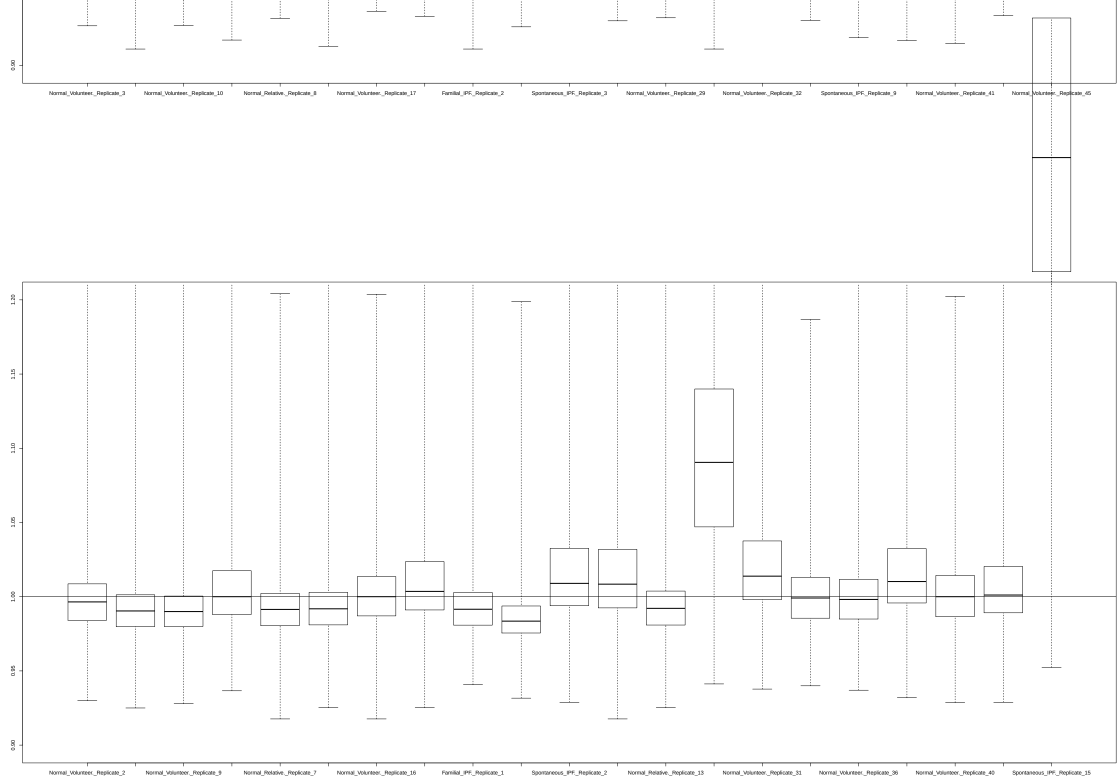

5 YAQC Plots

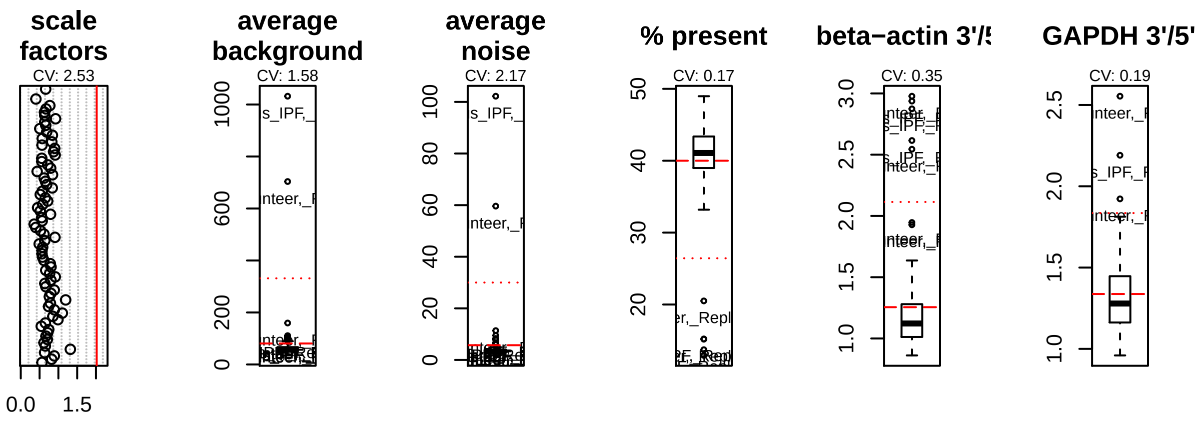

scale factors
CV: 2.53
0.0
1.5
average background
CV: 1.58
average noise
CV: 2.17
% present
CV: 0.17
beta−actin 3'/5'
CV: 0.35
GAPDH 3'/5'
CV: 0.19

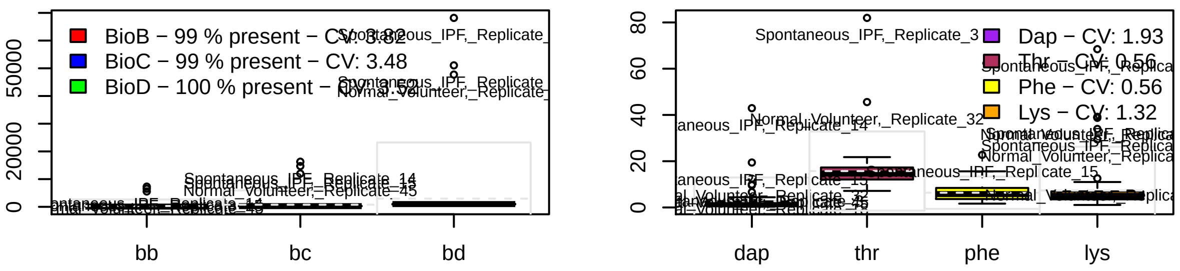

BioB − 99 % present − CV: 3.82
BioC − 99 % present − CV: 3.48
BioD − 100 % present − CV: 3.52
bb
bc
bd
Dap − CV: 1.93
Thr − CV: 0.56
Phe − CV: 0.56
Lys − CV: 1.32
dap
thr
phe
lys
