# Supplementary material for: Curated and harmonised transcriptomics datasets of interstitial lung diseases
Source: Data Brief. 2025 Oct 14;63:112139. doi: 10.1016/j.dib.2025.112139 (PMC12581653; doi:10.1016/j.dib.2025.112139)
Supplement: Supplementary file 1 [file mmc1.zip › Supplementary_material/DNA-microarray/GSE44723/GSE44723_eUTOPIA_Affymetrix_QC_Report_2024-02-12-3.pdf]

eUTOPIA Affymetrix QC Report

eUTOPIA

Contents

1 Outliers Table **1**
1.1 Outliers (All Methods) . . . 1
1.2 Outliers (At Least One Method) . . . 1

2 RNA Degradation **2**
2.1 Summarized Mean QC . . . 2
2.2 Discrete QC Plots . . . 3

3 Relative Log Expression **4**
3.1 Summarized Median QC . . . 4
3.2 Discrete QC Plots . . . 5

4 Normalized Unscaled Standard Errors **6**
4.1 Summarized Median QC . . . 6
4.2 Discrete QC Plots . . . 7

5 YAQC Plots **8**

1 Outliers Table

	RLE	NUSE	DEG	SUM
fibroblast__Normal__D133__Media__3	0	0	1	1
fibroblast__Rapid__D200__Media__14	0	0	1	1
fibroblast__Rapid__D26__Media__15	0	0	1	1
fibroblast__Rapid__D10__Media__13	0	1	0	1
fibroblast__Normal__D207__Media__6	0	1	0	1

1.1 Outliers (All Methods)

Outliers overall
NA

1.2 Outliers (At Least One Method)

Outliers at least 1
fibroblast__Normal__D133__Media__3
fibroblast__Rapid__D200__Media__14
fibroblast__Rapid__D26__Media__15
fibroblast__Rapid__D10__Media__13
fibroblast__Normal__D207__Media__6

2 RNA Degradation

2.1 Summarized Mean QC

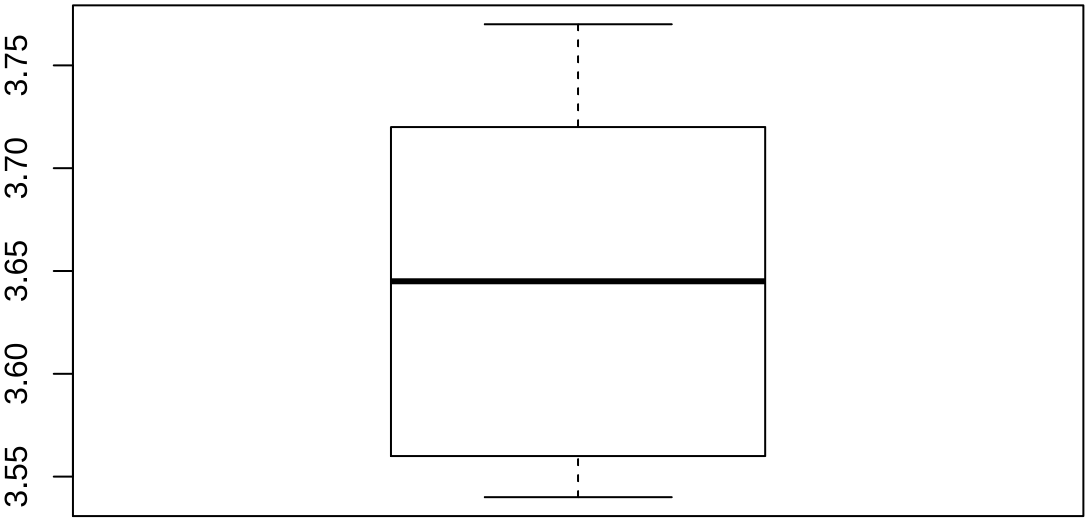

2.2 Discrete QC Plots

Sample Group [1]

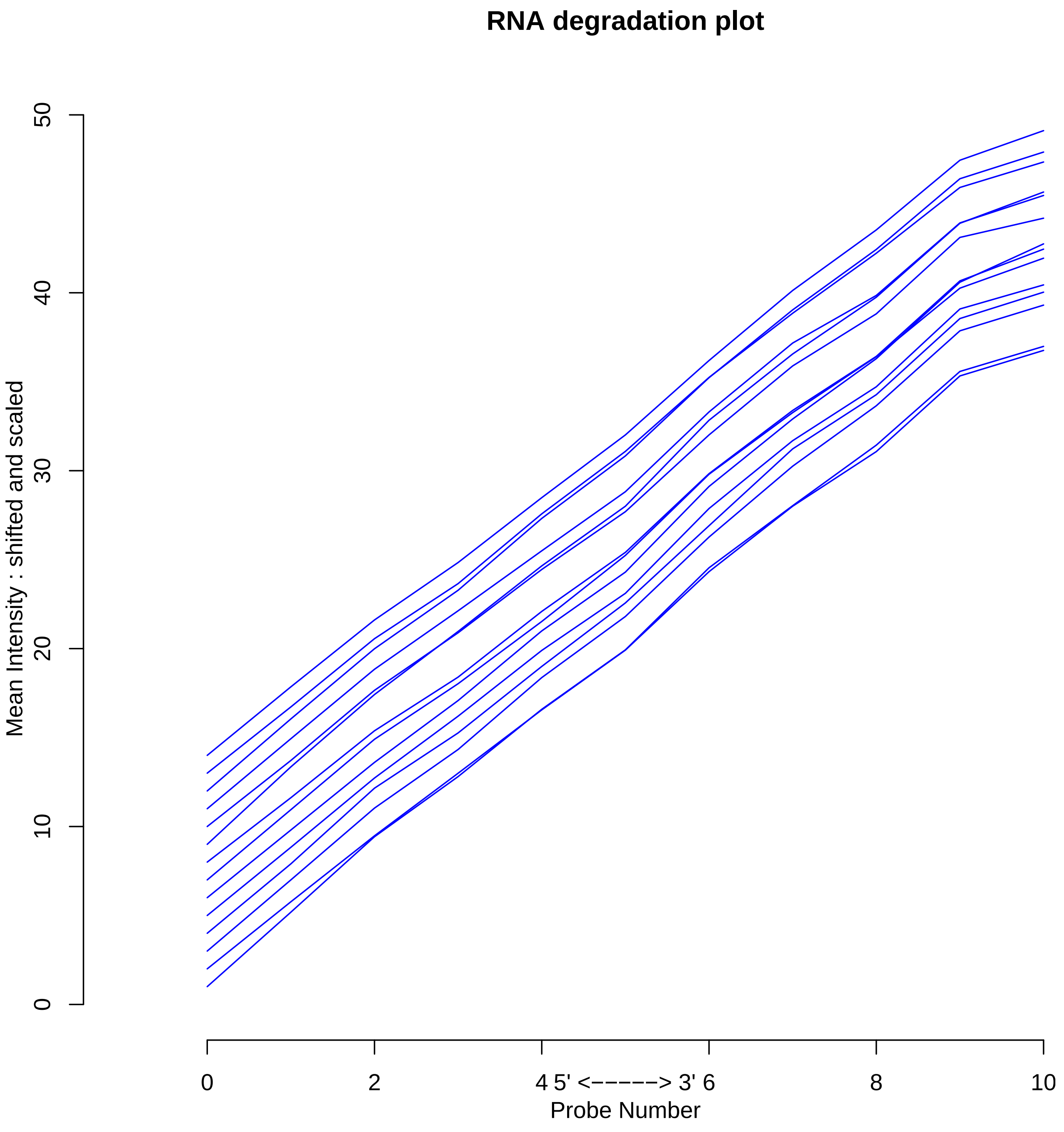

3 Relative Log Expression

3.1 Summarized Median QC

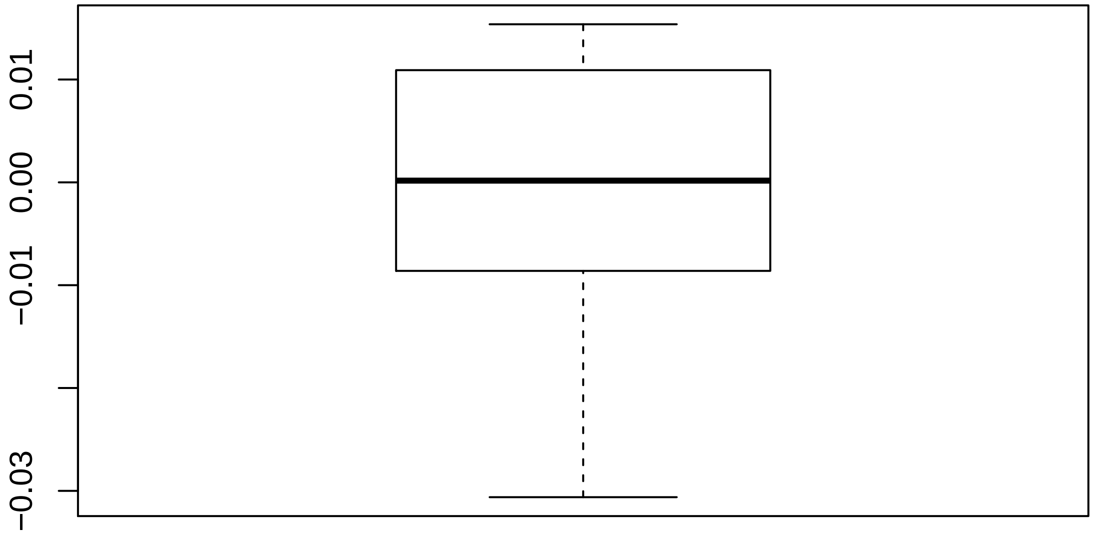

3.2 Discrete QC Plots

Sample Group [1]

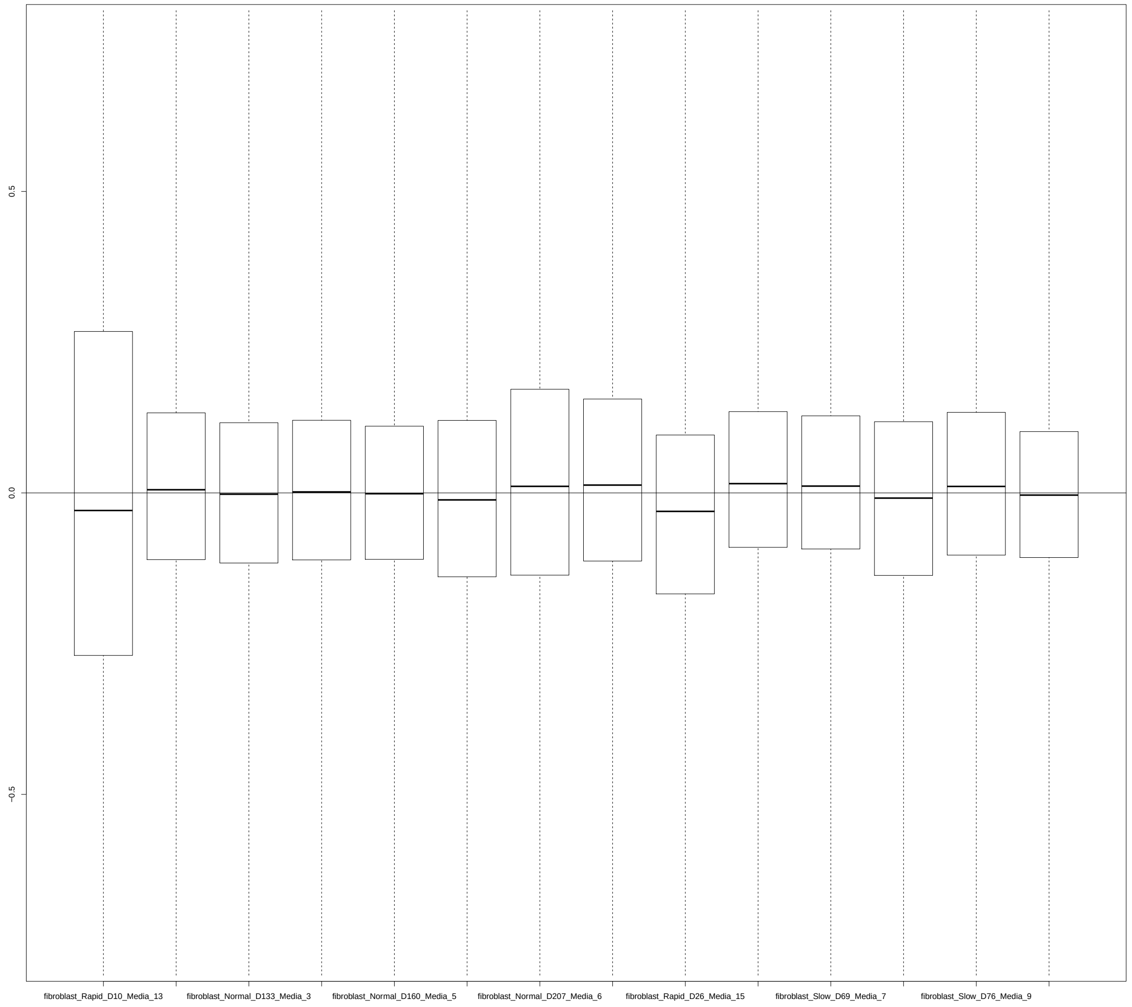

4 Normalized Unscaled Standard Errors

4.1 Summarized Median QC

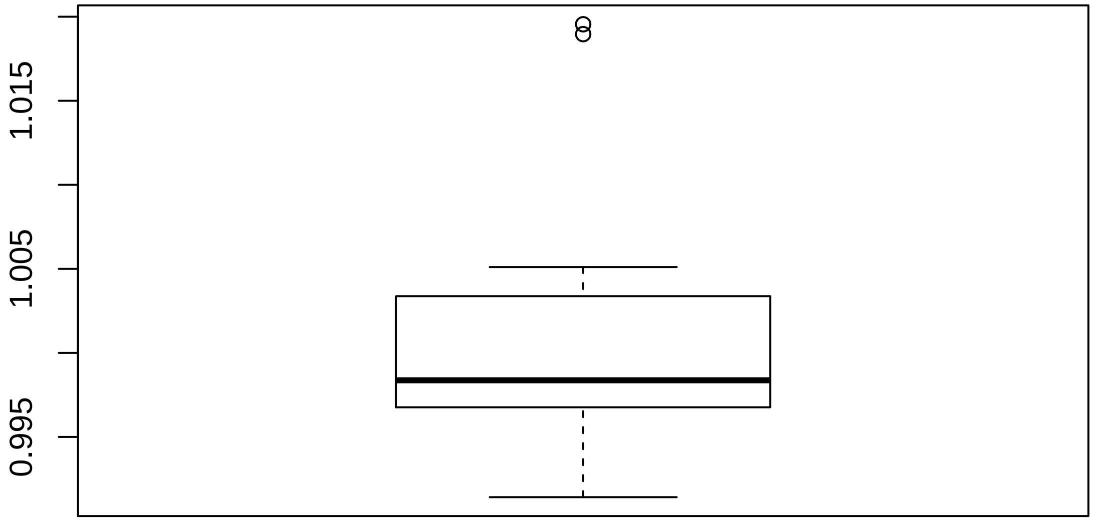

4.2 Discrete QC Plots

Sample Group [1]

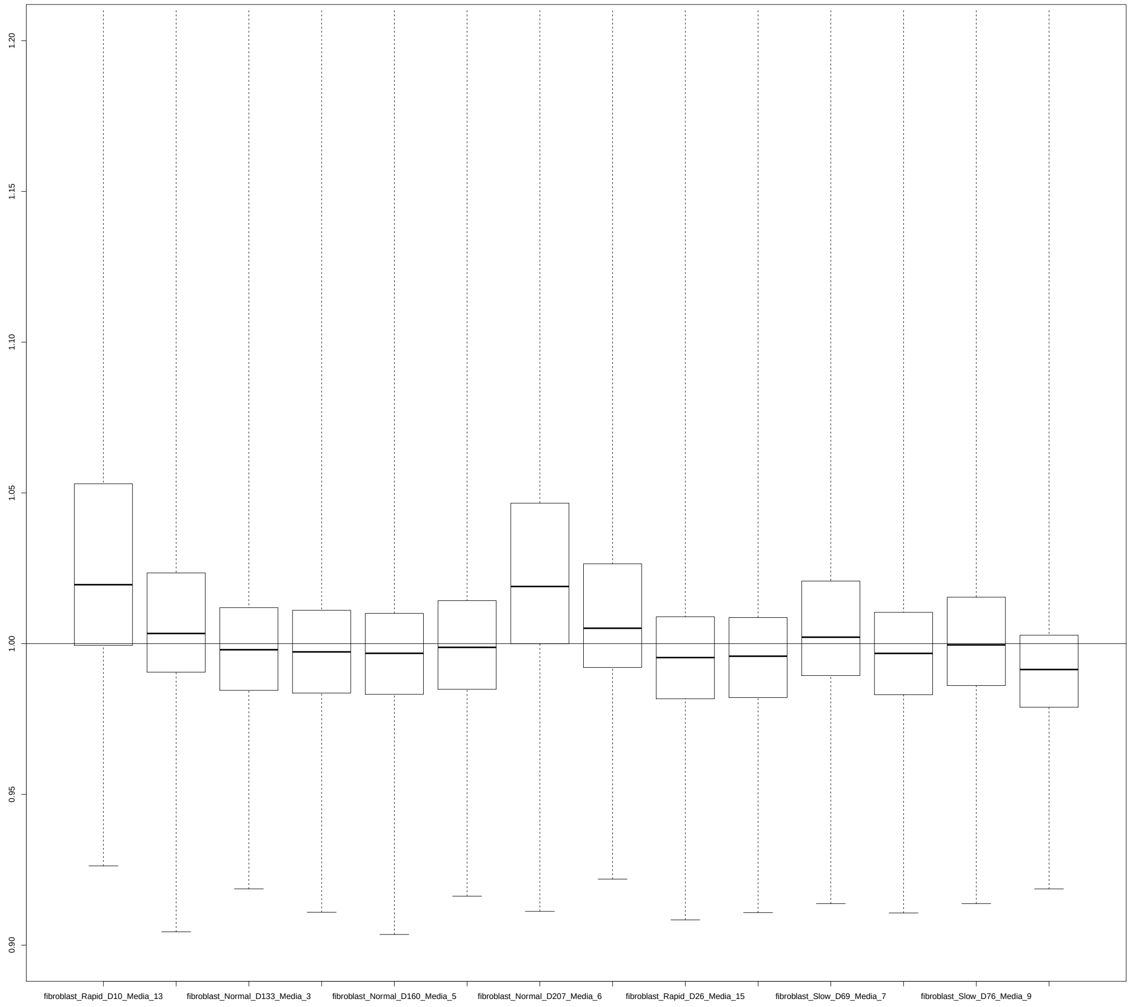

5 YAQC Plots

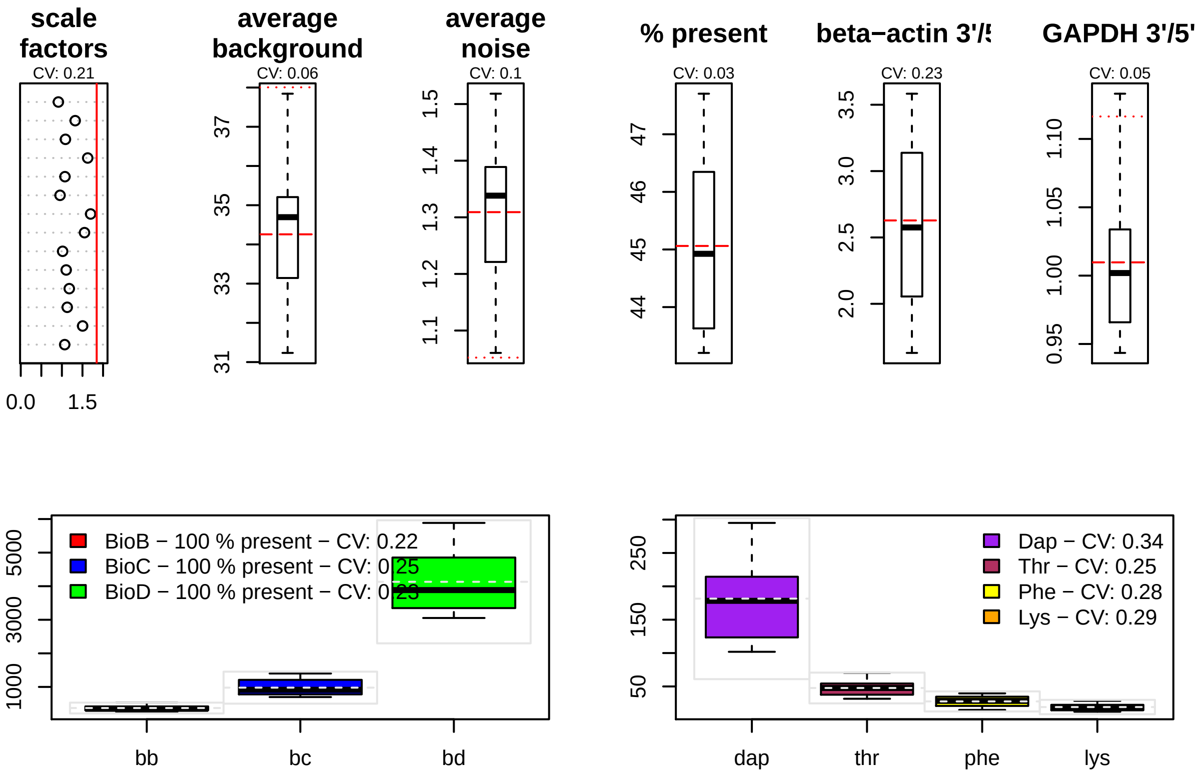

scale factors
CV: 0.21
average background
CV: 0.06
average noise
CV: 0.1
% present
CV: 0.03
beta−actin 3'/5'
CV: 0.23
GAPDH 3'/5'
CV: 0.05
BioB − 100 % present − CV: 0.22
BioC − 100 % present − CV: 0.25
BioD − 100 % present − CV: 0.23
bb
bc
bd
Dap − CV: 0.34
Thr − CV: 0.25
Phe − CV: 0.28
Lys − CV: 0.29
dap
thr
phe
lys
