# Supplementary material for: Curated and harmonised transcriptomics datasets of interstitial lung diseases
Source: Data Brief. 2025 Oct 14;63:112139. doi: 10.1016/j.dib.2025.112139 (PMC12581653; doi:10.1016/j.dib.2025.112139)

eUTOPIA Affymetrix QC Report

eUTOPIA

Contents

1 Outliers Table **1**
1.1 Outliers (All Methods) . . . 1
1.2 Outliers (At Least One Method) . . . 1

2 RNA Degradation **2**
2.1 Summarized Mean QC . . . 2
2.2 Discrete QC Plots . . . 3

3 Relative Log Expression **4**
3.1 Summarized Median QC . . . 4
3.2 Discrete QC Plots . . . 5

4 Normalized Unscaled Standard Errors **6**
4.1 Summarized Median QC . . . 6
4.2 Discrete QC Plots . . . 7

1 Outliers Table

	RLE	NUSE	DEG	SUM
Control_MDM_replicate_1	0	0	1	1
Control_MDM_replicate_2	0	0	1	1
Control_MDM_replicate_3	1	0	1	2
Control_MDM_replicate_4	0	0	1	1
MDM_co_cultured_with_AN_replicate_1	0	0	1	1
AM_from_RB_ILD_patient_1	0	0	1	1
MDM_co_cultured_with_AN_replicate_3	0	1	0	1
AM_from_RB_ILD_patient_2	0	1	0	1

1.1 Outliers (All Methods)

Outliers overall
Control_MDM_replicate_3

1.2 Outliers (At Least One Method)

Outliers at least 1
Control_MDM_replicate_1
Control_MDM_replicate_2
Control_MDM_replicate_3
Control_MDM_replicate_4
MDM_co_cultured_with_AN_replicate_1
AM_from_RB_ILD_patient_1

Outliers at least 1
MDM_co_cultured_with_AN_replicate_3
AM_from_RB_ILD_patient_2

2 RNA Degradation

2.1 Summarized Mean QC

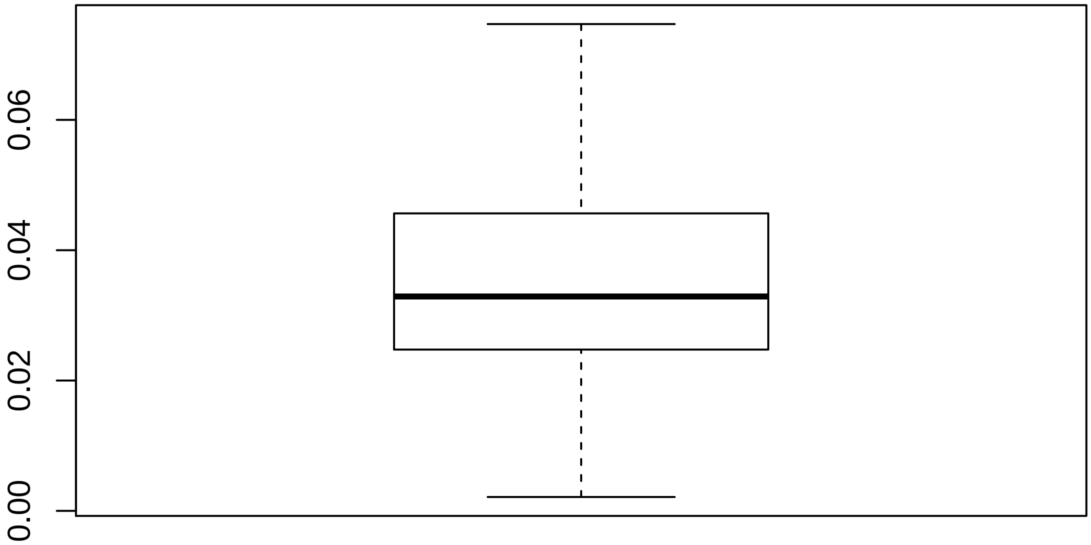

2.2 Discrete QC Plots

Sample Group [1]

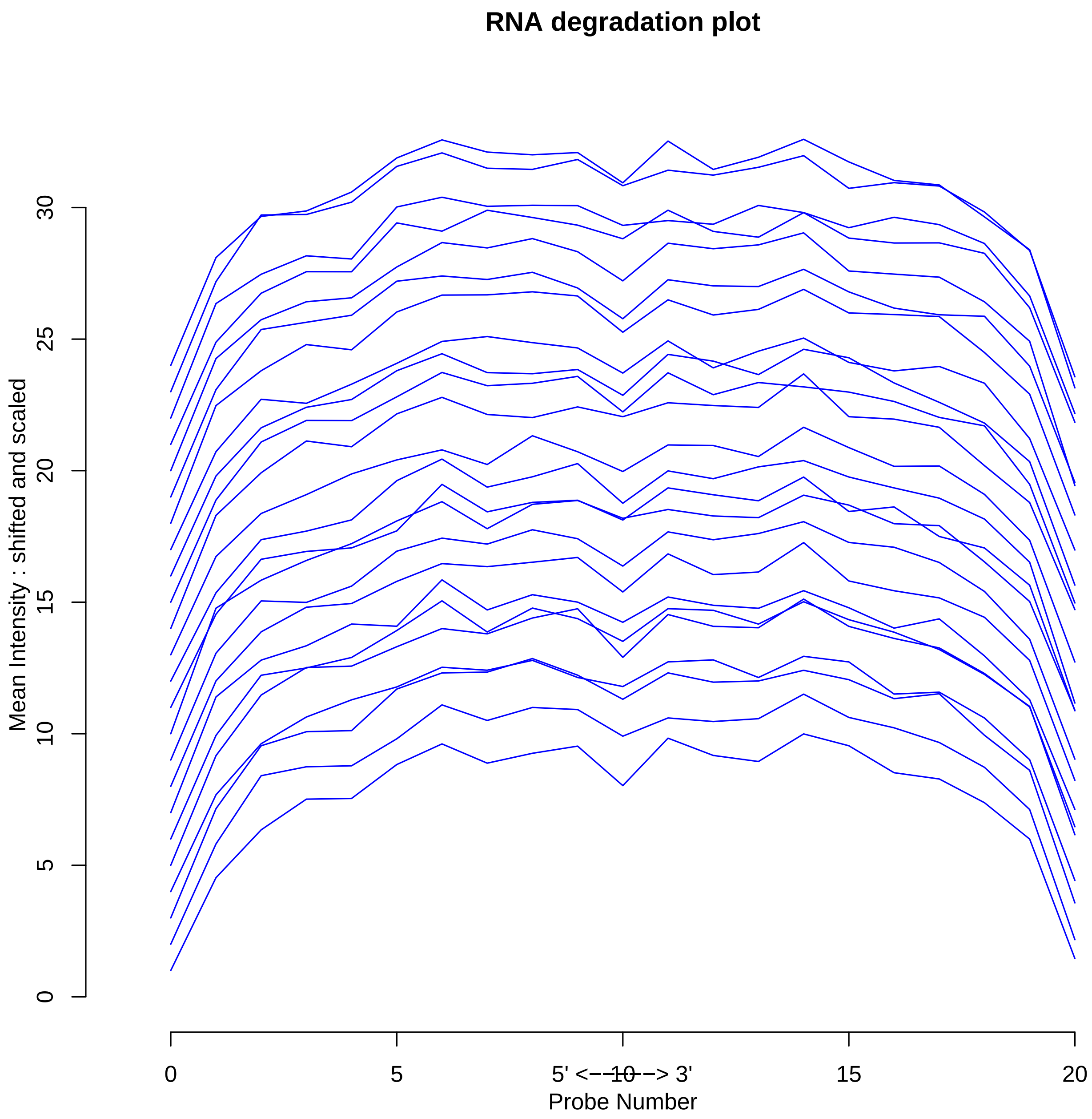

3 Relative Log Expression

3.1 Summarized Median QC

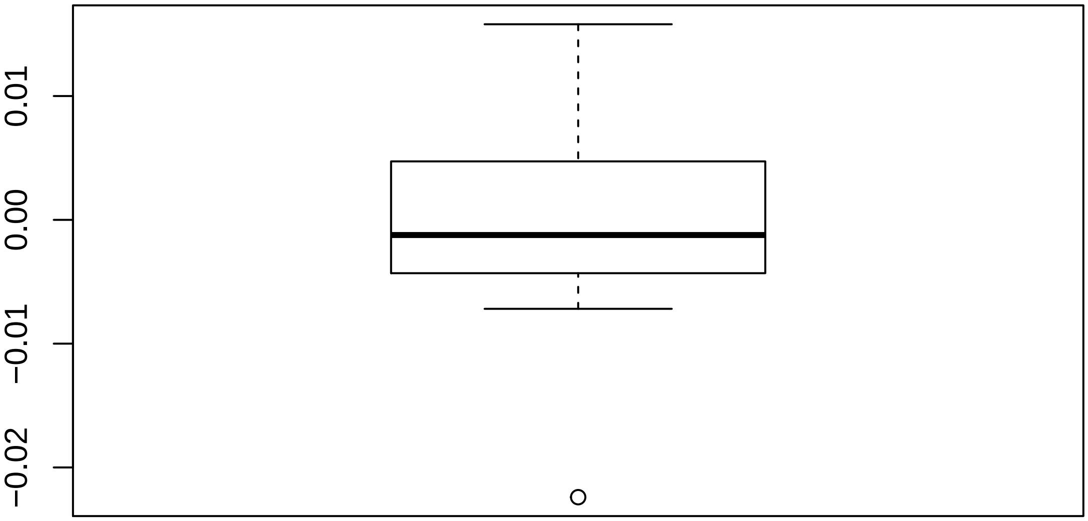

3.2 Discrete QC Plots

Sample Group [1]

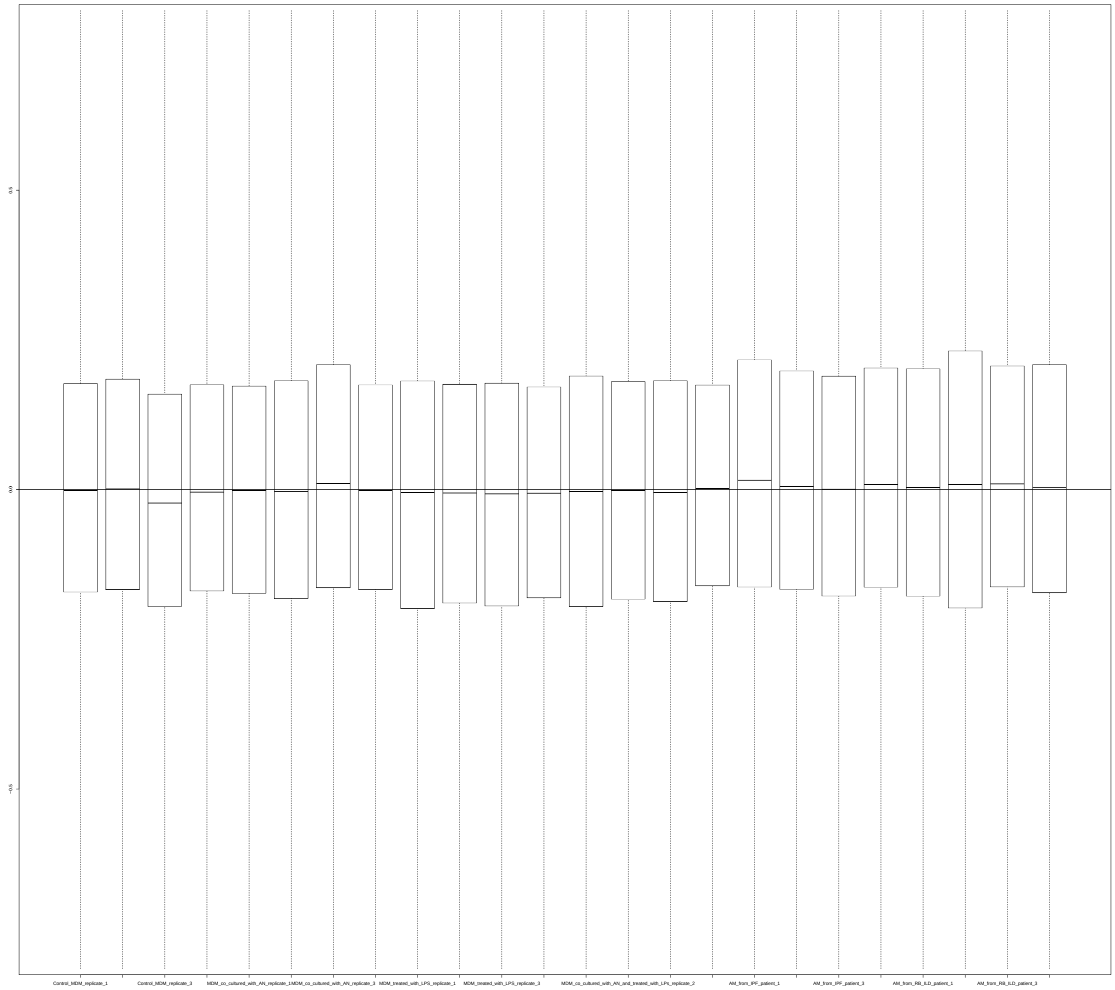

4 Normalized Unscaled Standard Errors

4.1 Summarized Median QC

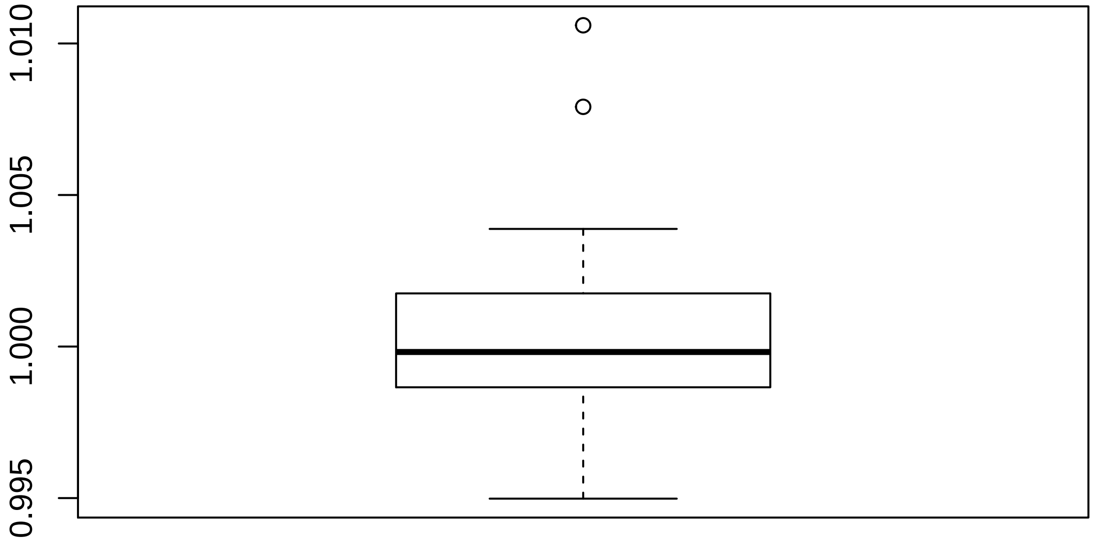

4.2 Discrete QC Plots

Sample Group [1]

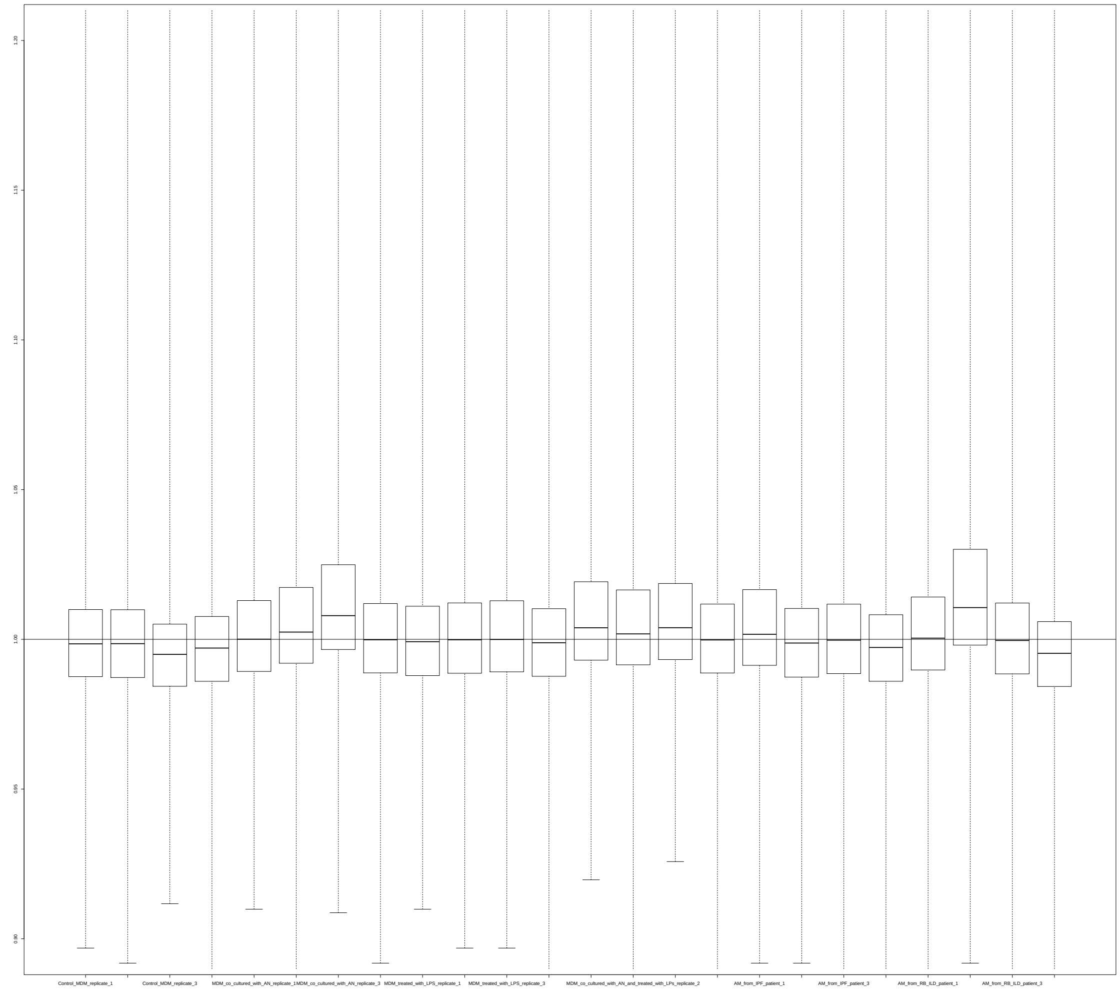

Supplement: Supplementary file 1 [file mmc1.zip › Supplementary_material/DNA-microarray/GSE90010/GSE90010_eUTOPIA_Affymetrix_QC_Report_2024-02-12-4.pdf]
