# Supplementary figures and images for: Curated and harmonised transcriptomics datasets of interstitial lung diseases
Source: Data Brief. 2025 Oct 14;63:112139. doi: 10.1016/j.dib.2025.112139 (PMC12581653; doi:10.1016/j.dib.2025.112139)

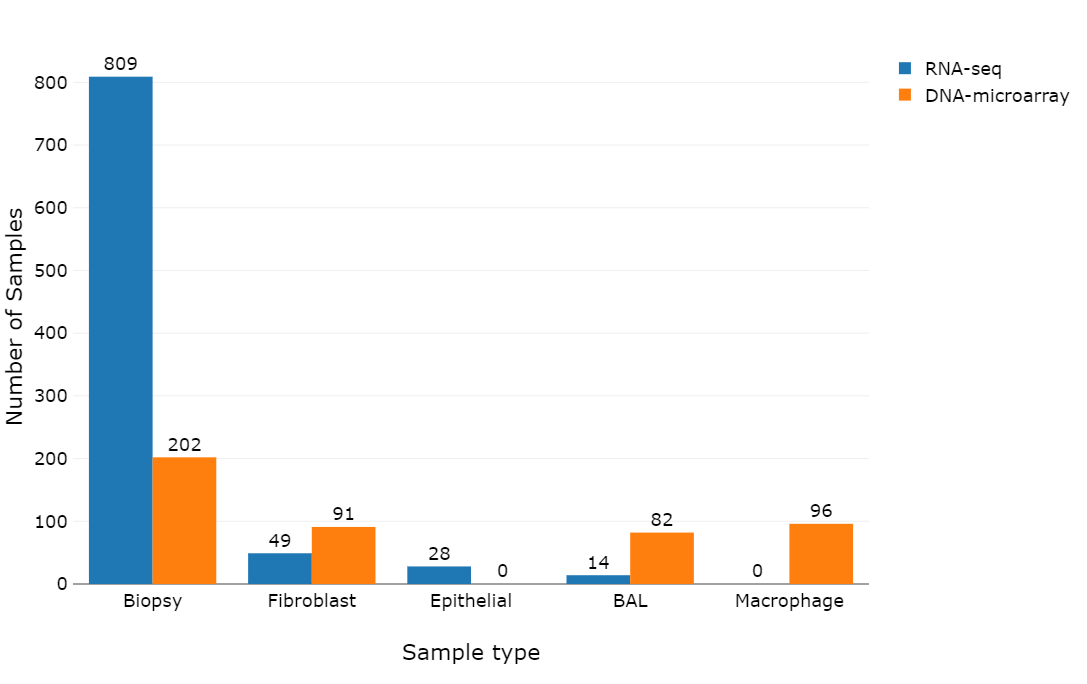

Supplement: Supplementary file 1 [file mmc1.zip › Supplementary_material/Supplementary_figure_1.png]

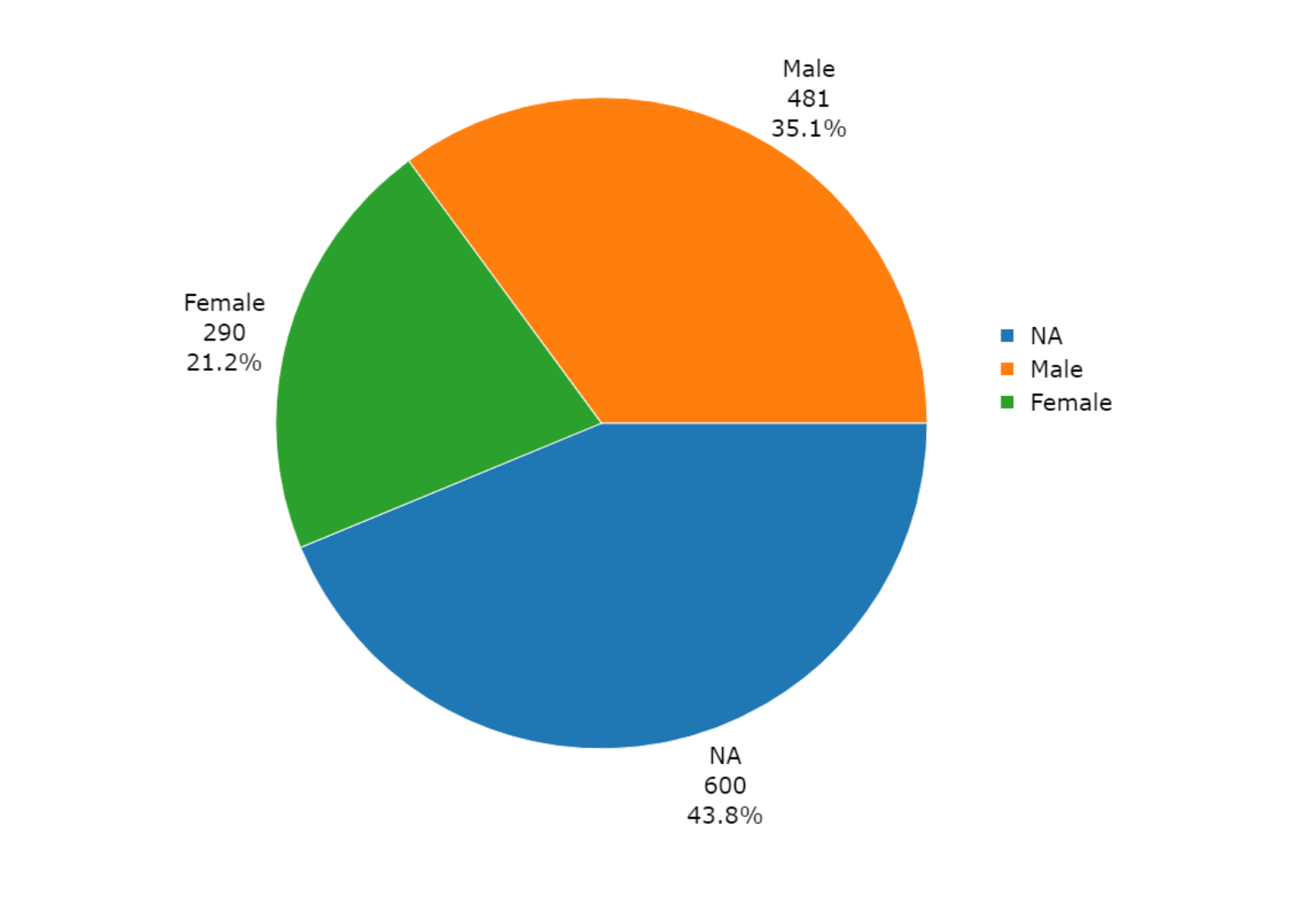

Supplement: Supplementary file 1 [file mmc1.zip › Supplementary_material/Supplementary_figure_2.png]

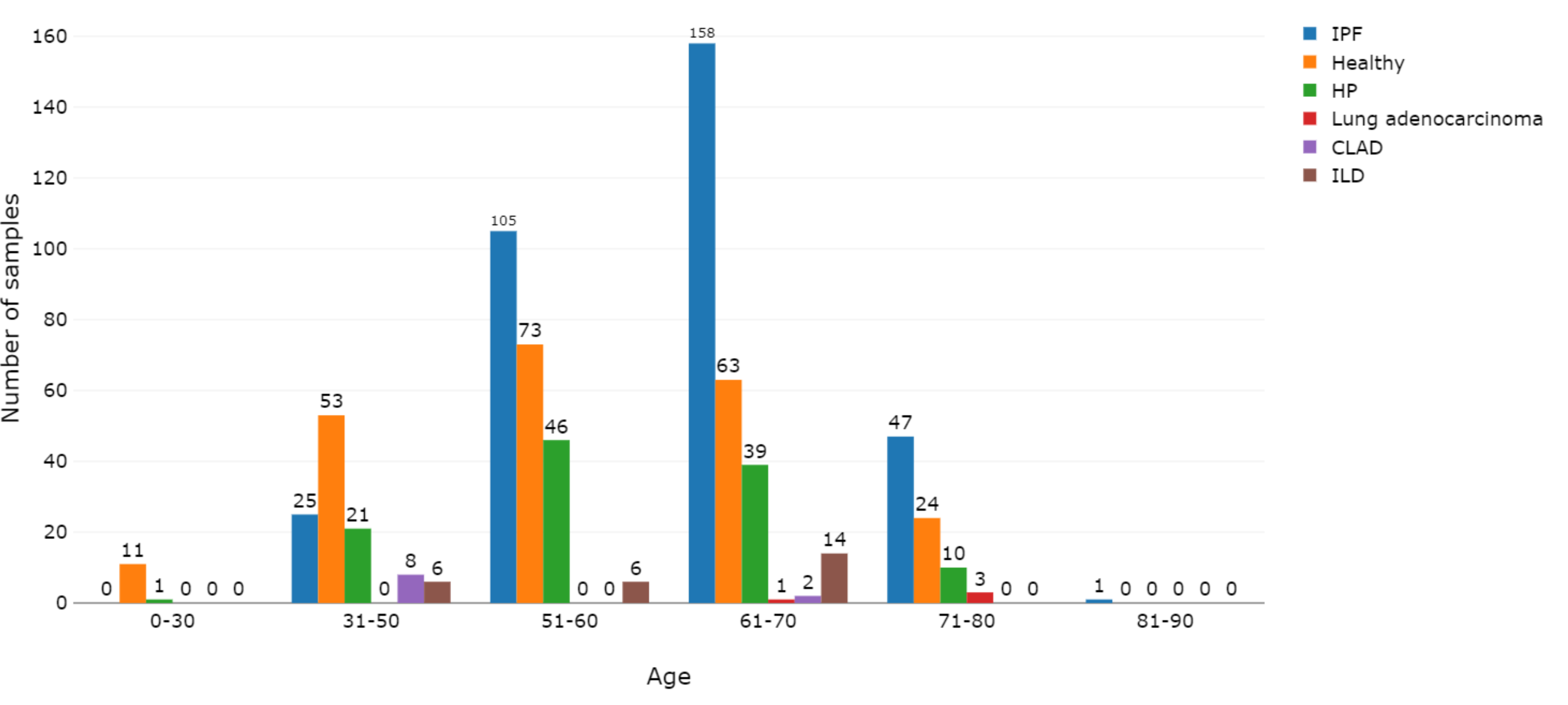

Supplement: Supplementary file 1 [file mmc1.zip › Supplementary_material/Supplementary_figure_3.png]

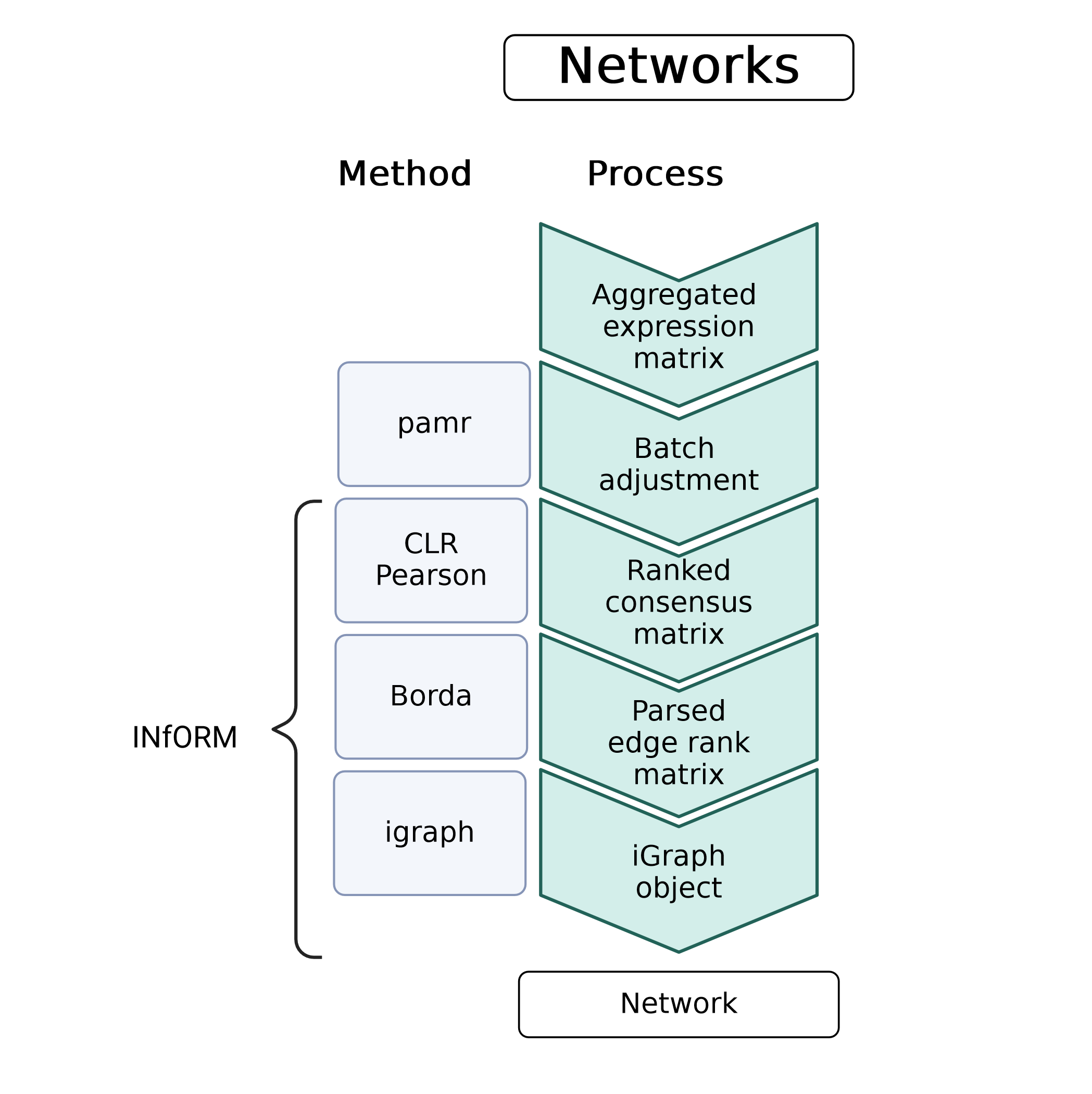

Supplement: Supplementary file 1 [file mmc1.zip › Supplementary_material/Supplementary_figure_7.png]

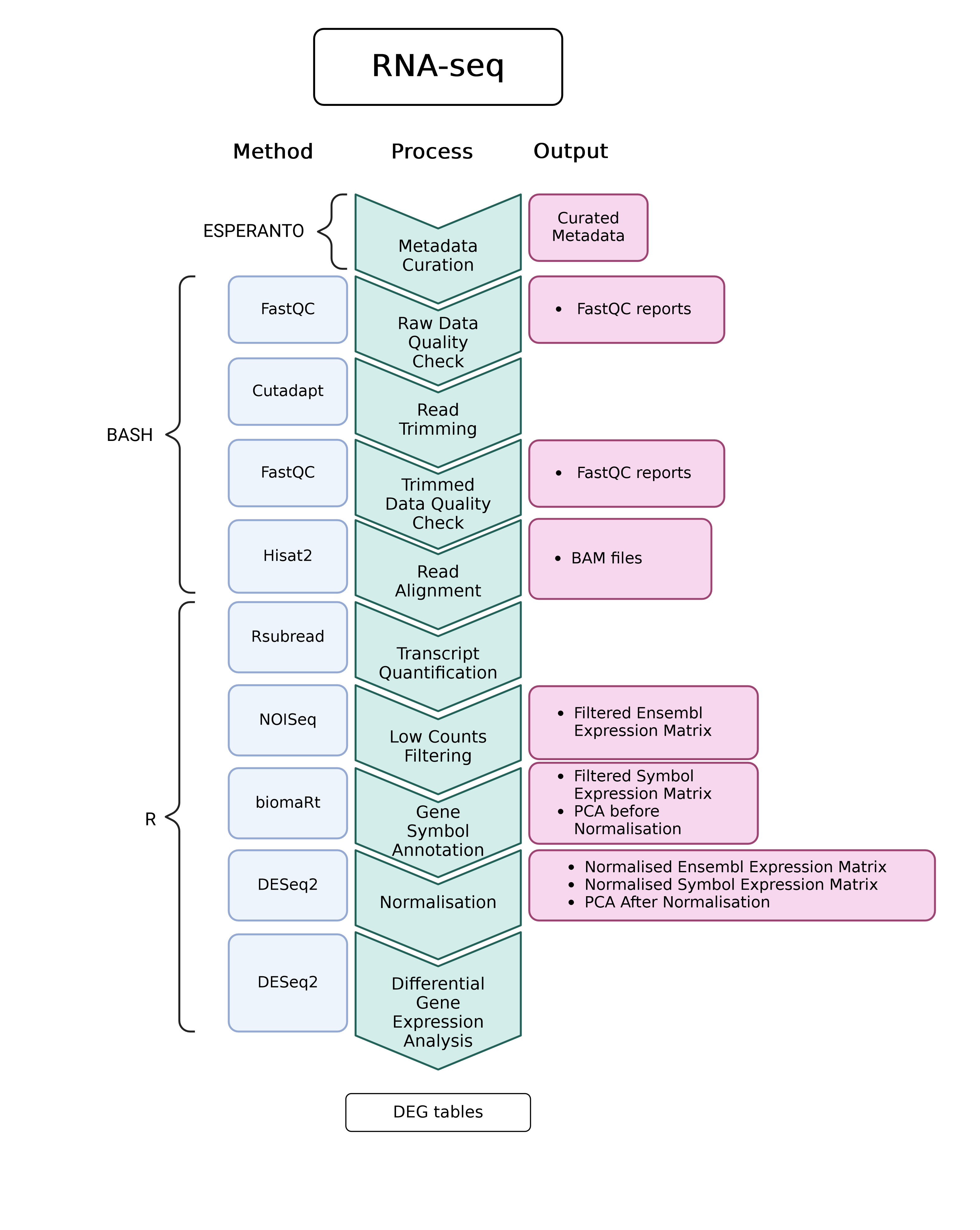

Supplement: Supplementary file 1 [file mmc1.zip › Supplementary_material/Supplementary_figure_6.png]

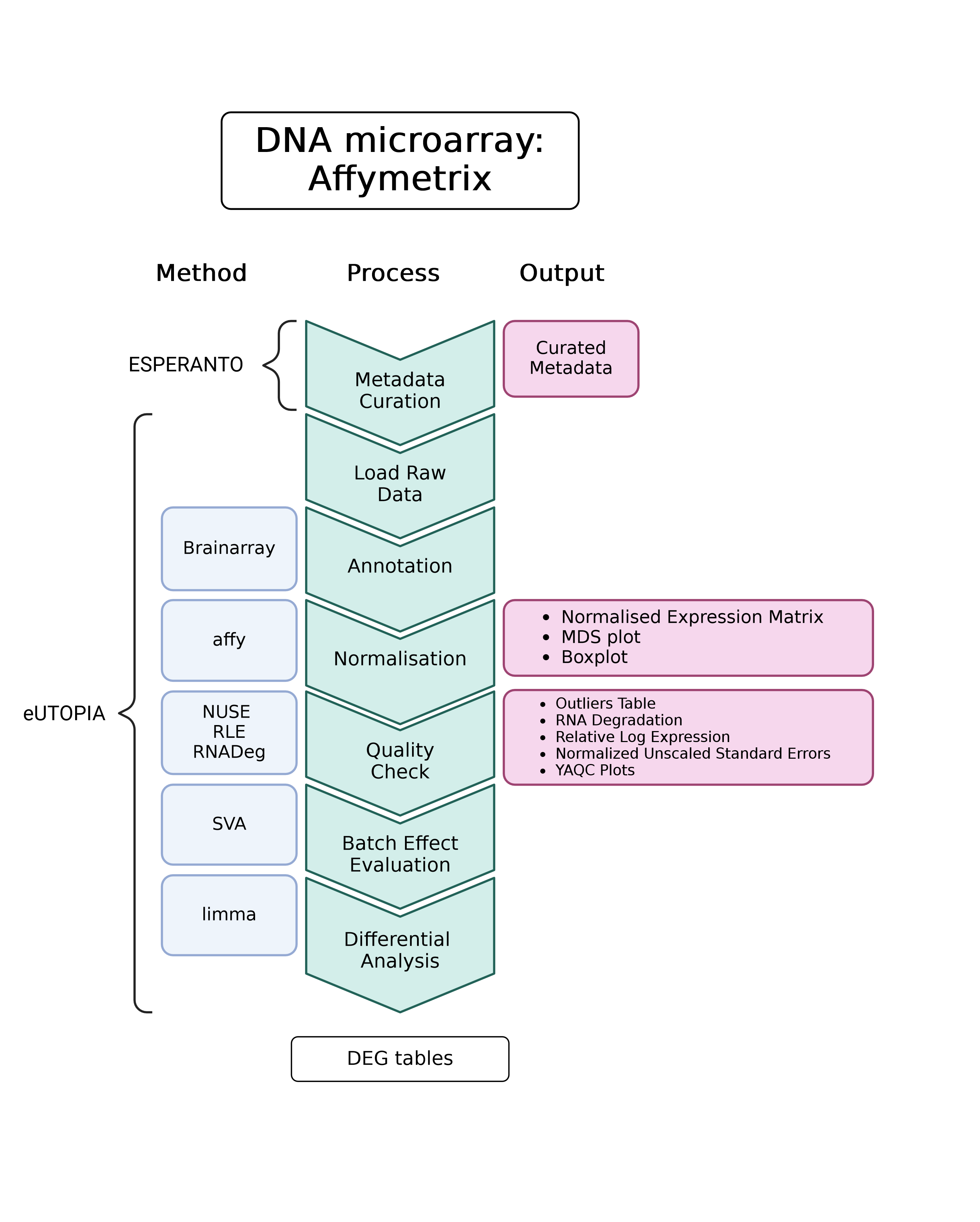

Supplement: Supplementary file 1 [file mmc1.zip › Supplementary_material/Supplementary_figure_4.png]

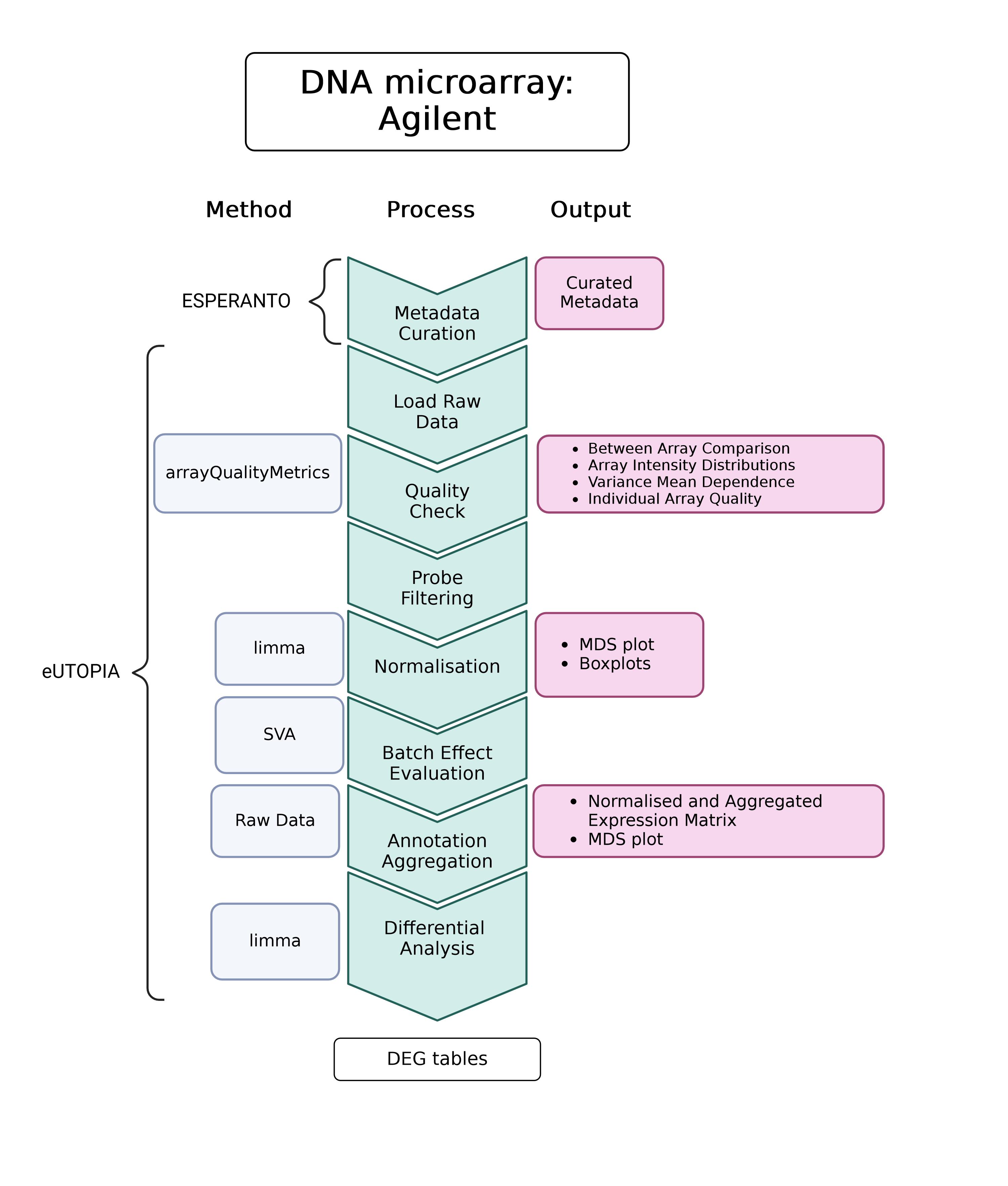

Supplement: Supplementary file 1 [file mmc1.zip › Supplementary_material/Supplementary_figure_5.png]

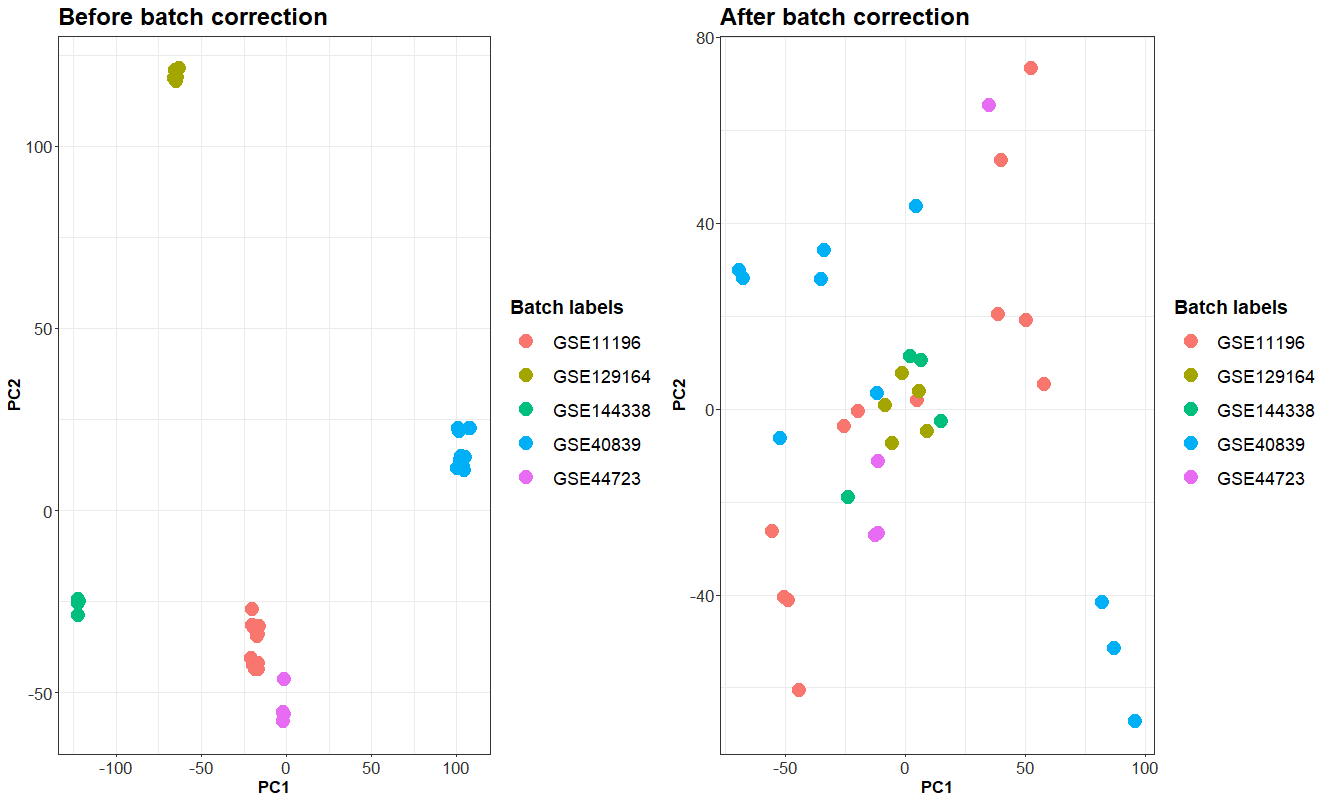

Supplement: Supplementary file 1 [file mmc1.zip › Supplementary_material/multi_studies_batch_adjust_pca_plots/fibroblast_healthy_microarray.png]

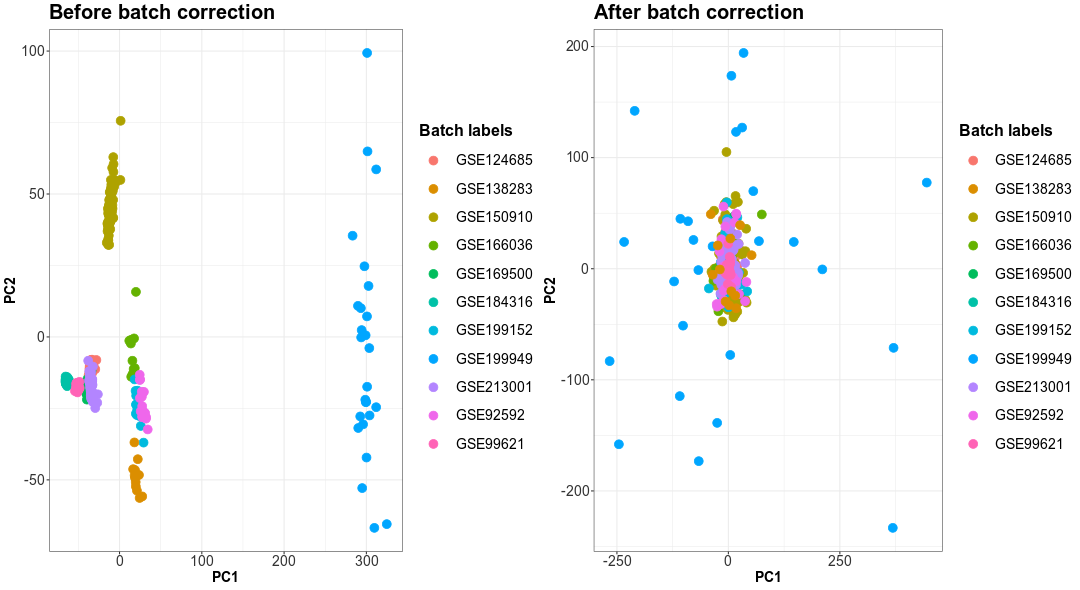

Supplement: Supplementary file 1 [file mmc1.zip › Supplementary_material/multi_studies_batch_adjust_pca_plots/biopsy_disease_RNAseq.png]

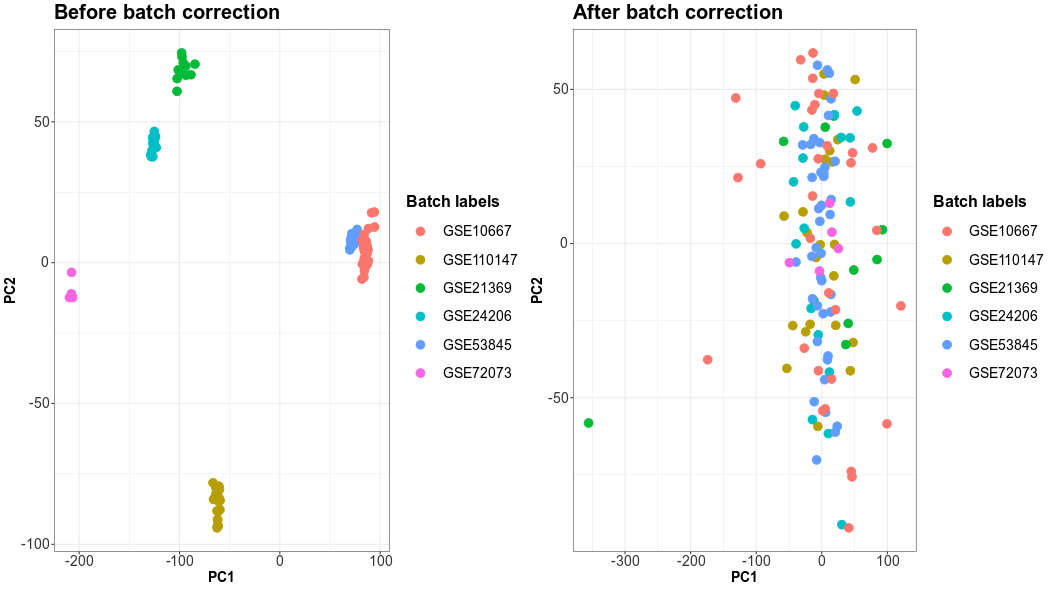

Supplement: Supplementary file 1 [file mmc1.zip › Supplementary_material/multi_studies_batch_adjust_pca_plots/biopsy_disease_microarray.png]

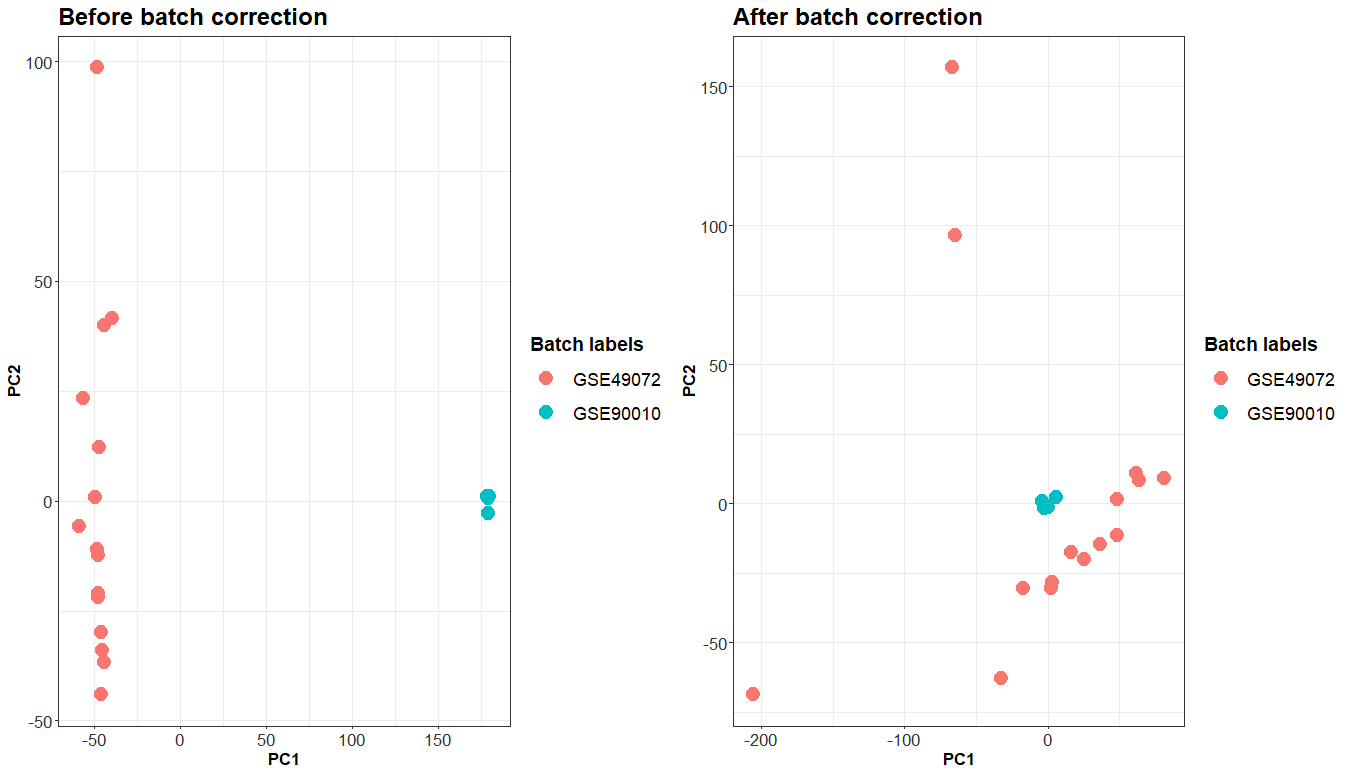

Supplement: Supplementary file 1 [file mmc1.zip › Supplementary_material/multi_studies_batch_adjust_pca_plots/macrophage_disease_microarray.png]

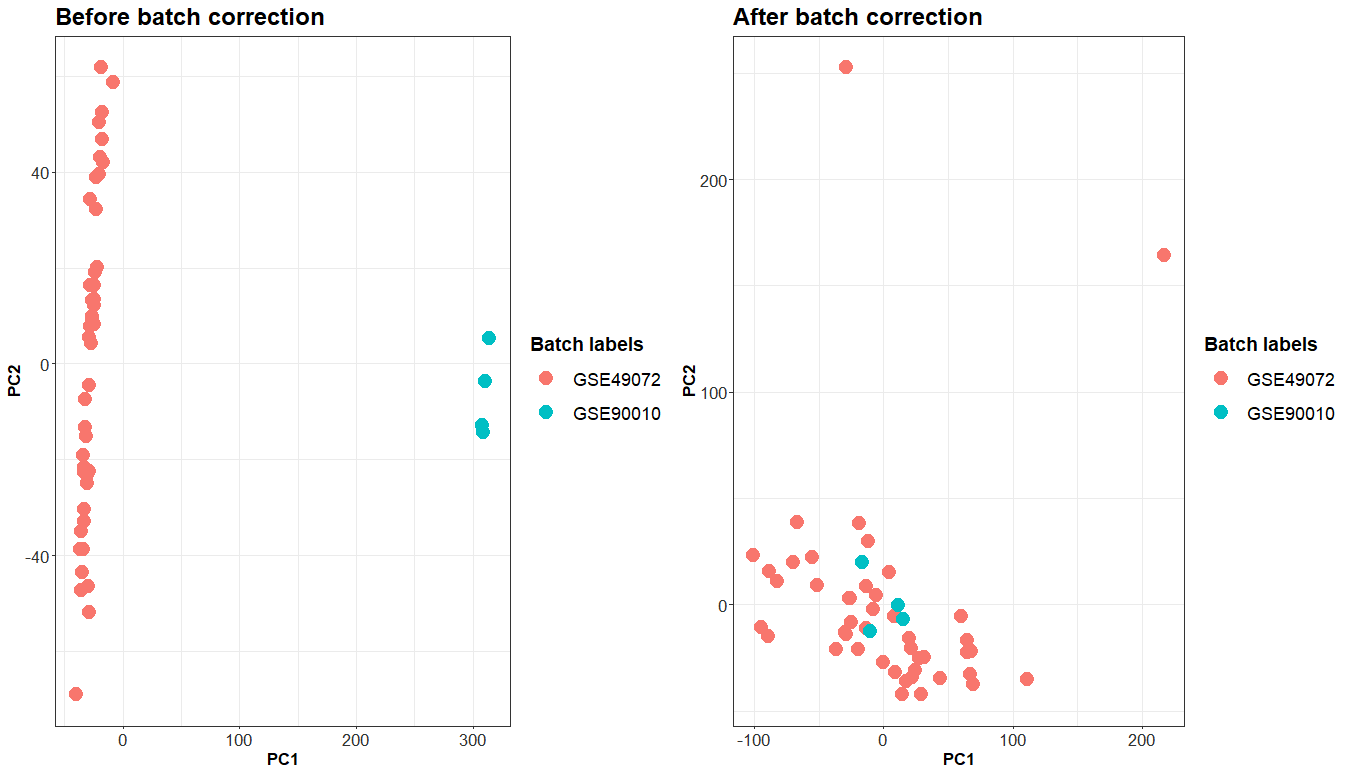

Supplement: Supplementary file 1 [file mmc1.zip › Supplementary_material/multi_studies_batch_adjust_pca_plots/macrophage_healthy_microarray.png]

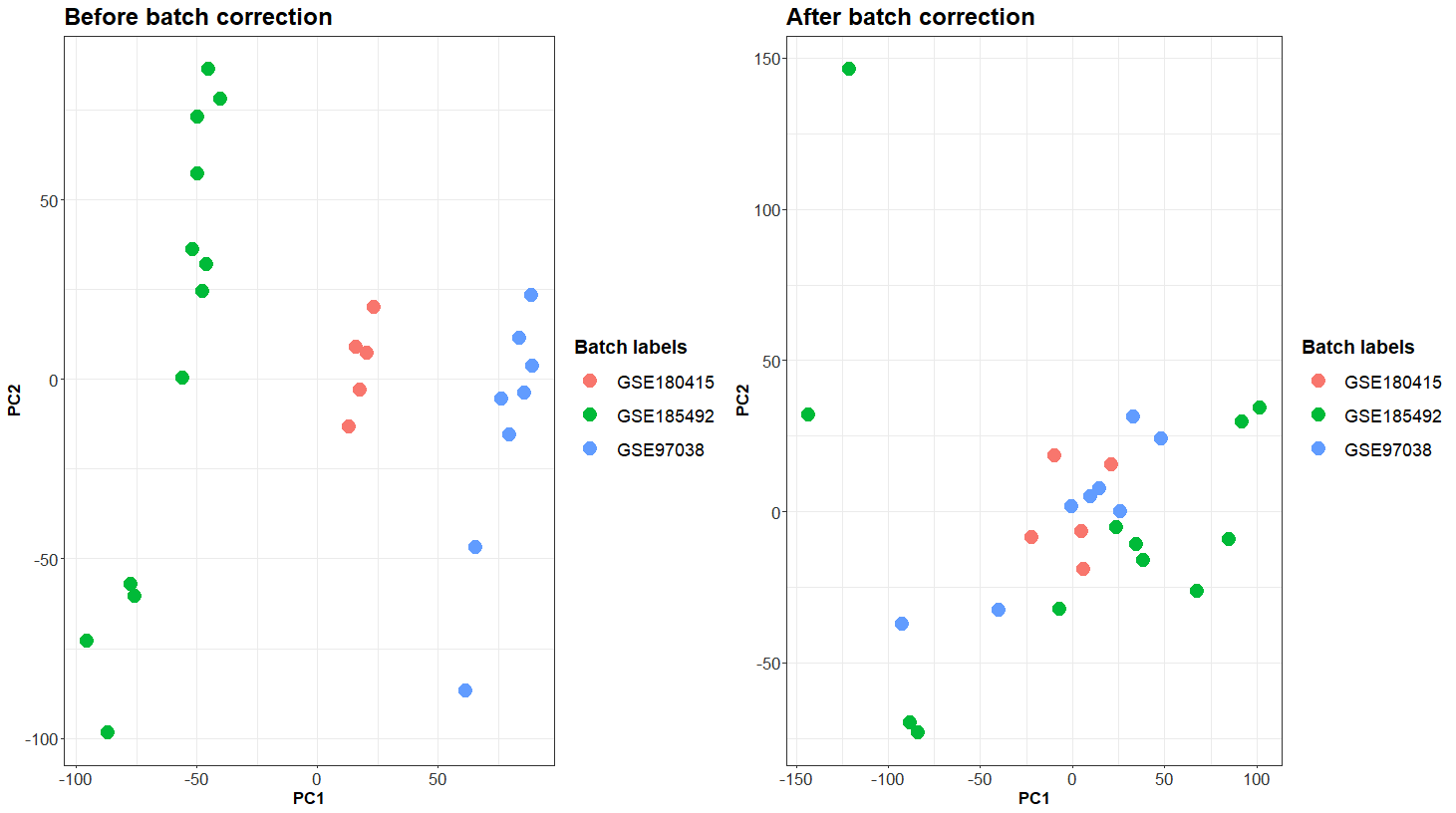

Supplement: Supplementary file 1 [file mmc1.zip › Supplementary_material/multi_studies_batch_adjust_pca_plots/fibroblast_disease_RNAseq.png]

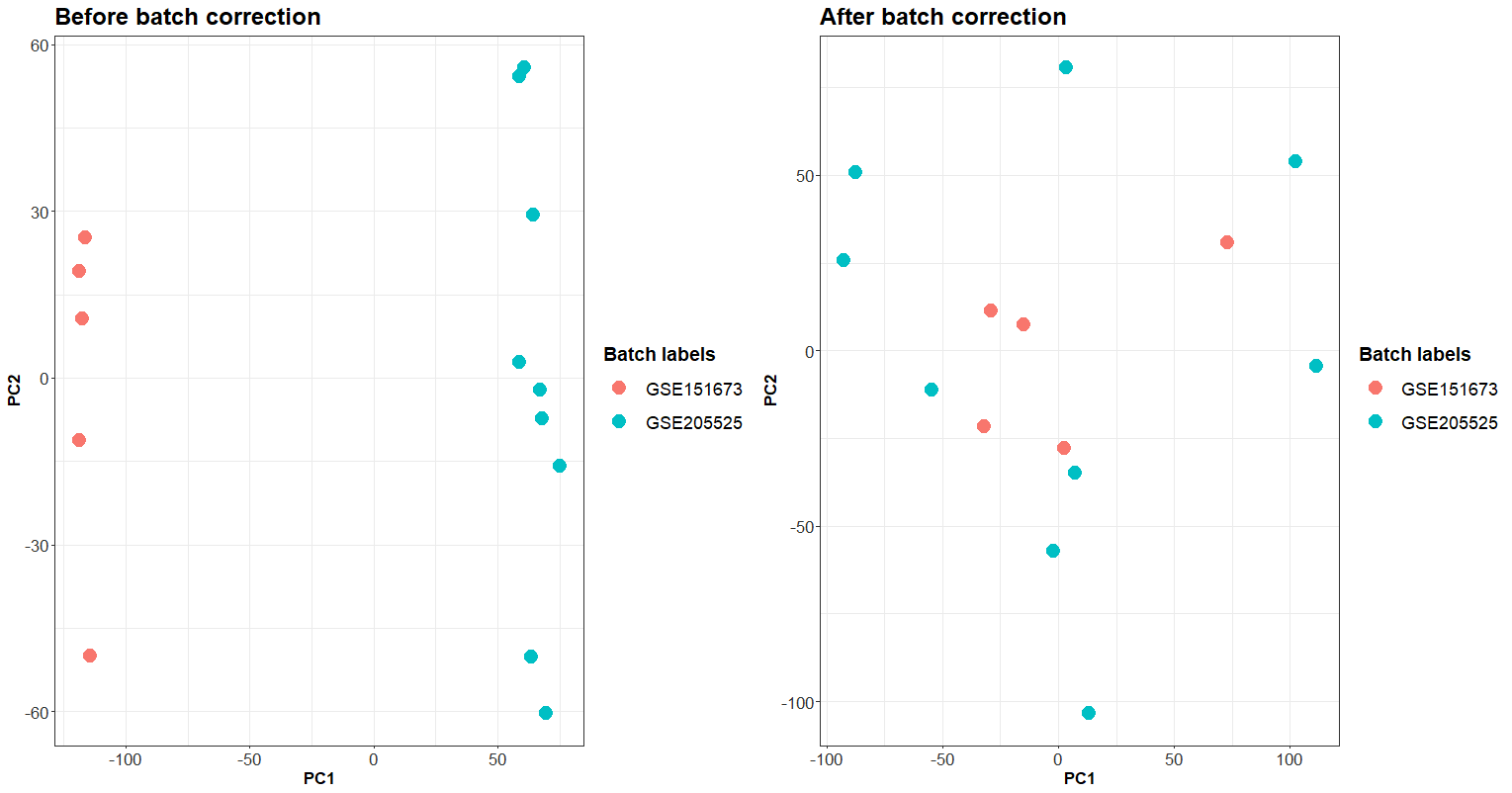

Supplement: Supplementary file 1 [file mmc1.zip › Supplementary_material/multi_studies_batch_adjust_pca_plots/epithelial_healthy_RNAseq.png]

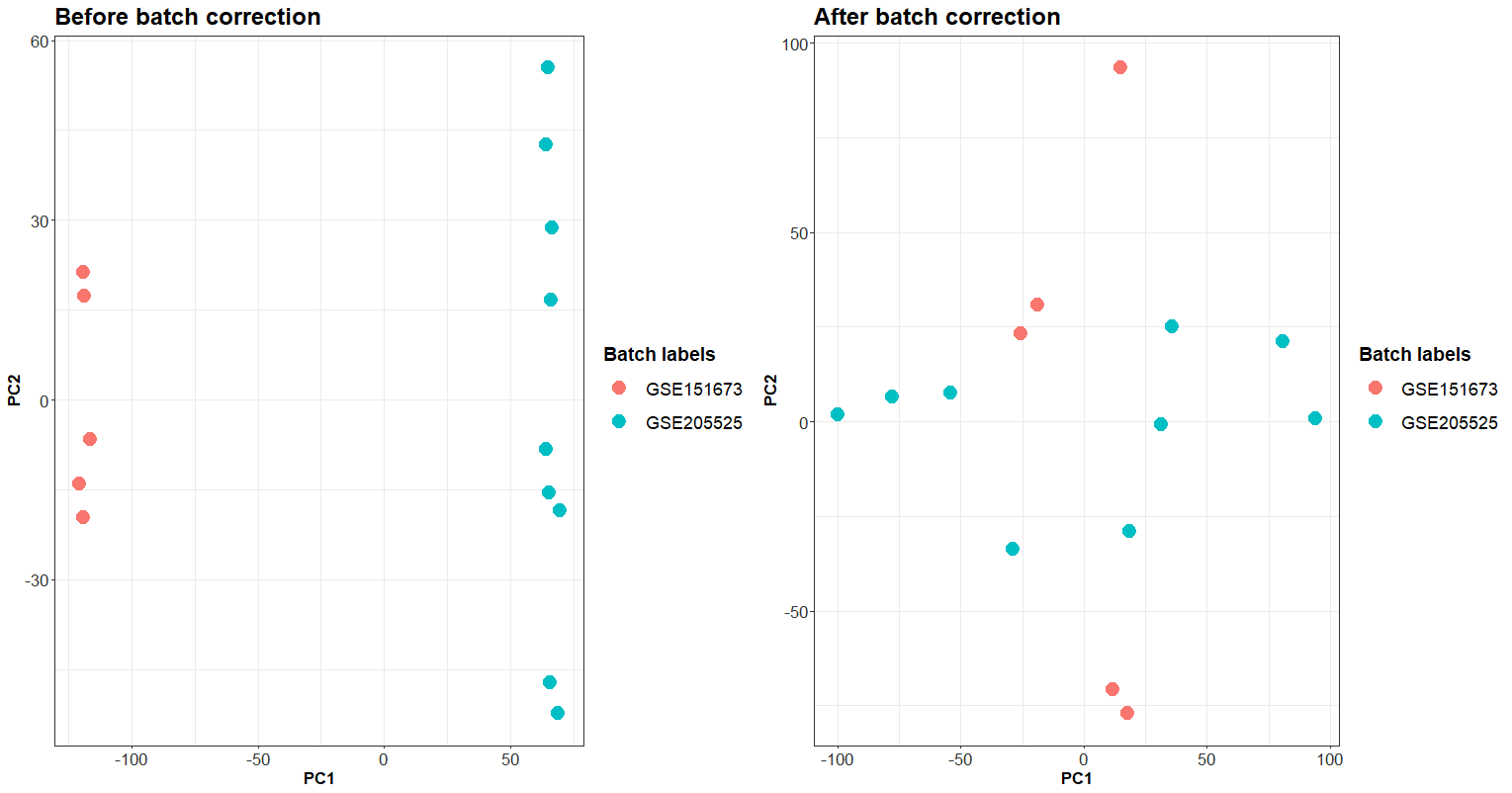

Supplement: Supplementary file 1 [file mmc1.zip › Supplementary_material/multi_studies_batch_adjust_pca_plots/epithelial_disease_RNAseq.png]

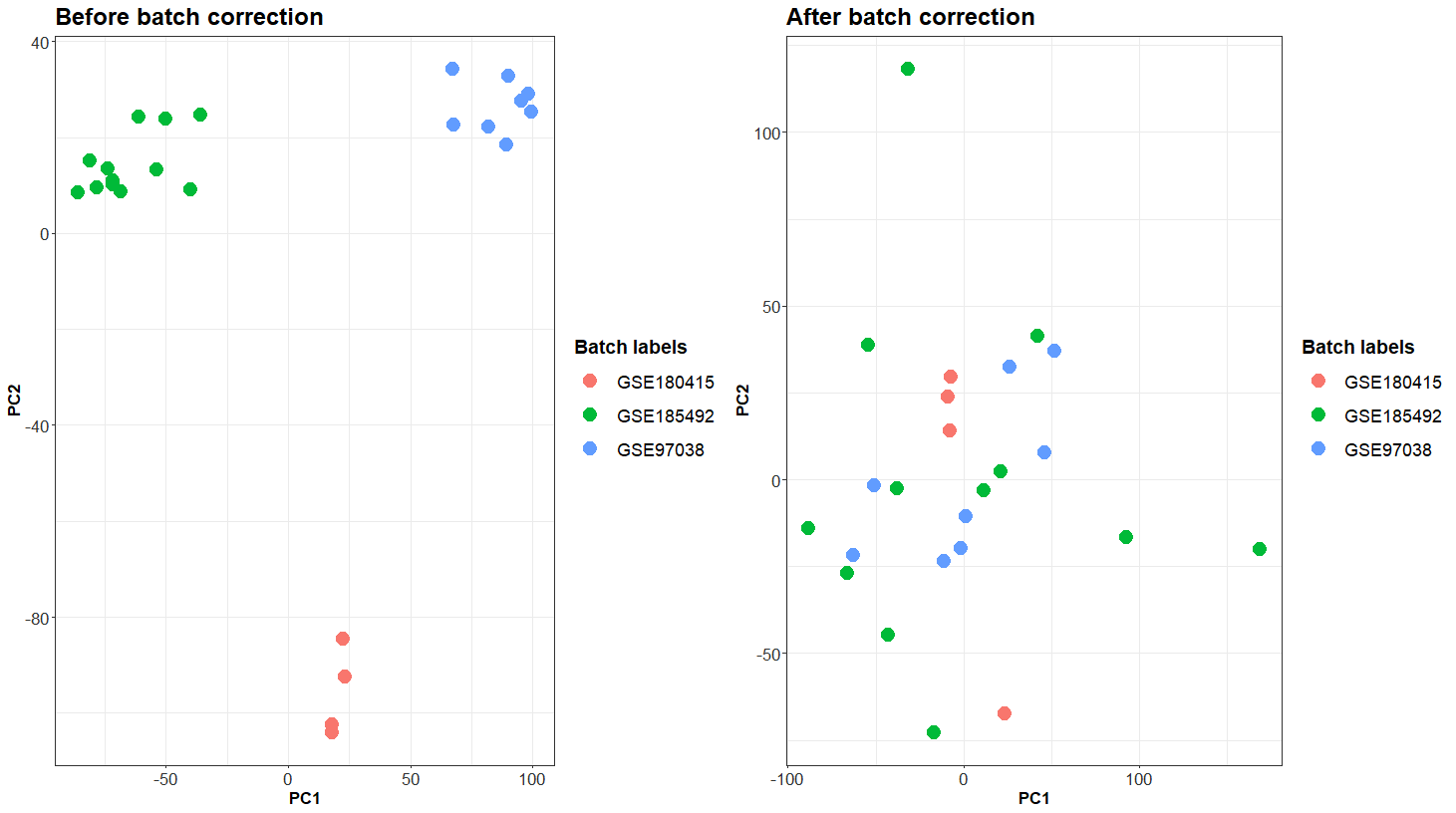

Supplement: Supplementary file 1 [file mmc1.zip › Supplementary_material/multi_studies_batch_adjust_pca_plots/fibroblast_healthy_RNAseq.png]

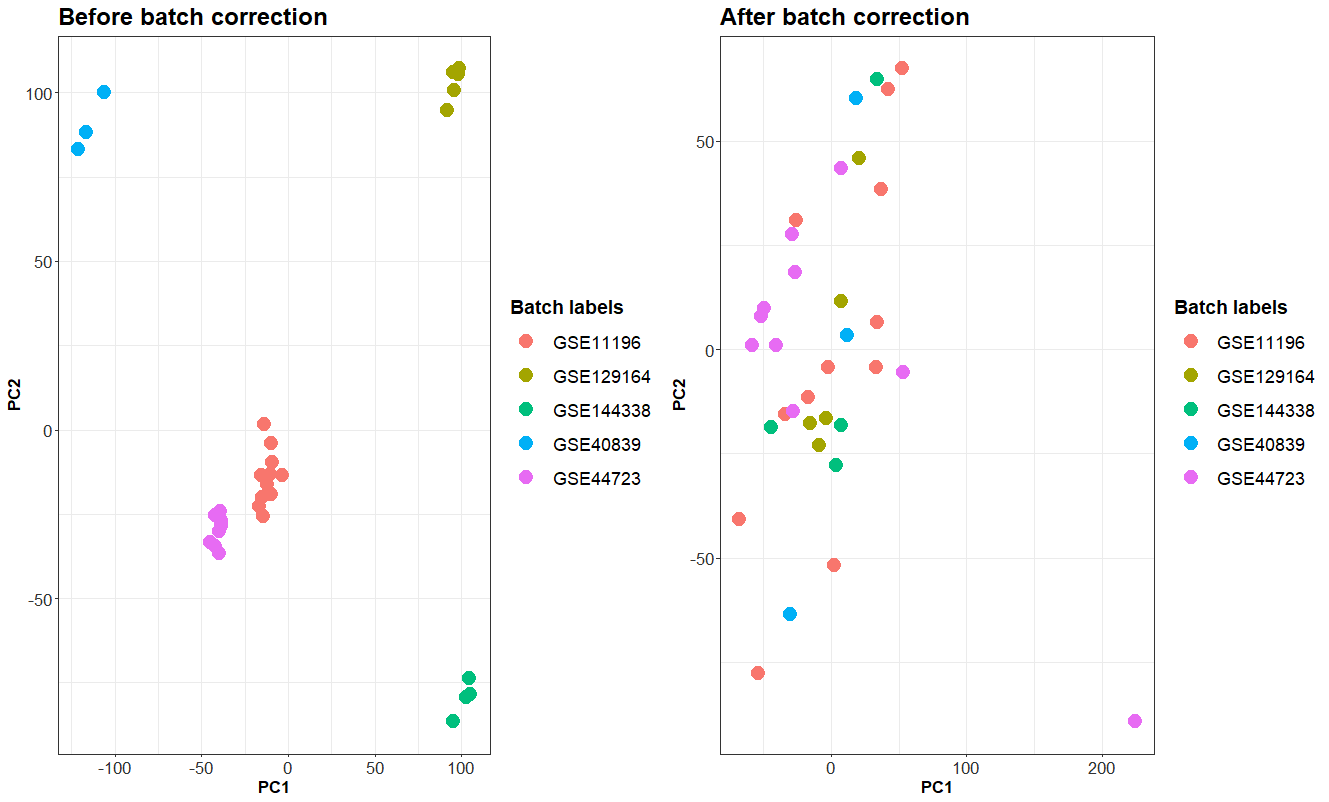

Supplement: Supplementary file 1 [file mmc1.zip › Supplementary_material/multi_studies_batch_adjust_pca_plots/fibroblast_disease_microarray.png]

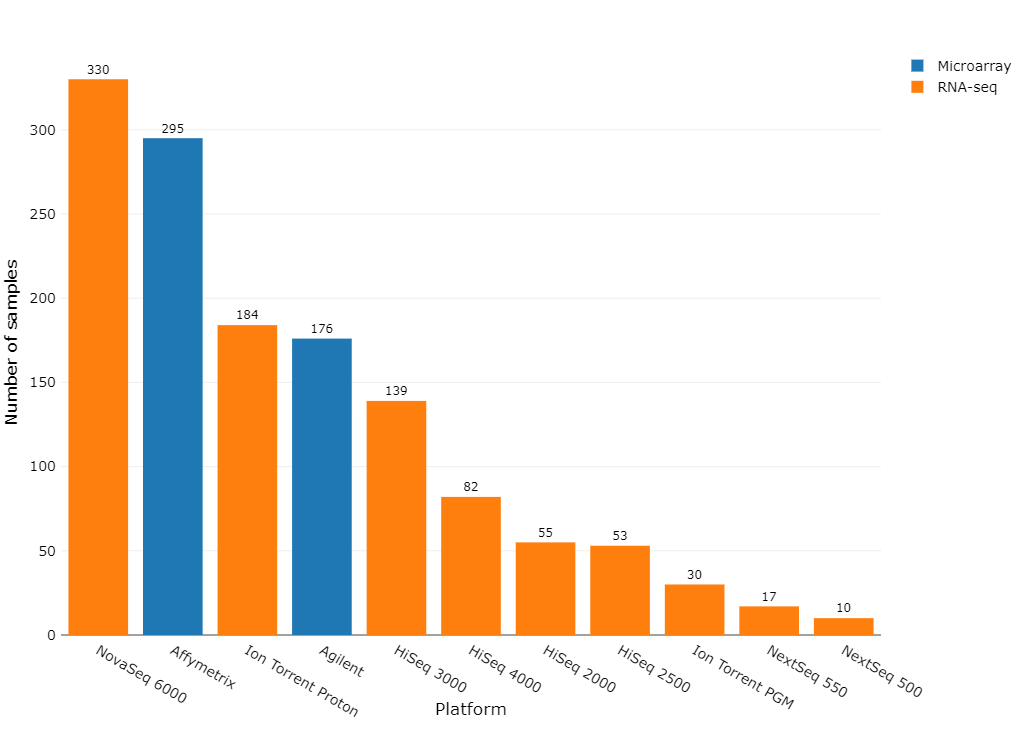

Supplement: Supplementary file 1 [file mmc1.zip › Supplementary_material/platform_plots/seq_type_samples.png]

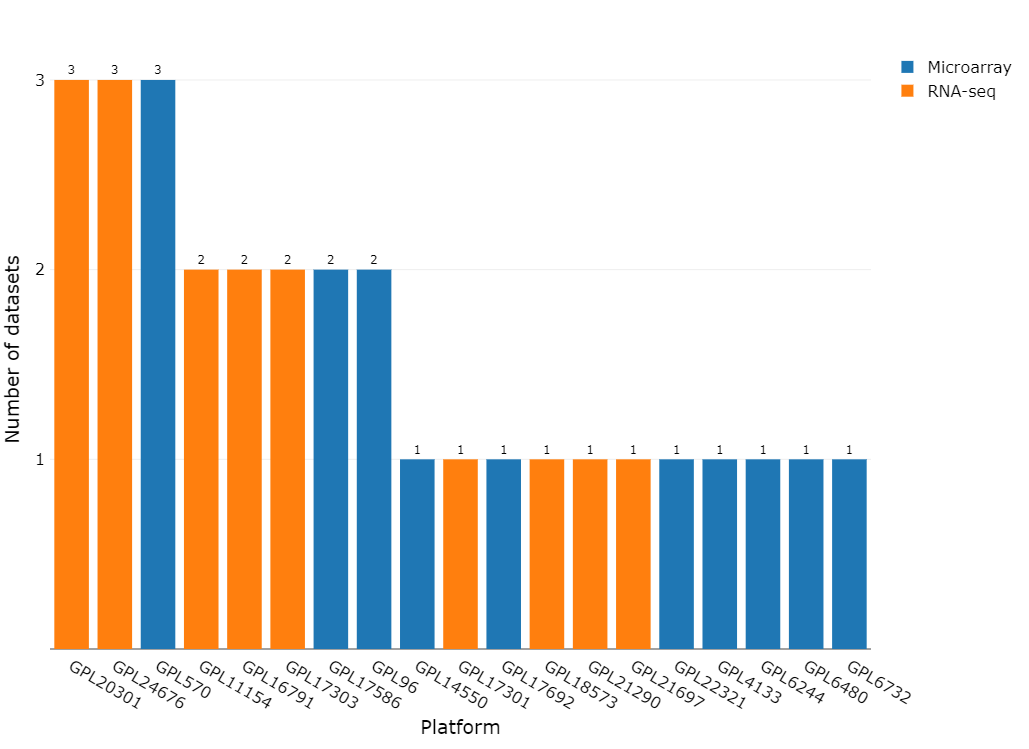

Supplement: Supplementary file 1 [file mmc1.zip › Supplementary_material/platform_plots/seq_tech.png]

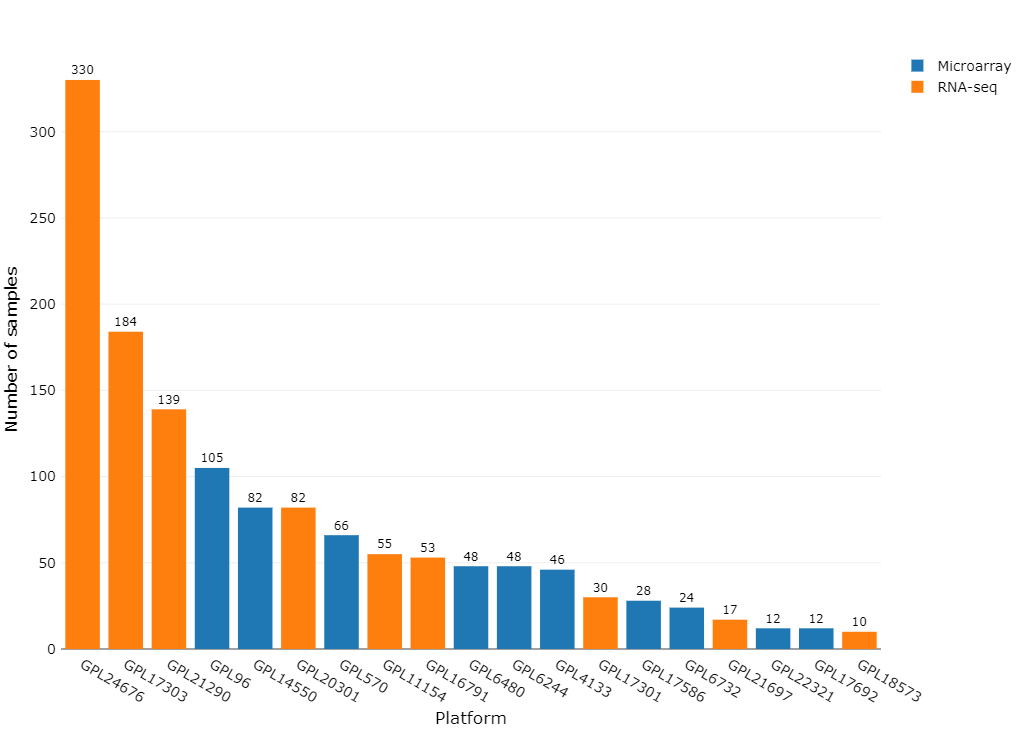

Supplement: Supplementary file 1 [file mmc1.zip › Supplementary_material/platform_plots/seq_tech_samples.png]

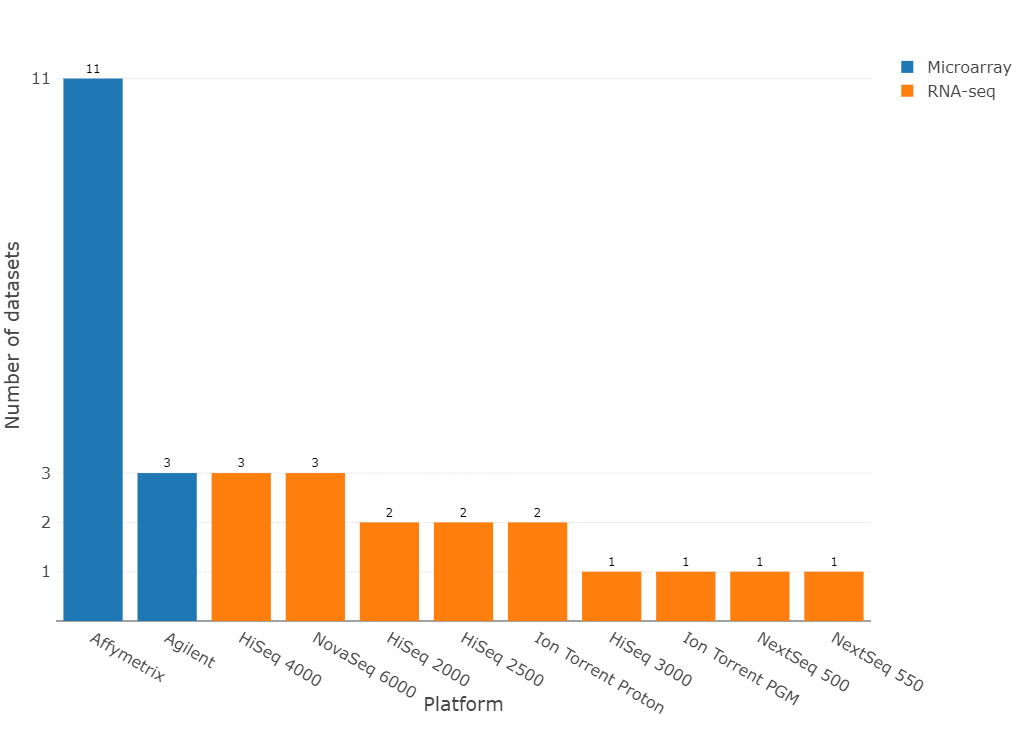

Supplement: Supplementary file 1 [file mmc1.zip › Supplementary_material/platform_plots/seq_type.png]

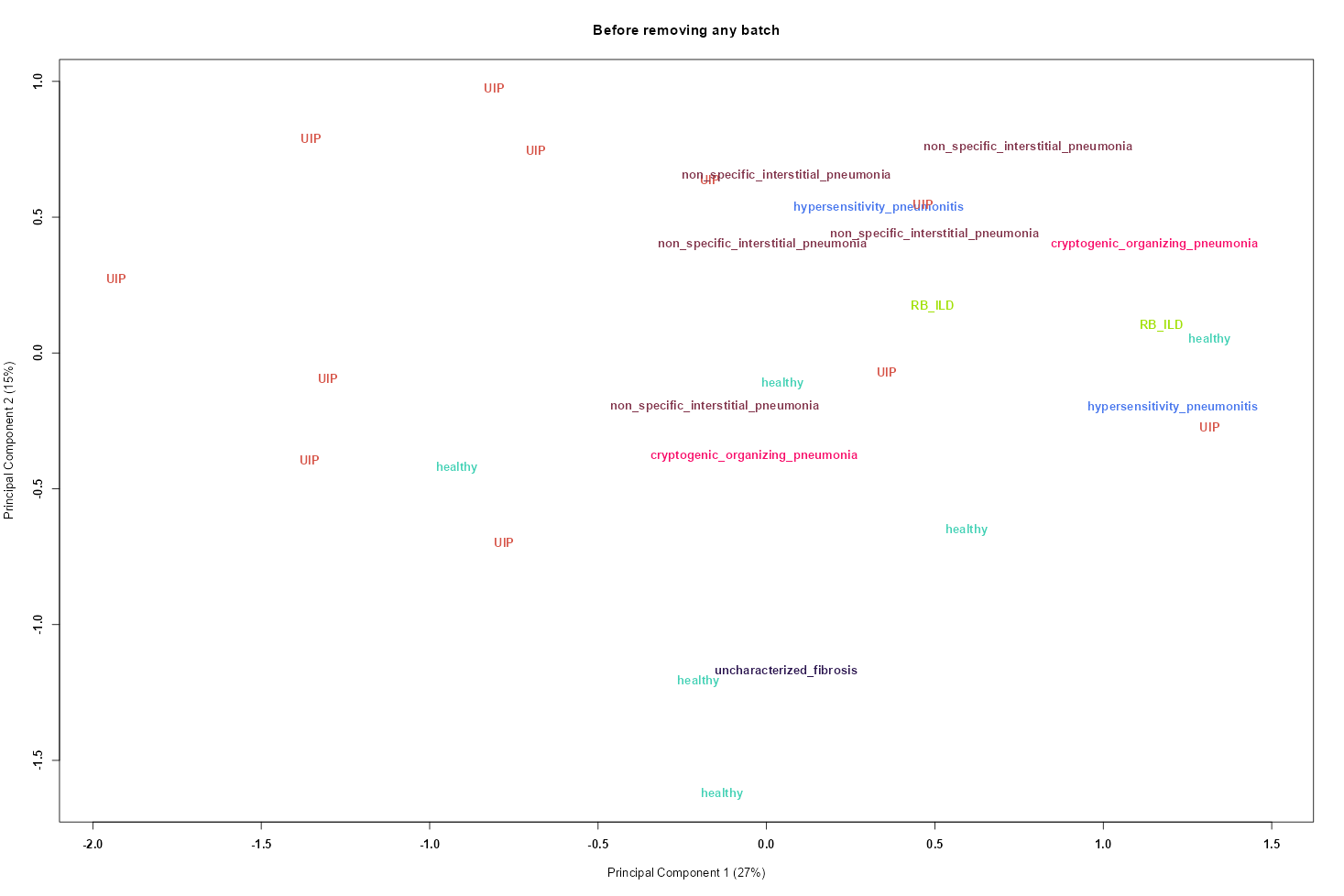

Supplement: Supplementary file 1 [file mmc1.zip › Supplementary_material/DNA-microarray/GSE21369/GSE21369_MDS_before_correction.png]

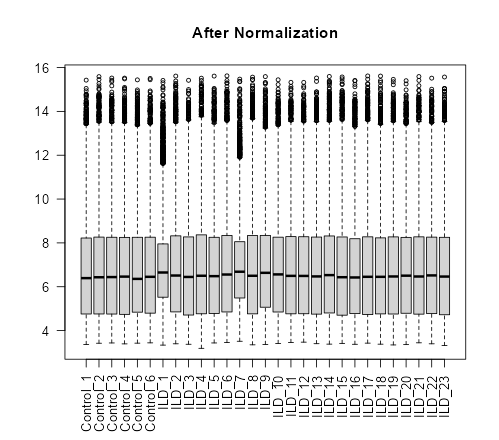

Supplement: Supplementary file 1 [file mmc1.zip › Supplementary_material/DNA-microarray/GSE21369/GSE21369_boxplot_after_normalization.png]

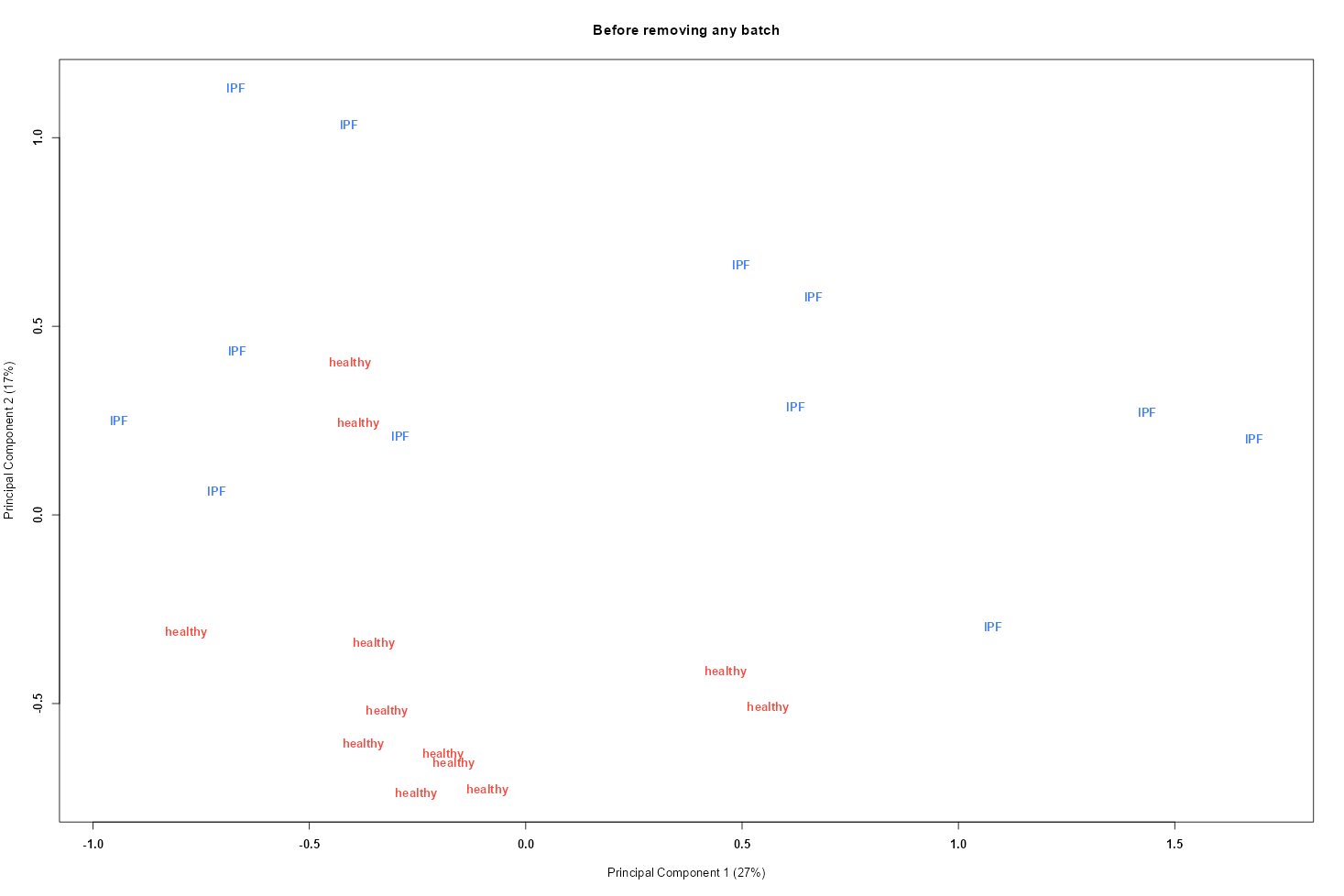

Supplement: Supplementary file 1 [file mmc1.zip › Supplementary_material/DNA-microarray/GSE11196/GSE11196_MDS_before_correction_total_RNA.png]

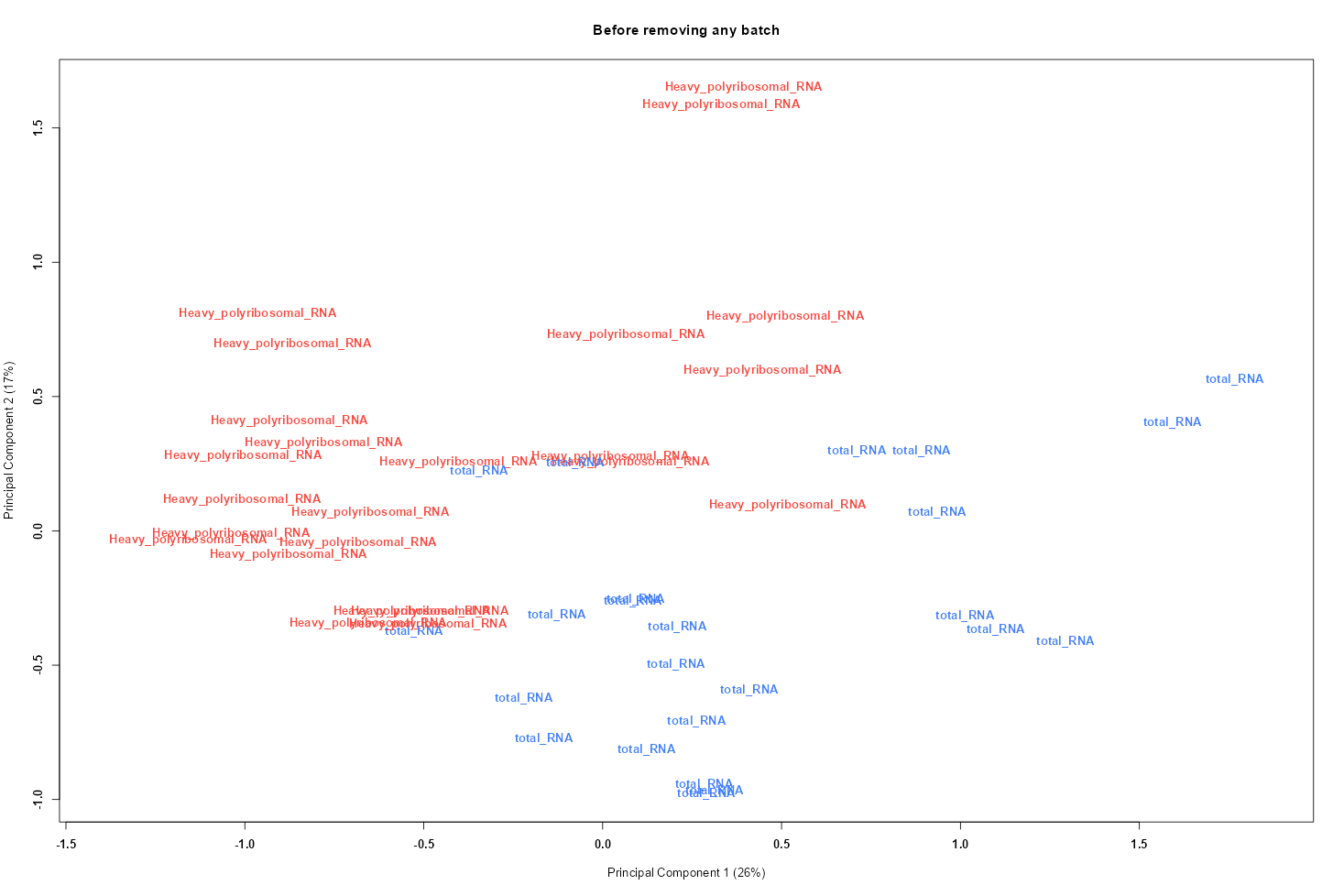

Supplement: Supplementary file 1 [file mmc1.zip › Supplementary_material/DNA-microarray/GSE11196/GSE11196_MDS_before_correction_all_samples.png]

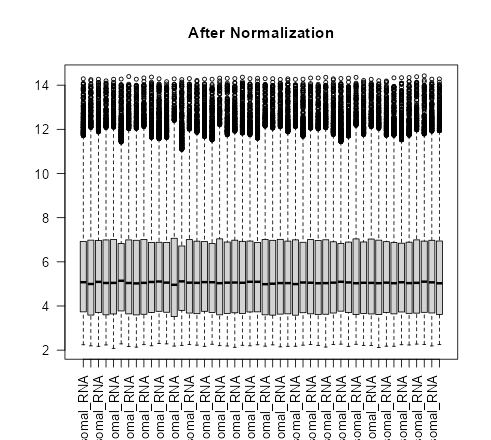

Supplement: Supplementary file 1 [file mmc1.zip › Supplementary_material/DNA-microarray/GSE11196/GSE11196_boxplot_after_normalization_all_samples_RNA.png]

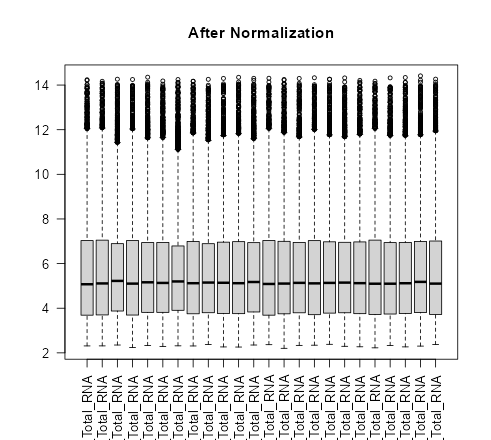

Supplement: Supplementary file 1 [file mmc1.zip › Supplementary_material/DNA-microarray/GSE11196/GSE11196_boxplot_after_normalization_total_RNA.png]

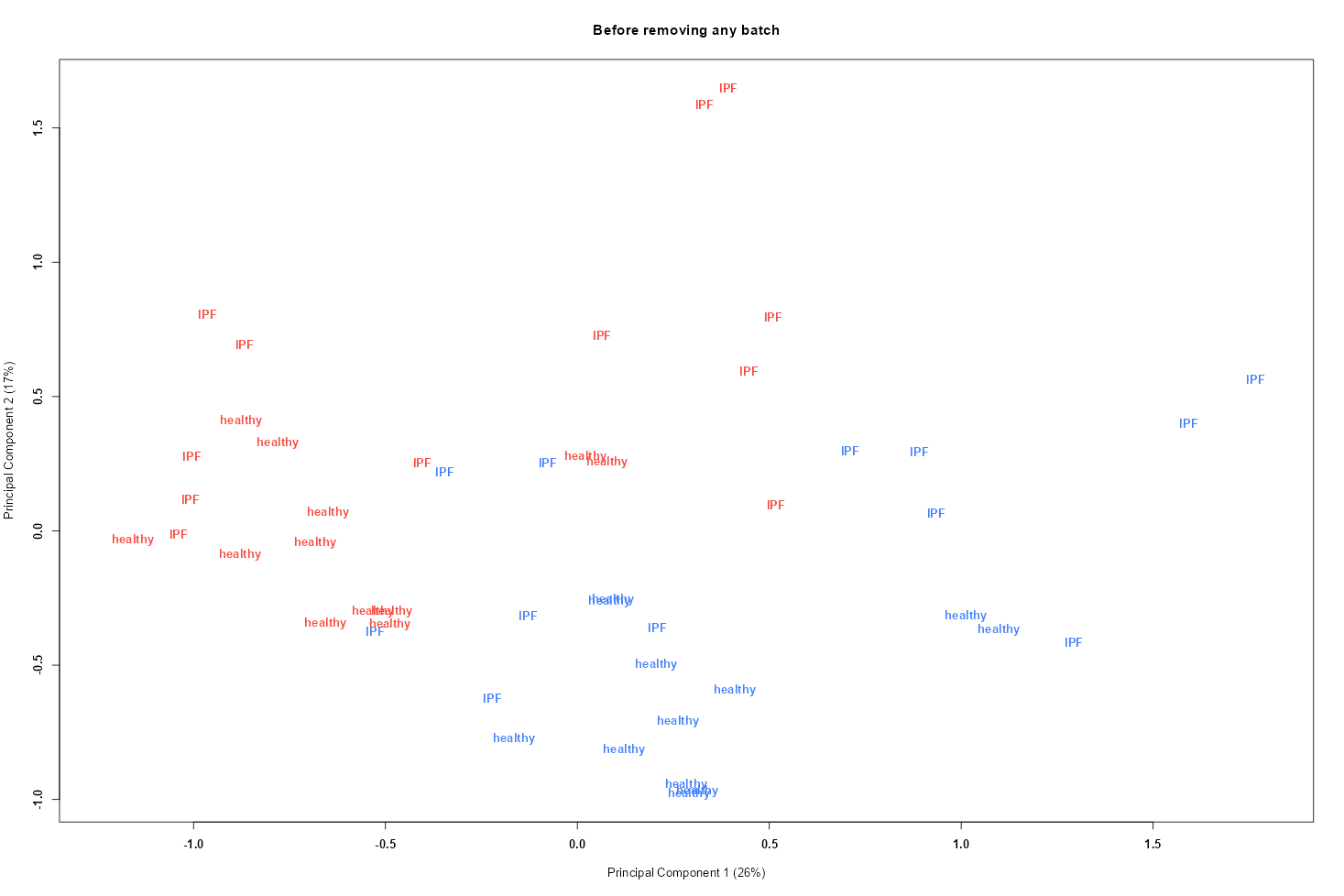

Supplement: Supplementary file 1 [file mmc1.zip › Supplementary_material/DNA-microarray/GSE11196/GSE11196_MDS_before_correction_all_samples_disease.png]

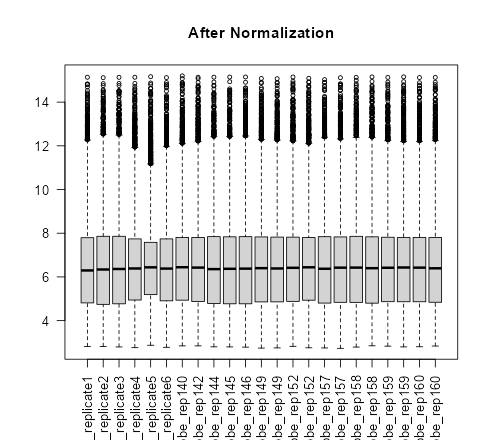

Supplement: Supplementary file 1 [file mmc1.zip › Supplementary_material/DNA-microarray/GSE24206/GSE24206_boxplot_after_normalization.png]

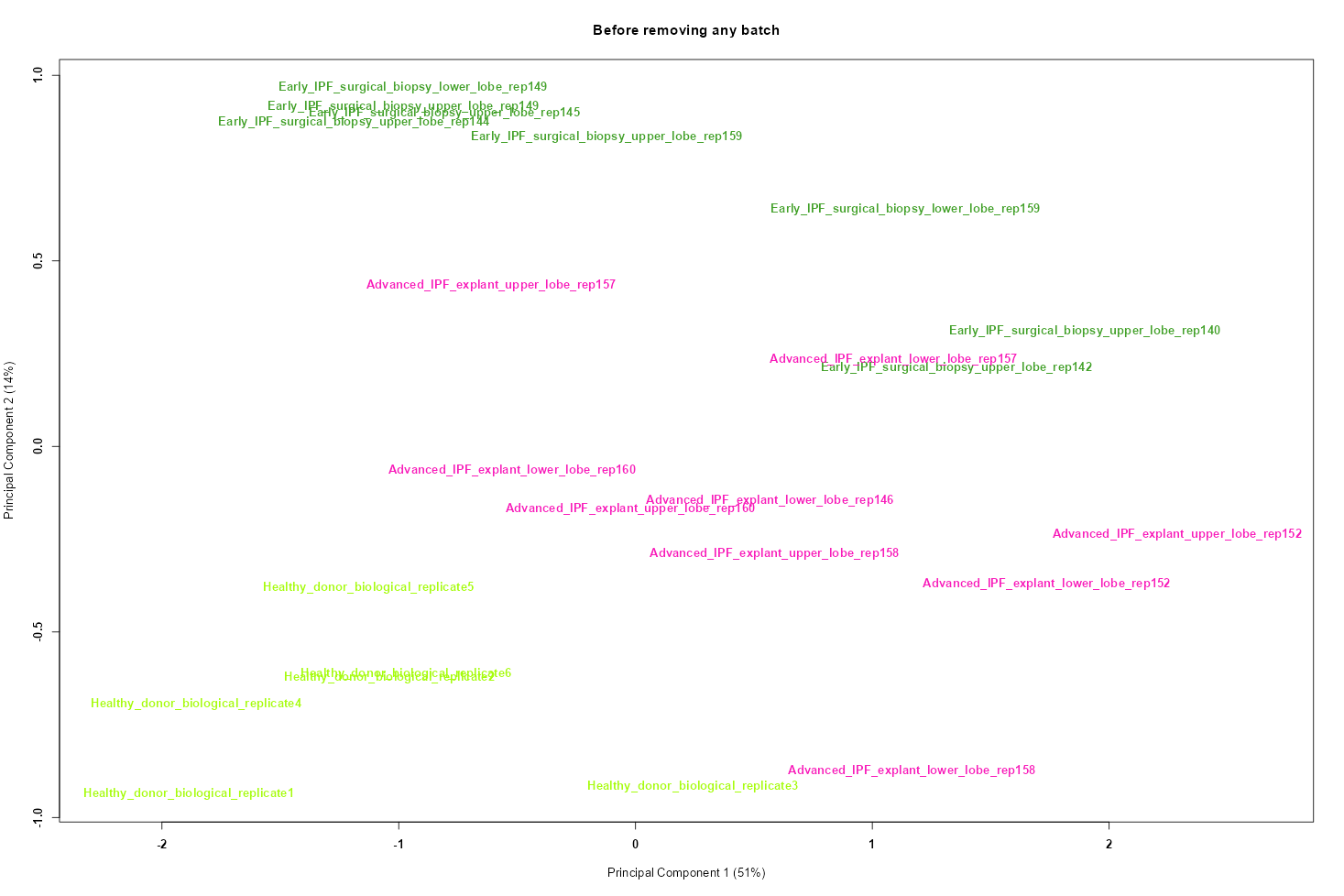

Supplement: Supplementary file 1 [file mmc1.zip › Supplementary_material/DNA-microarray/GSE24206/GSE24206_MDS_before_correction_disease_state.png]

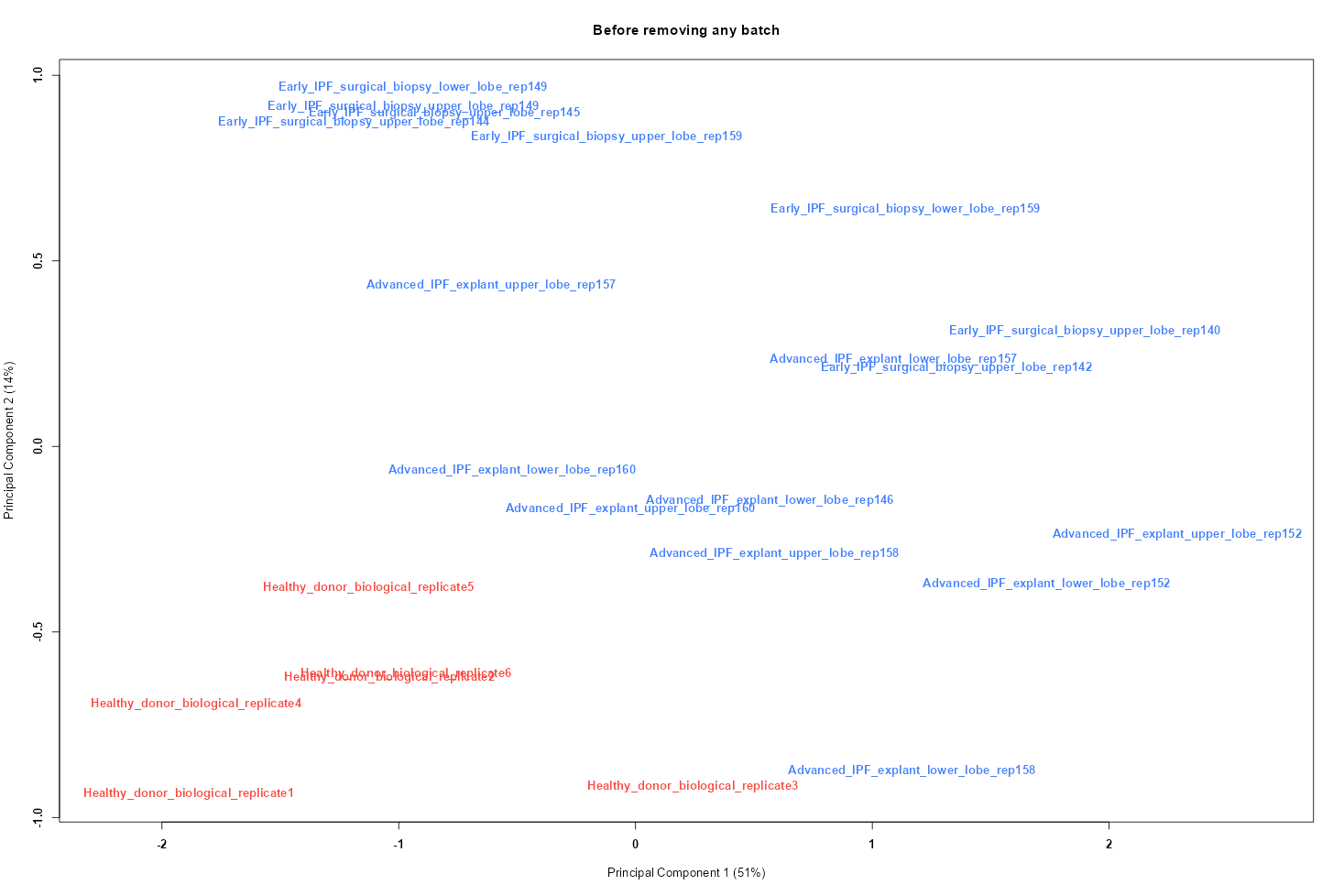

Supplement: Supplementary file 1 [file mmc1.zip › Supplementary_material/DNA-microarray/GSE24206/GSE24206_MDS_before_correction_disease.png]

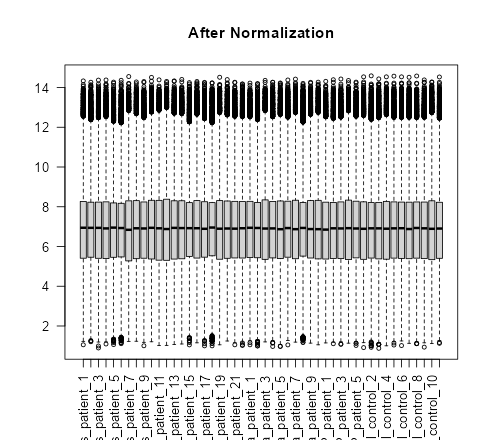

Supplement: Supplementary file 1 [file mmc1.zip › Supplementary_material/DNA-microarray/GSE110147/GSE110147_boxplot_after_normalization.png]

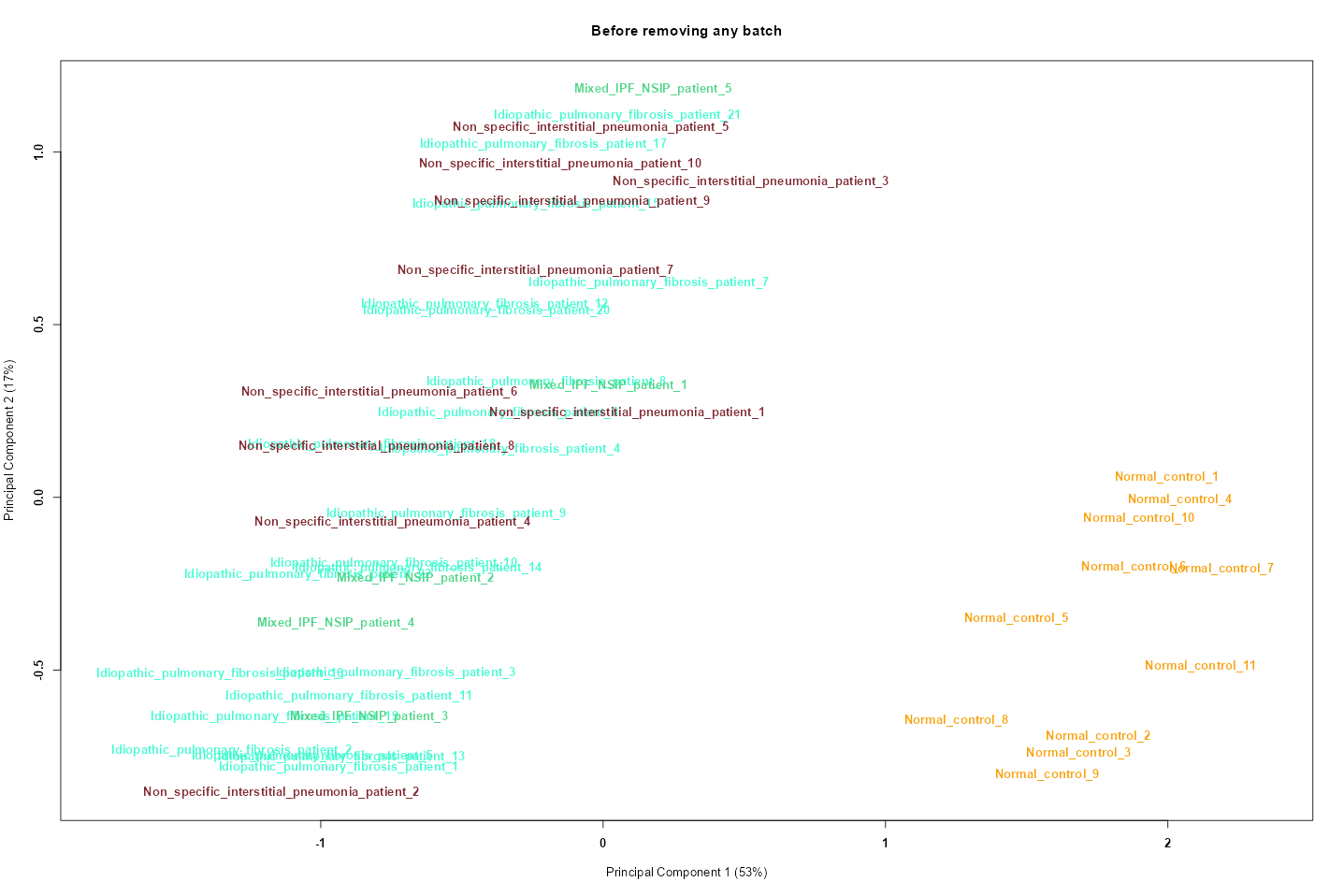

Supplement: Supplementary file 1 [file mmc1.zip › Supplementary_material/DNA-microarray/GSE110147/GSE110147_MDS_before_correction.png]

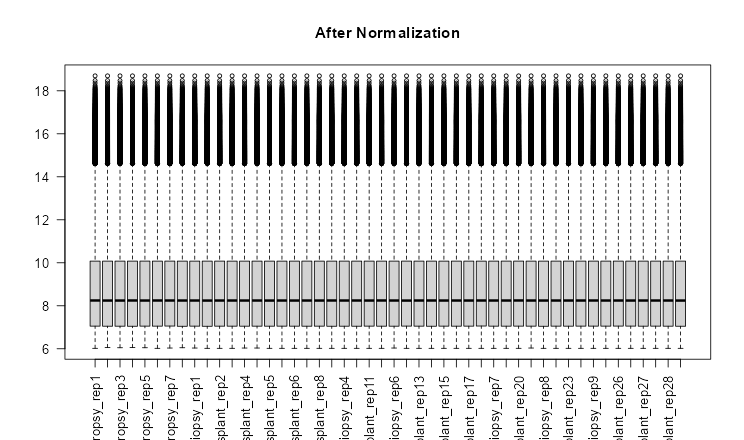

Supplement: Supplementary file 1 [file mmc1.zip › Supplementary_material/DNA-microarray/GSE53845/GSE53845_boxplot_after_normalization.png]

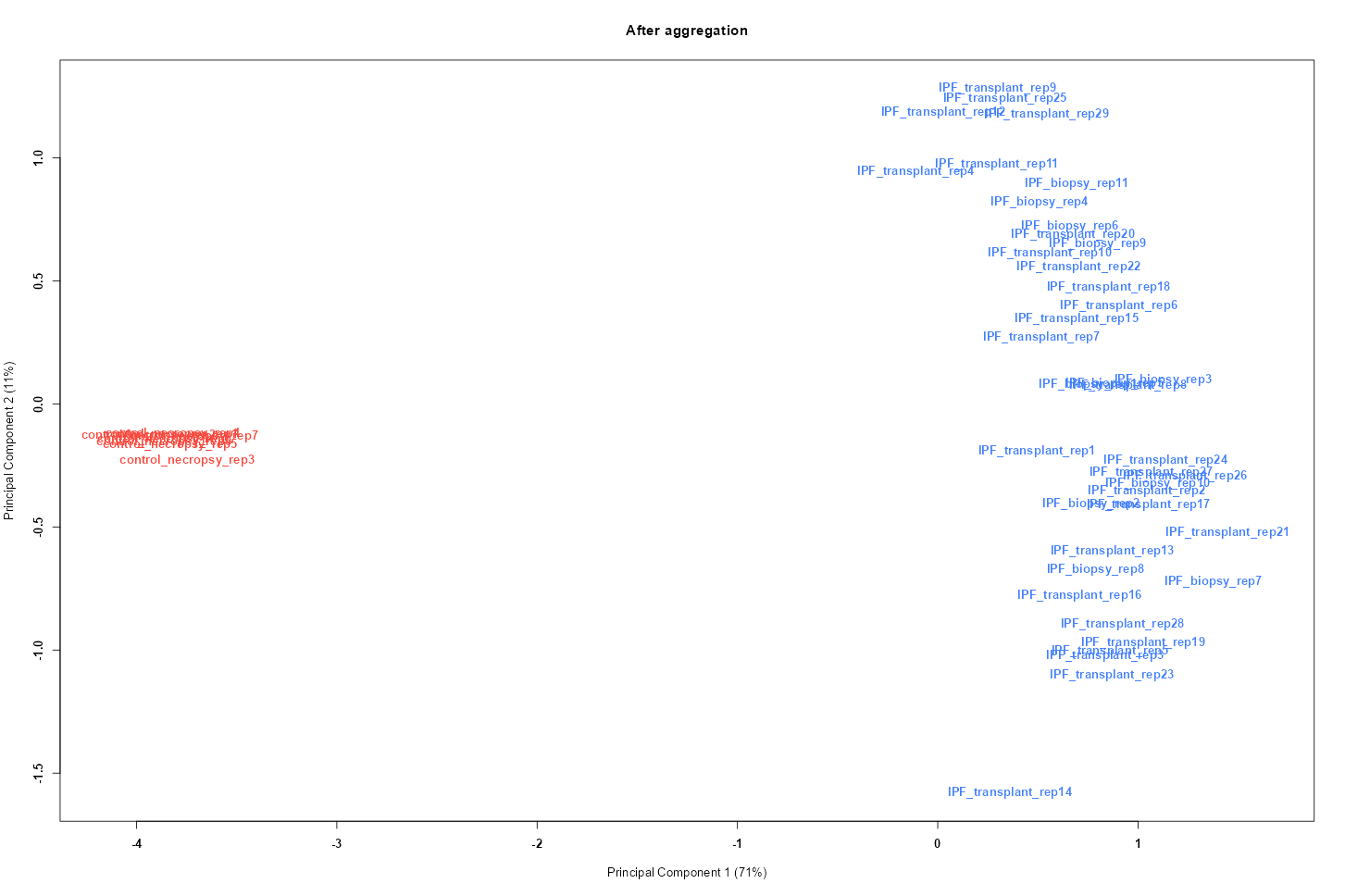

Supplement: Supplementary file 1 [file mmc1.zip › Supplementary_material/DNA-microarray/GSE53845/GSE53845_MDS_after_aggregation.png]

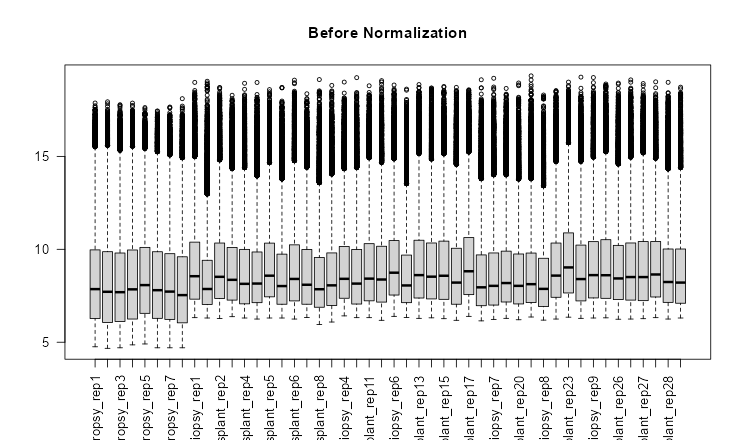

Supplement: Supplementary file 1 [file mmc1.zip › Supplementary_material/DNA-microarray/GSE53845/GSE53845_boxplot_before_normalization.png]

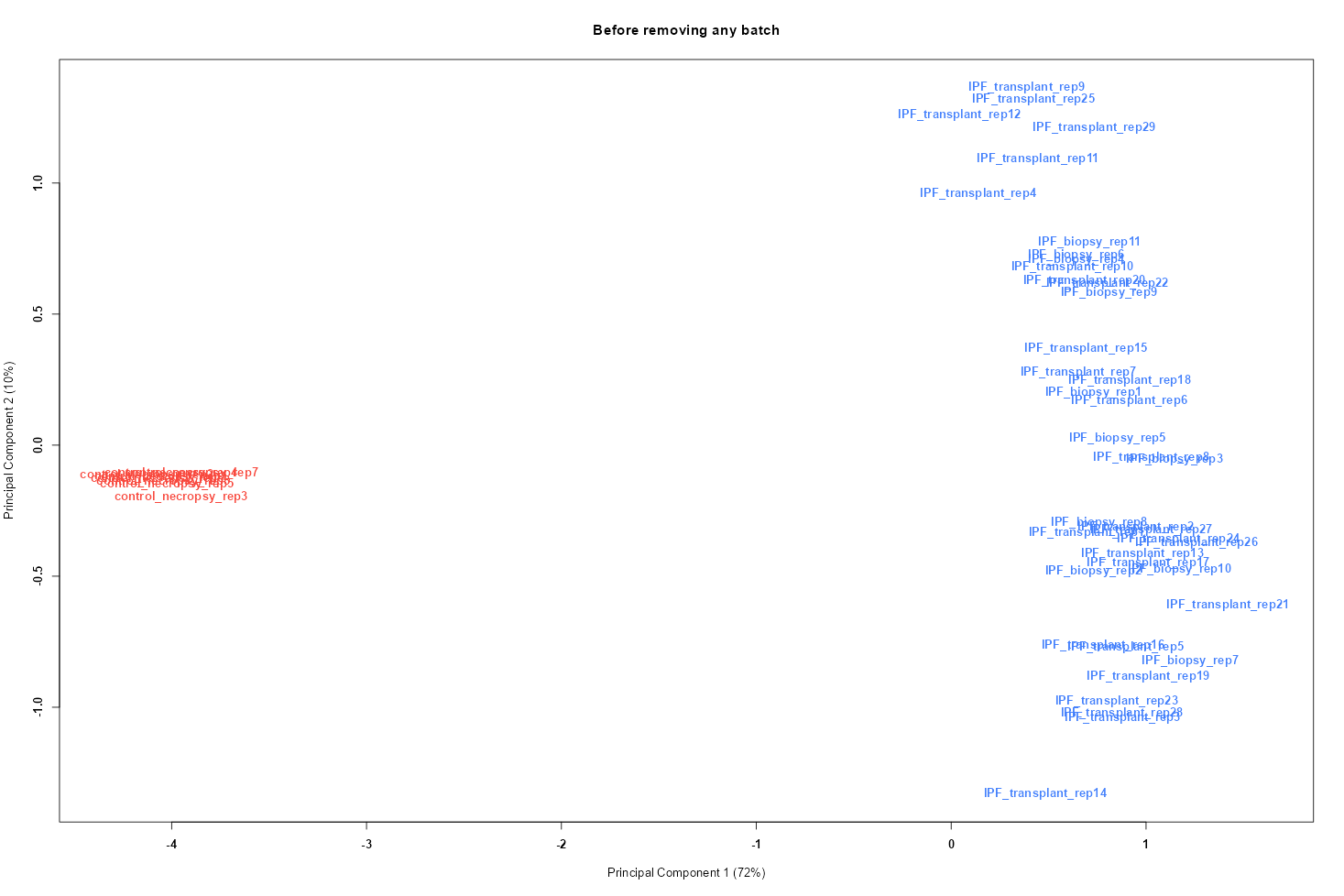

Supplement: Supplementary file 1 [file mmc1.zip › Supplementary_material/DNA-microarray/GSE53845/GSE53845_MDS_before_correction.png]

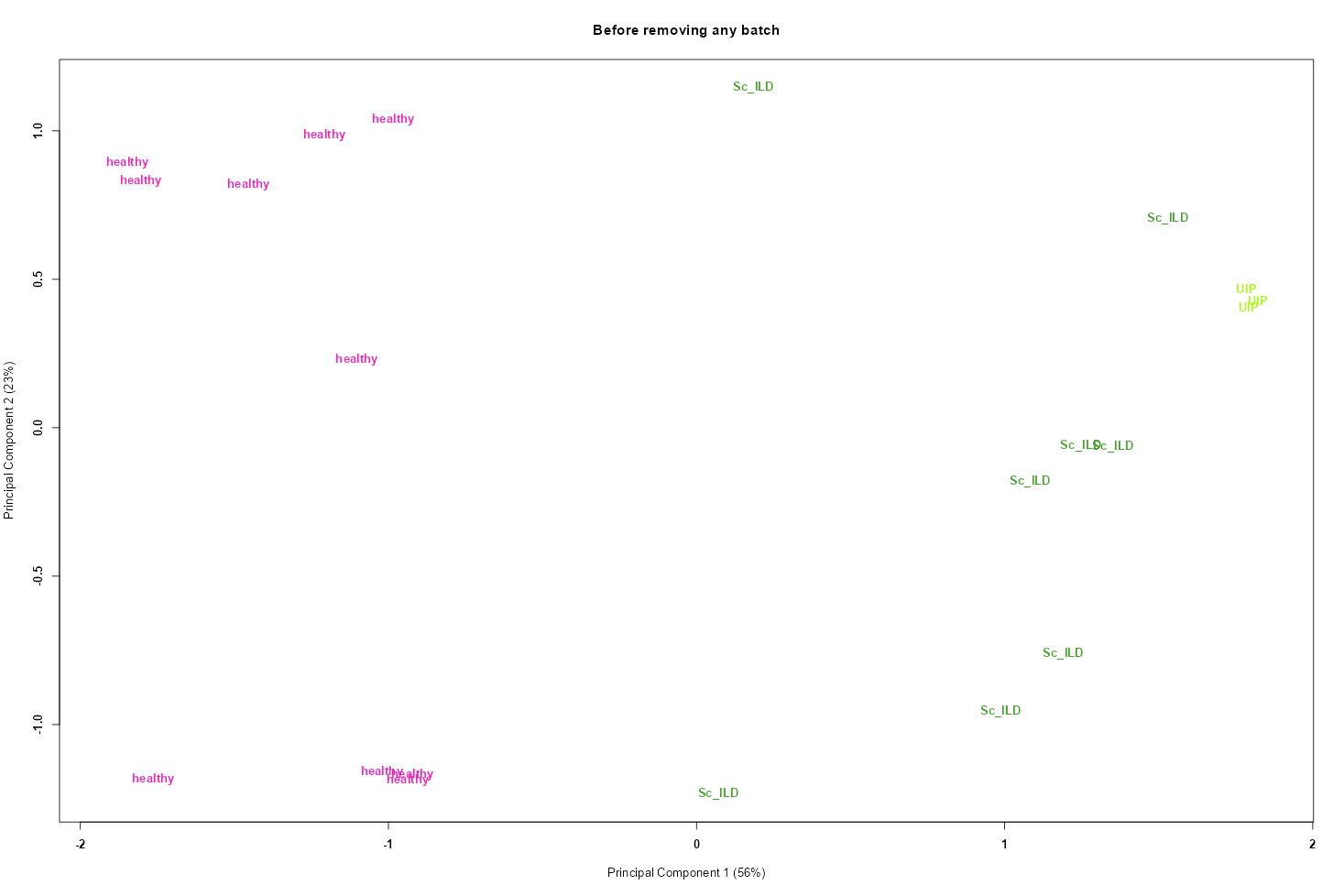

Supplement: Supplementary file 1 [file mmc1.zip › Supplementary_material/DNA-microarray/GSE40839/GSE40839_MDS_before_correction.png]

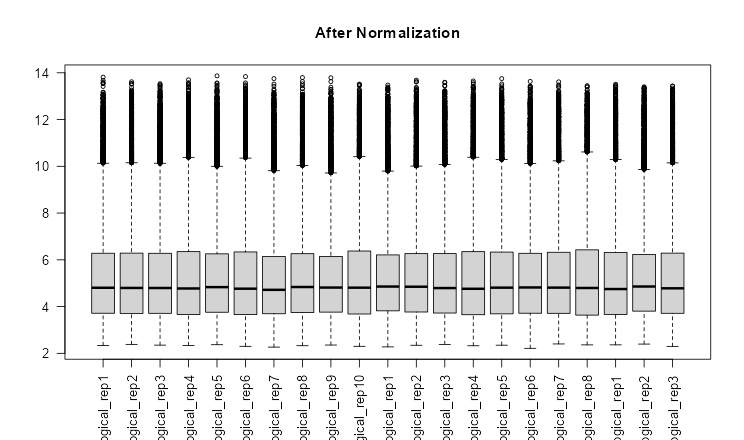

Supplement: Supplementary file 1 [file mmc1.zip › Supplementary_material/DNA-microarray/GSE40839/GSE40839_boxplot_after_normalization.png]

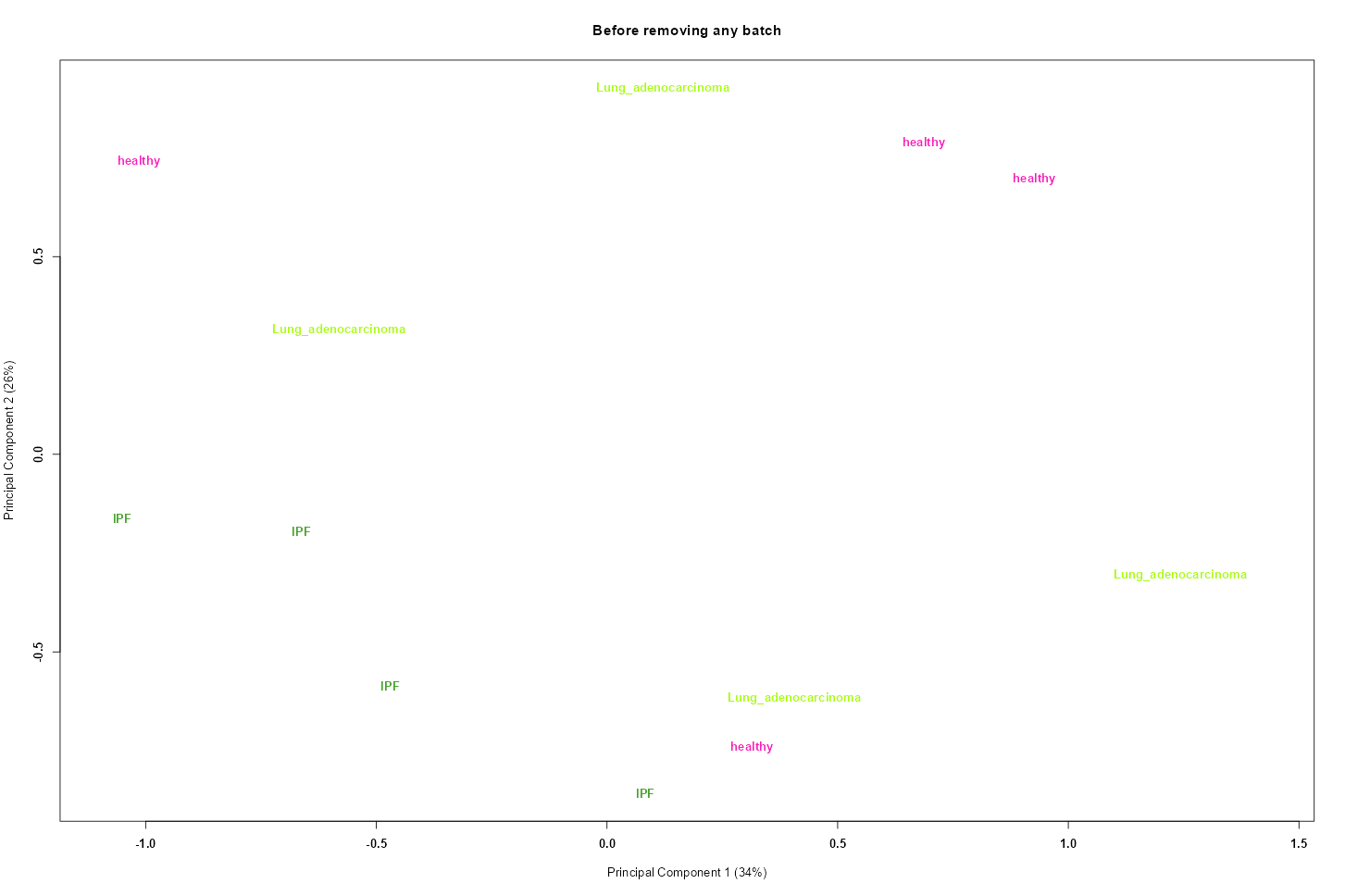

Supplement: Supplementary file 1 [file mmc1.zip › Supplementary_material/DNA-microarray/GSE144338/GSE144338_MDS_before_correction.png]

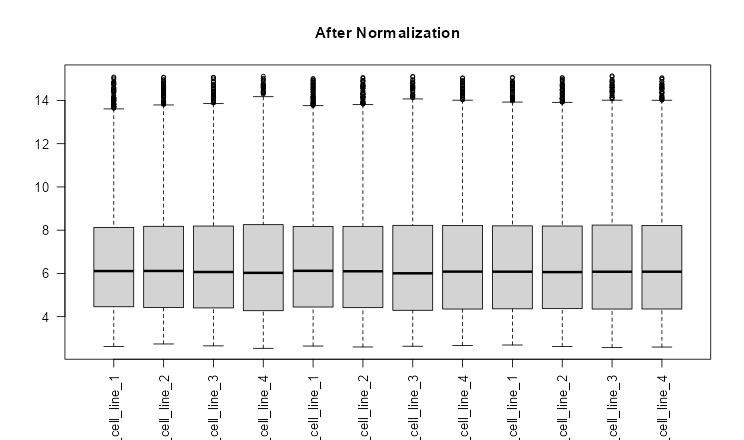

Supplement: Supplementary file 1 [file mmc1.zip › Supplementary_material/DNA-microarray/GSE144338/GSE144338_boxplot_after_normalization.png]

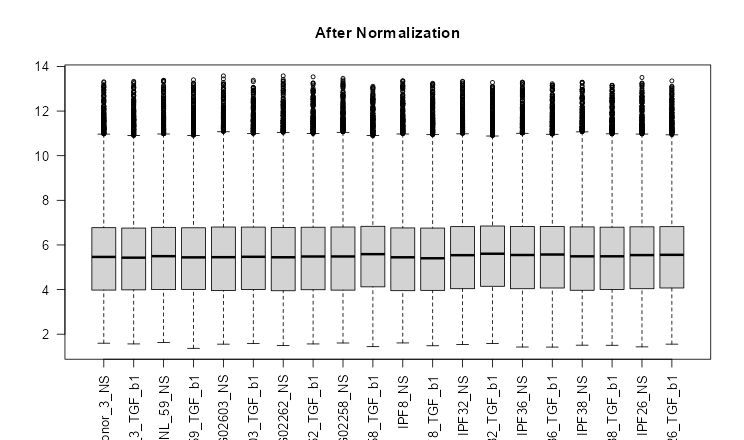

Supplement: Supplementary file 1 [file mmc1.zip › Supplementary_material/DNA-microarray/GSE129164/GSE129164_boxplot_after_normalization.png]

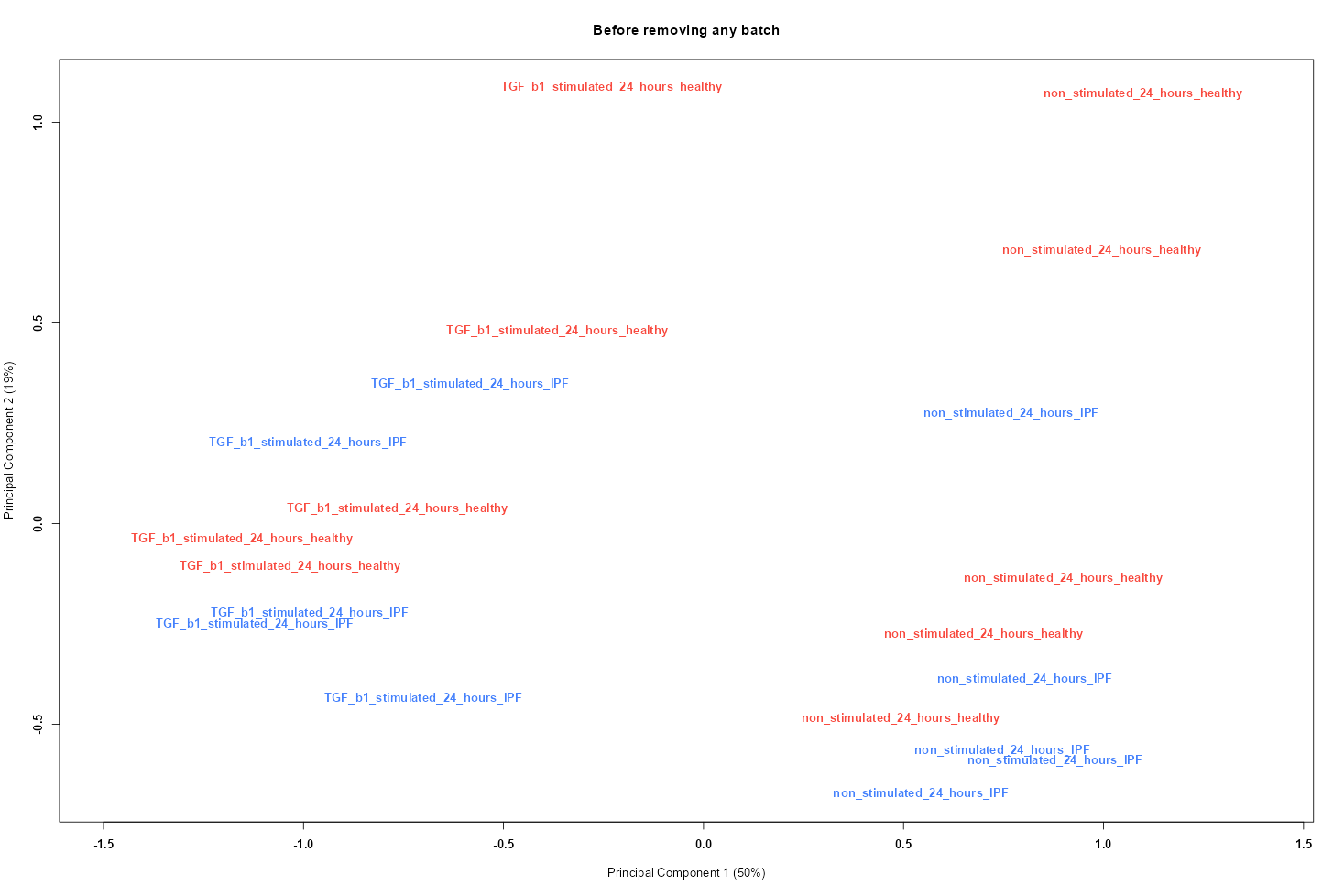

Supplement: Supplementary file 1 [file mmc1.zip › Supplementary_material/DNA-microarray/GSE129164/GSE129164_MDS_before_correction_treatment.png]

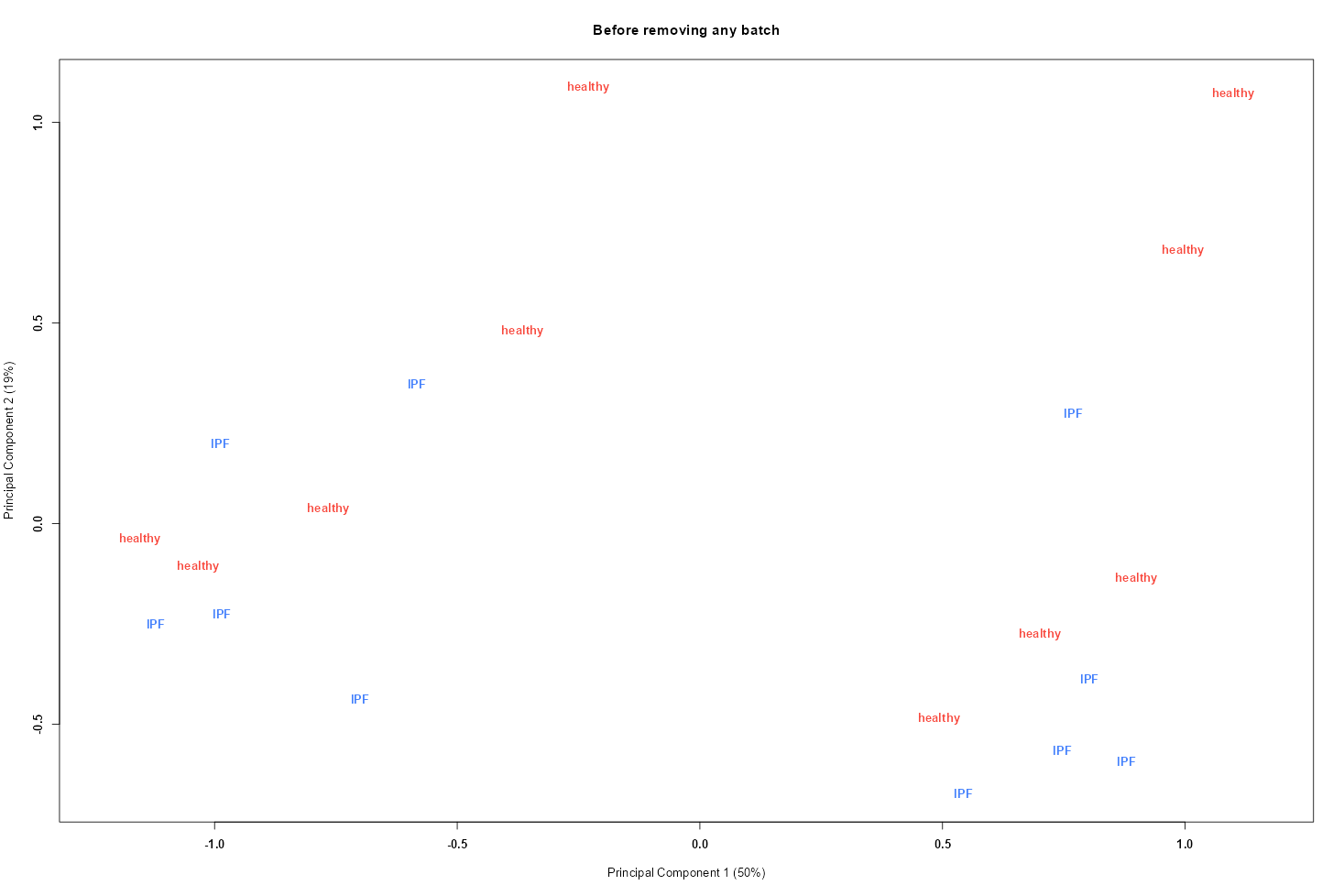

Supplement: Supplementary file 1 [file mmc1.zip › Supplementary_material/DNA-microarray/GSE129164/GSE129164_MDS_before_correction.png]

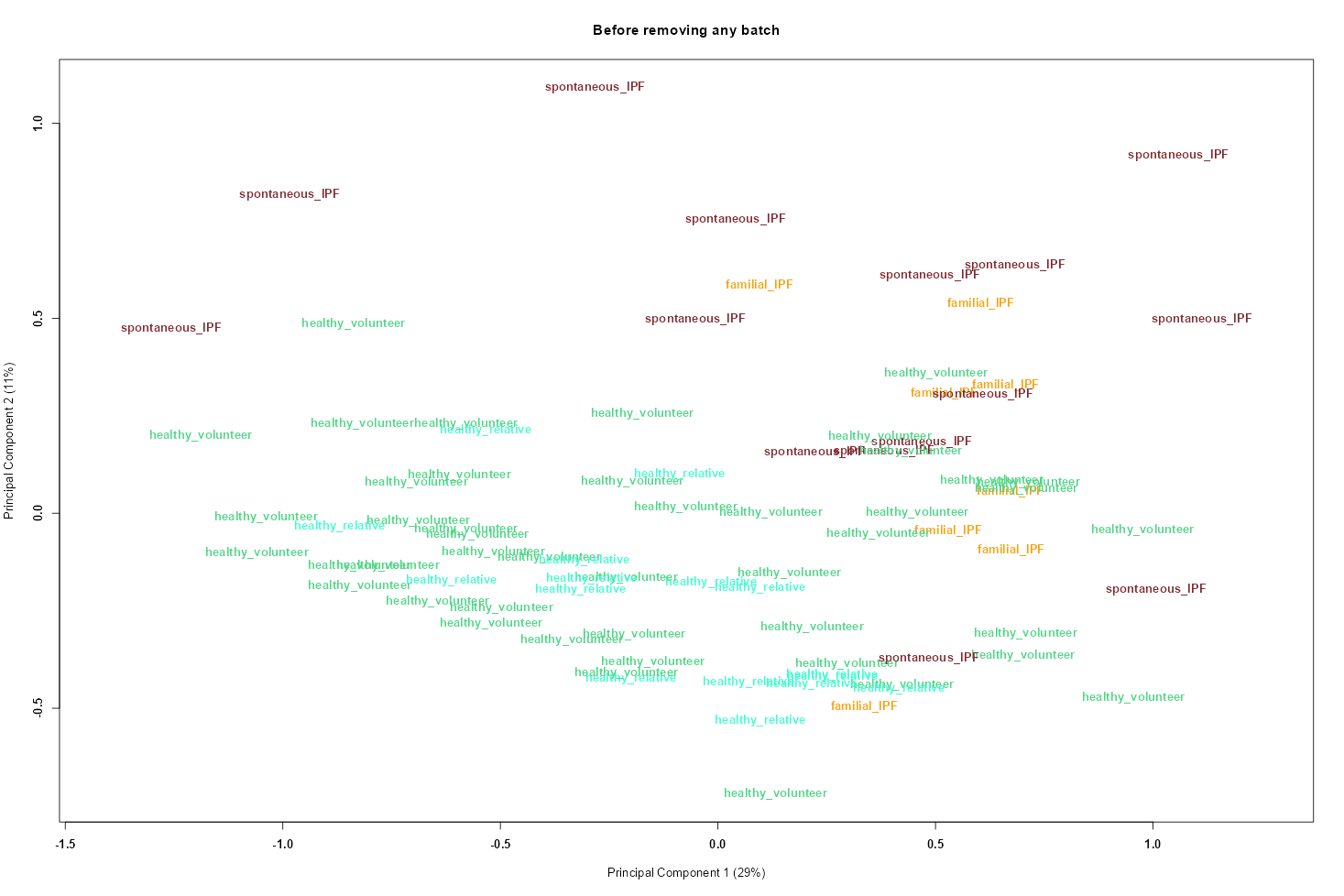

Supplement: Supplementary file 1 [file mmc1.zip › Supplementary_material/DNA-microarray/GSE49072/GSE49072_MDS_before_correction.png]

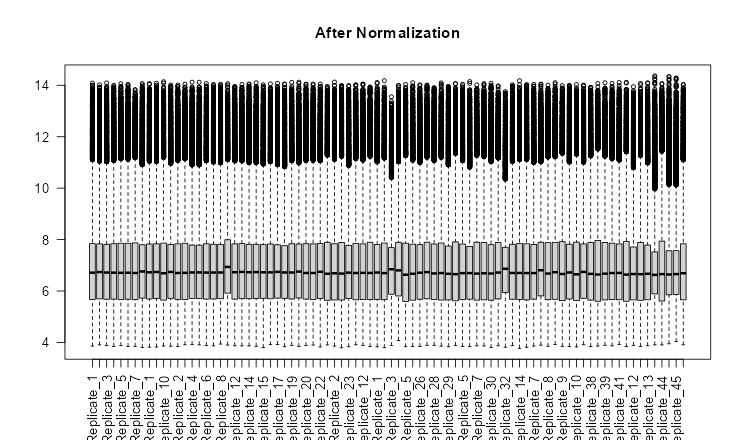

Supplement: Supplementary file 1 [file mmc1.zip › Supplementary_material/DNA-microarray/GSE49072/GSE49072_boxplot_after_normalization.png]

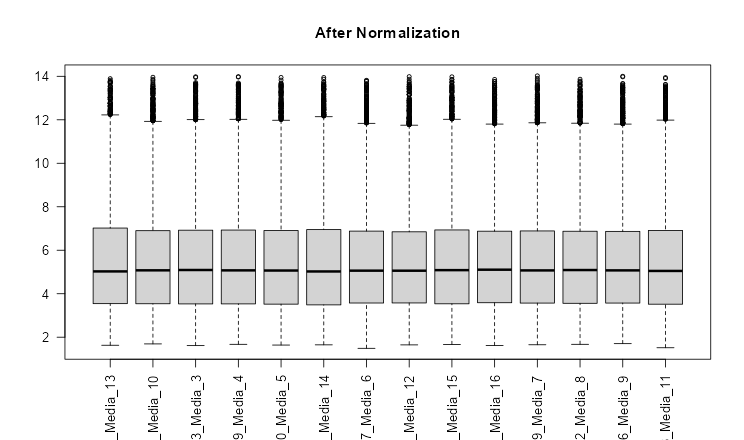

Supplement: Supplementary file 1 [file mmc1.zip › Supplementary_material/DNA-microarray/GSE44723/GSE44723_boxplot_after_normalization.png]

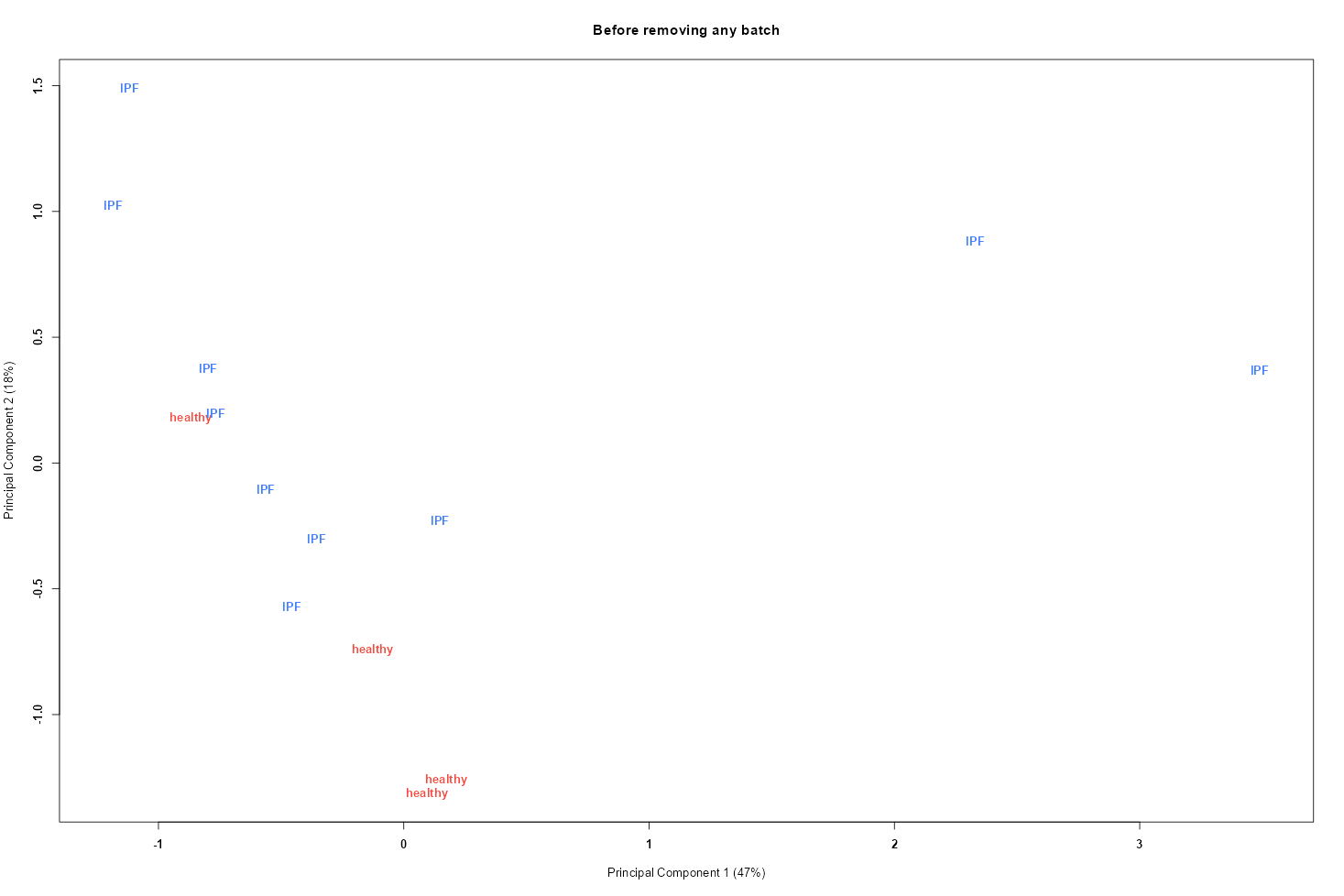

Supplement: Supplementary file 1 [file mmc1.zip › Supplementary_material/DNA-microarray/GSE44723/GSE44723_MDS_before_correction.png]

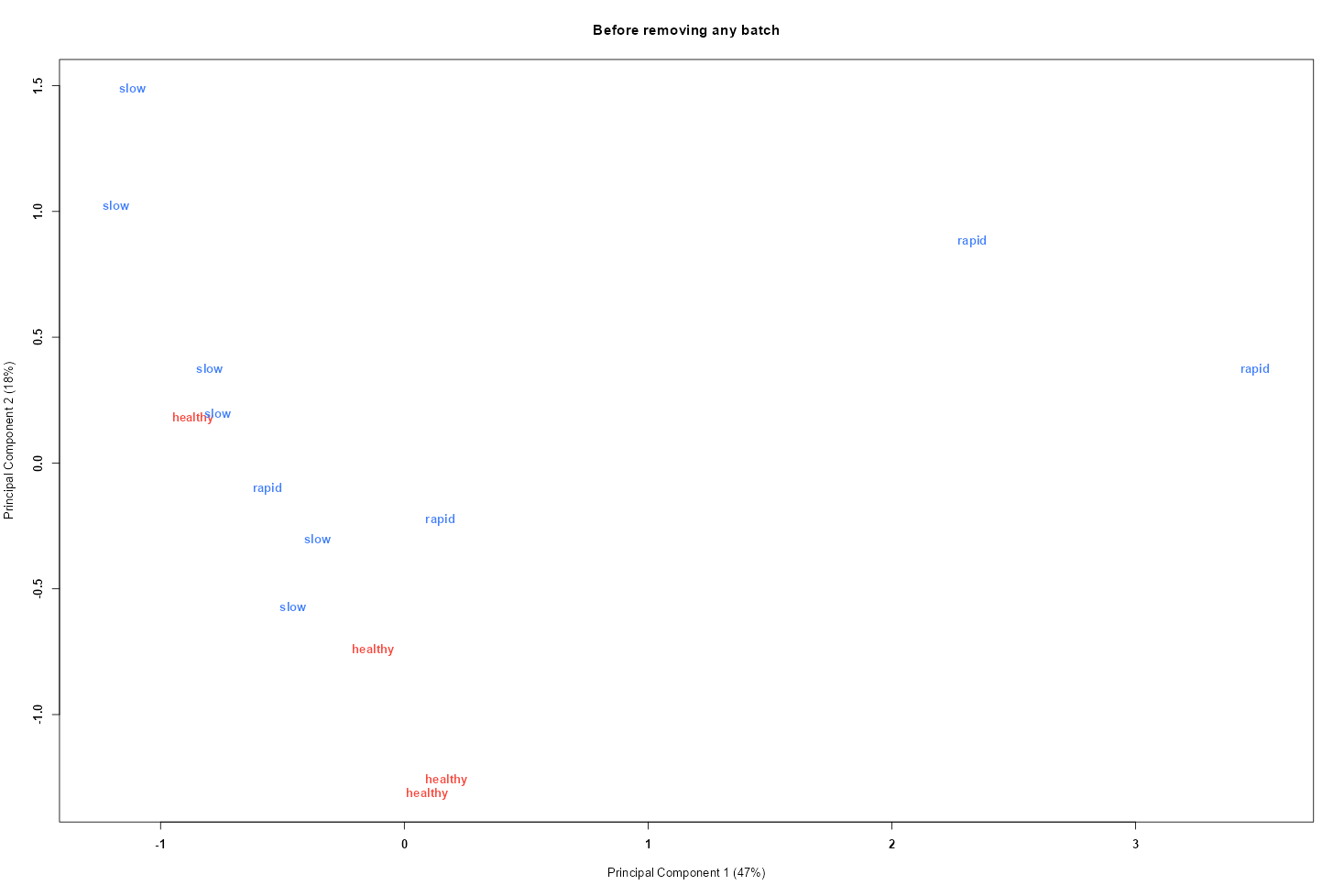

Supplement: Supplementary file 1 [file mmc1.zip › Supplementary_material/DNA-microarray/GSE44723/GSE44723_MDS_before_correction_disease_state.png]

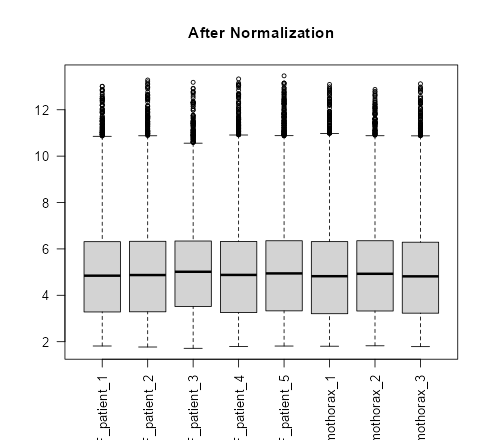

Supplement: Supplementary file 1 [file mmc1.zip › Supplementary_material/DNA-microarray/GSE72073/GSE72073_boxplot_after_normalization.png]

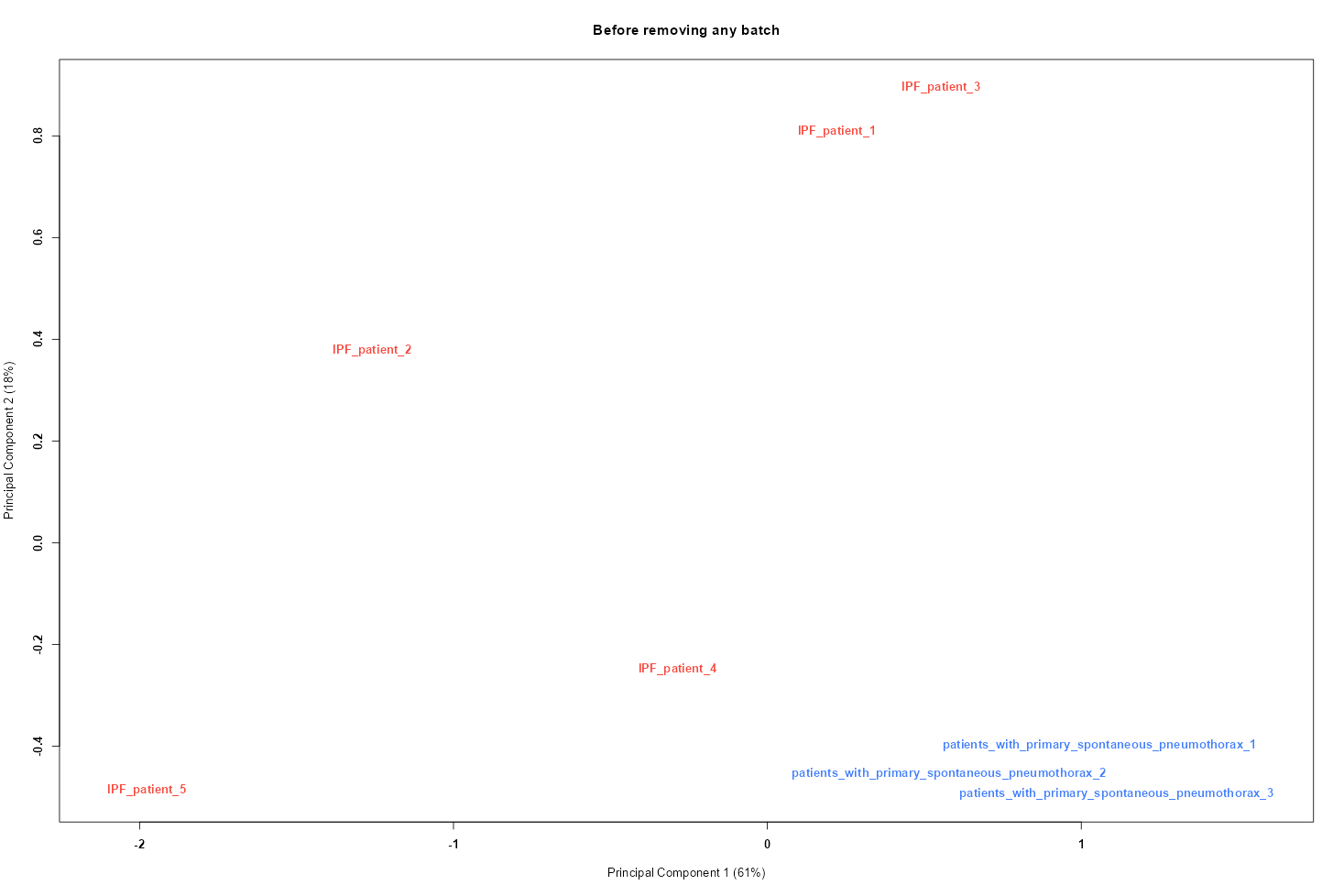

Supplement: Supplementary file 1 [file mmc1.zip › Supplementary_material/DNA-microarray/GSE72073/GSE72073_MDS_before_correction.png]

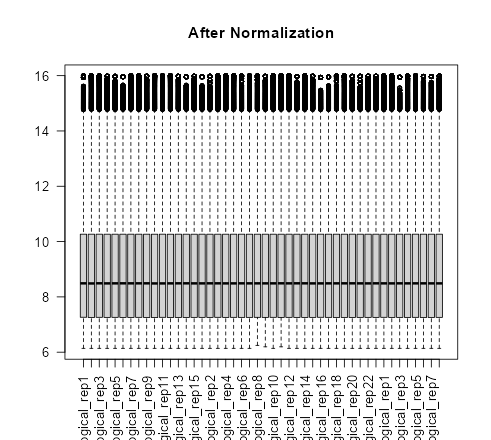

Supplement: Supplementary file 1 [file mmc1.zip › Supplementary_material/DNA-microarray/GSE10667/GSE10667_boxplot_after_normalization.png]

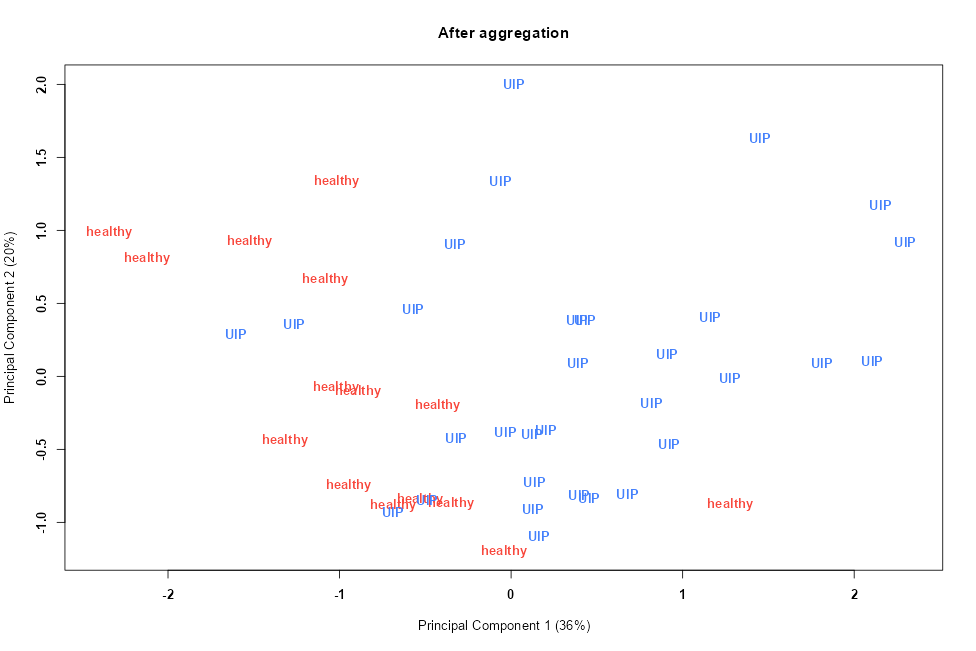

Supplement: Supplementary file 1 [file mmc1.zip › Supplementary_material/DNA-microarray/GSE10667/GSE10667_MDS_after_aggregation_disease.png]

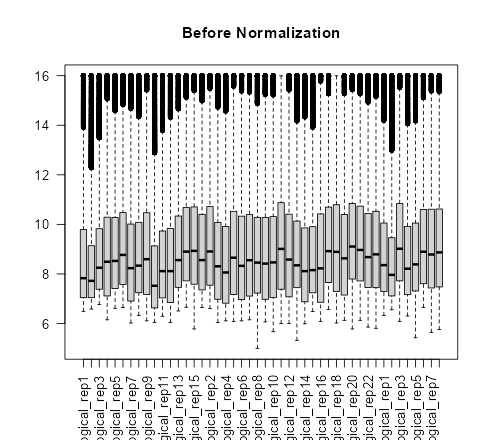

Supplement: Supplementary file 1 [file mmc1.zip › Supplementary_material/DNA-microarray/GSE10667/GSE10667_boxplot_before_normalization.png]

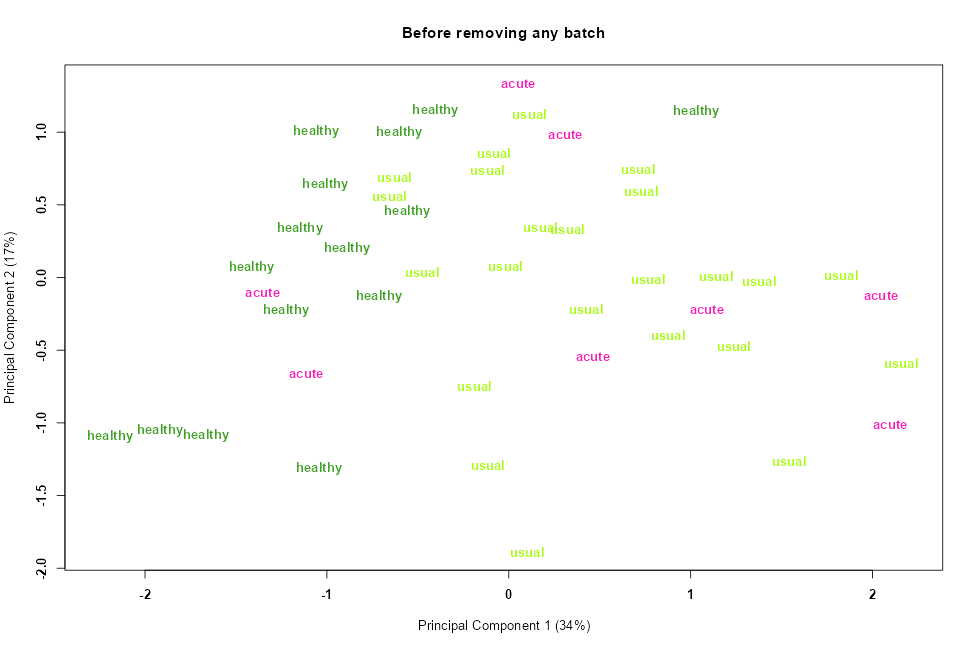

Supplement: Supplementary file 1 [file mmc1.zip › Supplementary_material/DNA-microarray/GSE10667/GSE10667_MDS_before_correction_disease_state.png]

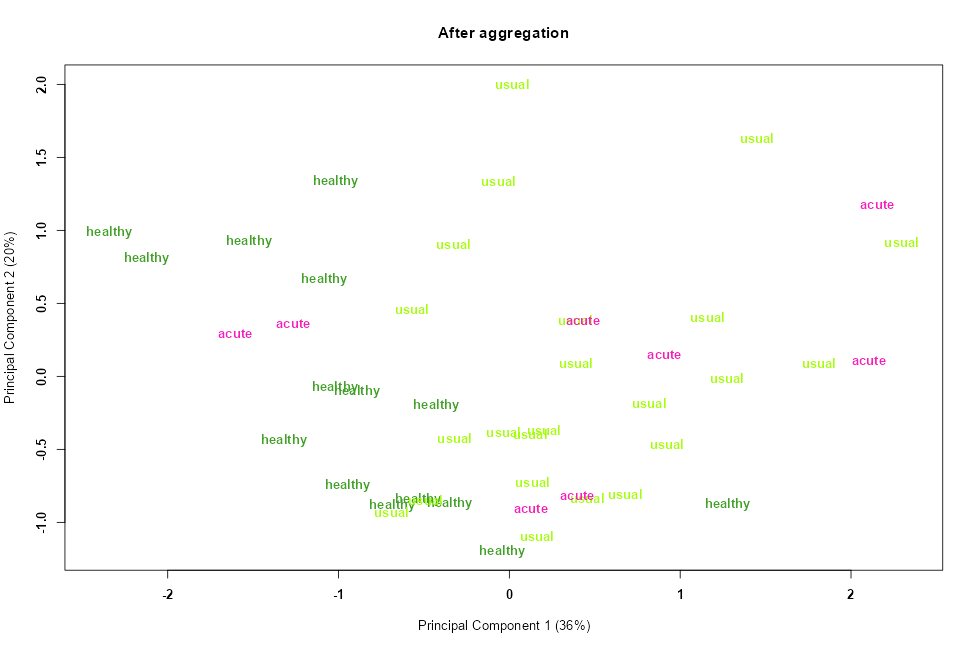

Supplement: Supplementary file 1 [file mmc1.zip › Supplementary_material/DNA-microarray/GSE10667/GSE10667_MDS_after_aggregation_disease_state.png]

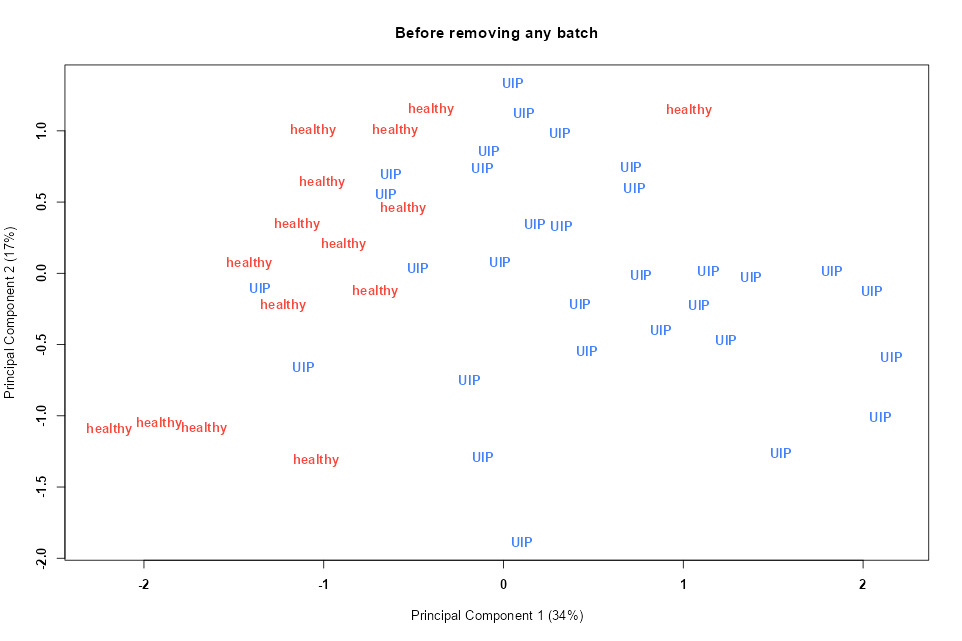

Supplement: Supplementary file 1 [file mmc1.zip › Supplementary_material/DNA-microarray/GSE10667/GSE10667_MDS_before_correction_disease.png]

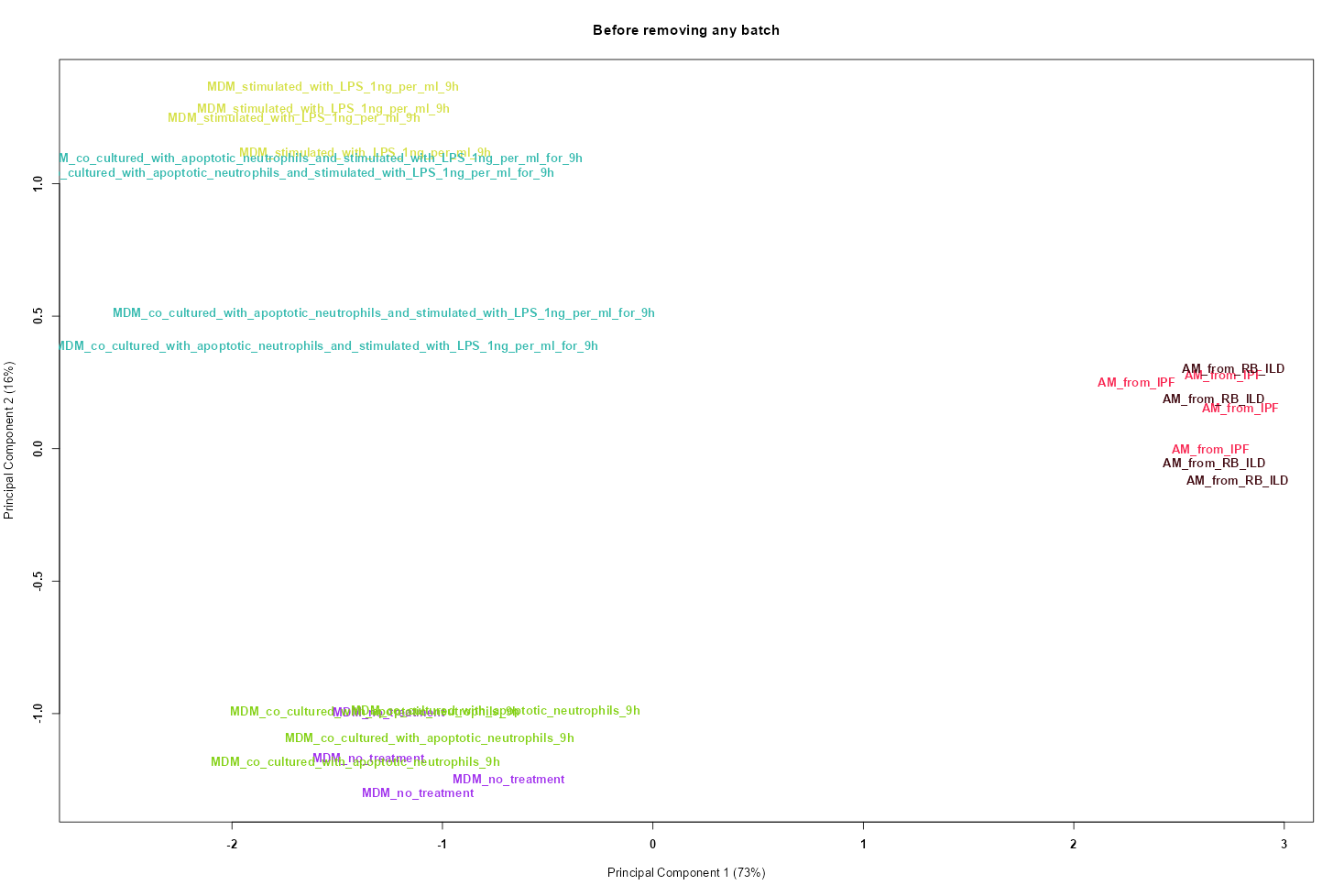

Supplement: Supplementary file 1 [file mmc1.zip › Supplementary_material/DNA-microarray/GSE90010/GSE90010_MDS_before_correction.png]

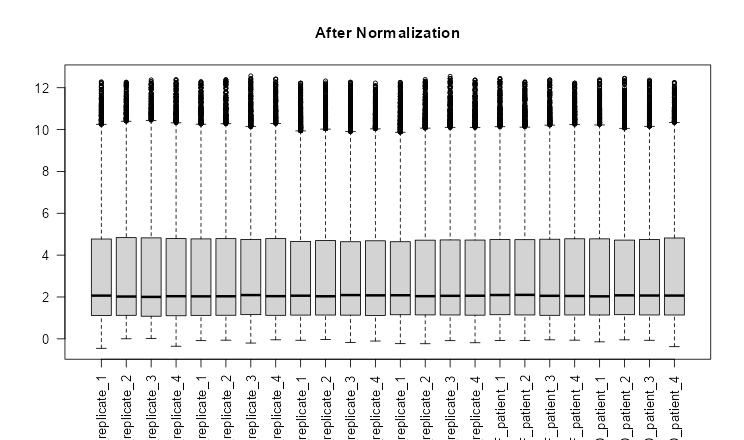

Supplement: Supplementary file 1 [file mmc1.zip › Supplementary_material/DNA-microarray/GSE90010/GSE90010_boxplot_after_normalization.png]

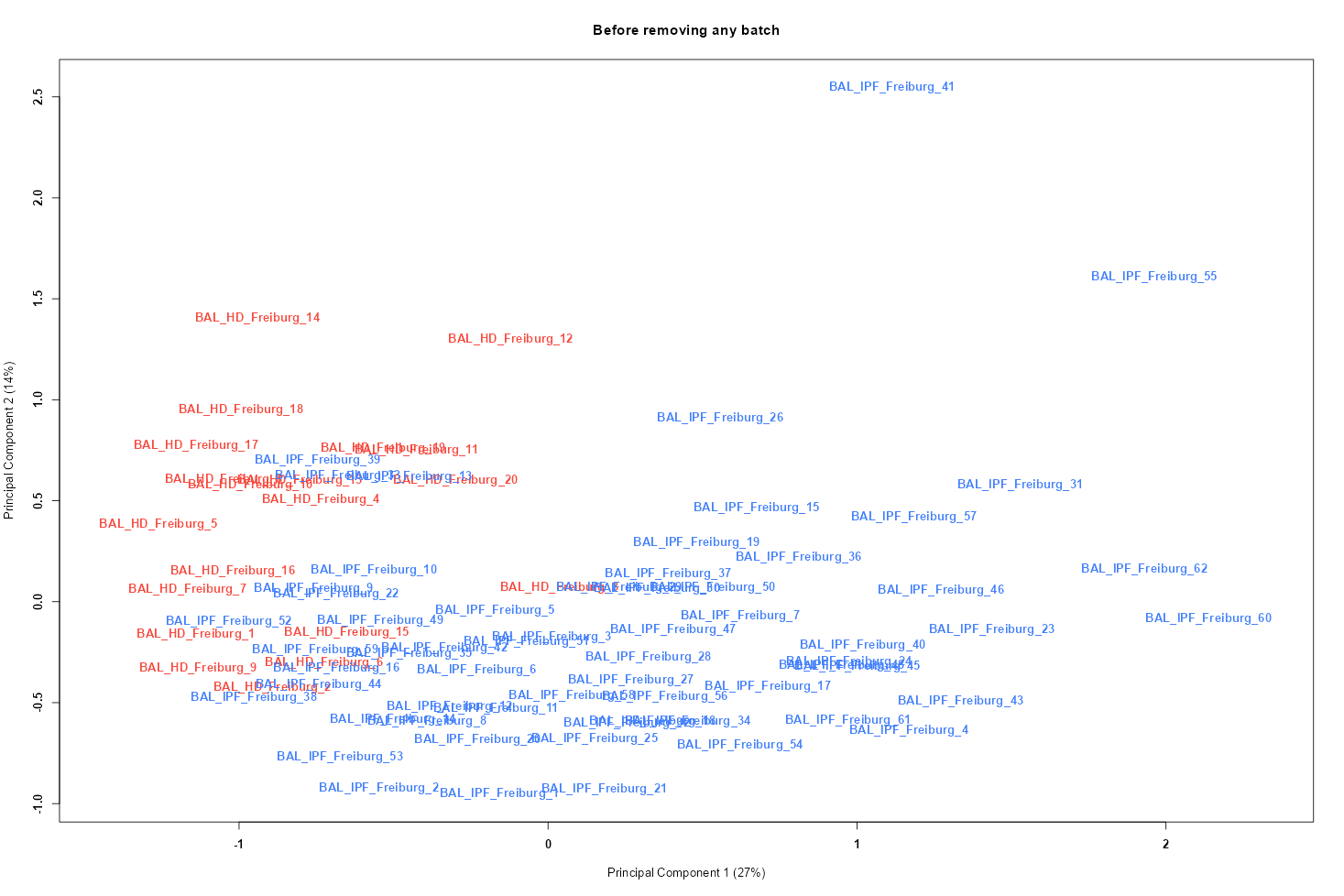

Supplement: Supplementary file 1 [file mmc1.zip › Supplementary_material/DNA-microarray/GSE70866/GSE70866_MDS_before_correction_freiburgh.png]

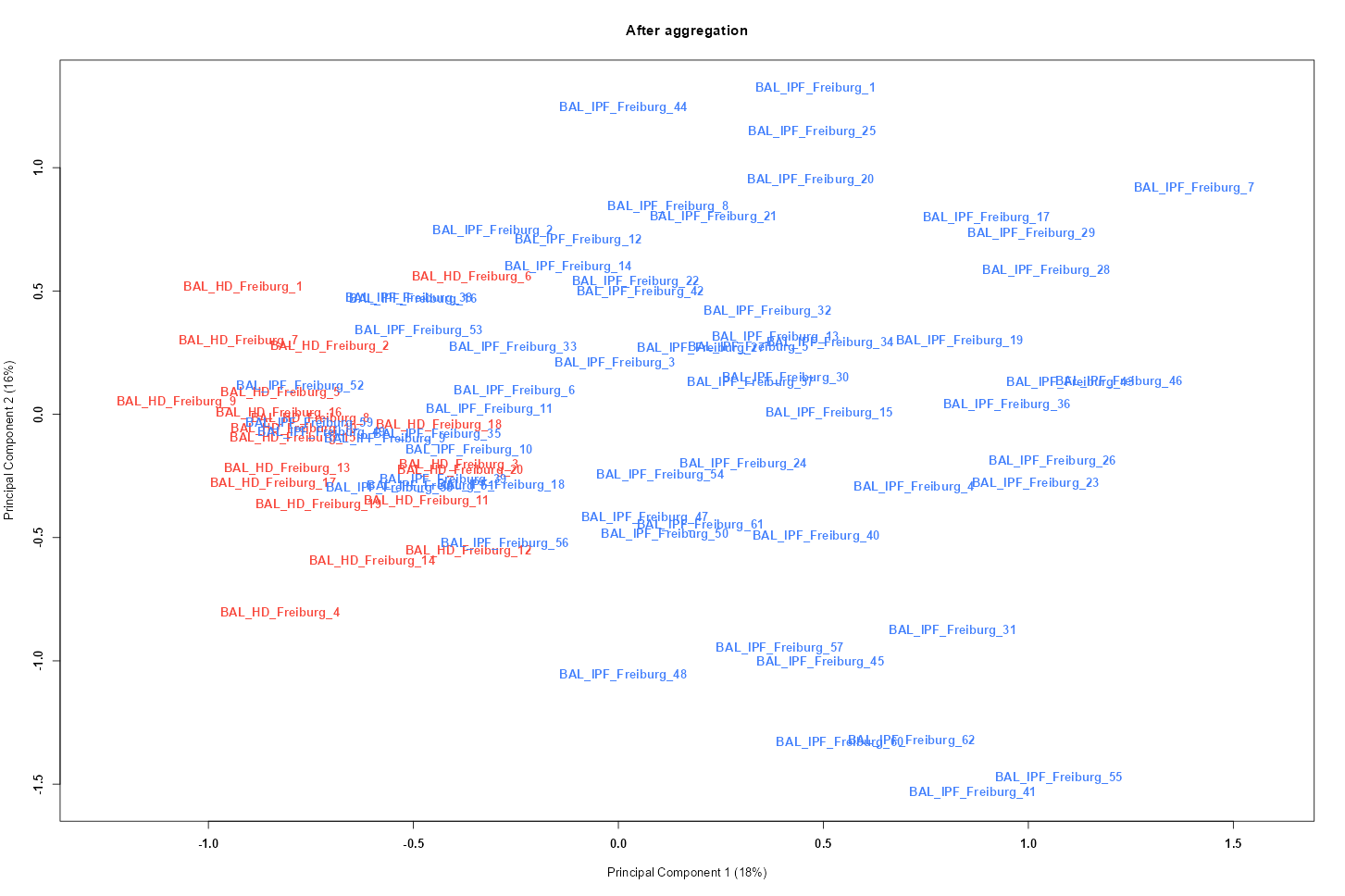

Supplement: Supplementary file 1 [file mmc1.zip › Supplementary_material/DNA-microarray/GSE70866/GSE70866_MDS_after_aggregation_freiburgh.png]

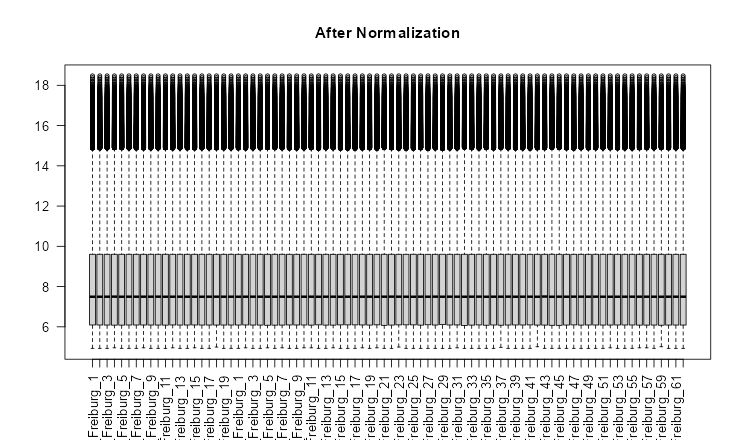

Supplement: Supplementary file 1 [file mmc1.zip › Supplementary_material/DNA-microarray/GSE70866/GSE70866_boxplot_after_normalization_freiburg.png]

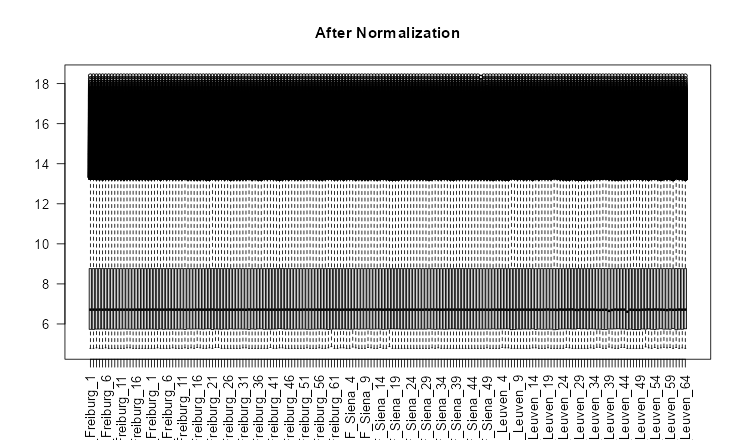

Supplement: Supplementary file 1 [file mmc1.zip › Supplementary_material/DNA-microarray/GSE70866/GSE70866_boxplot_after_normalization_all_samples.png]

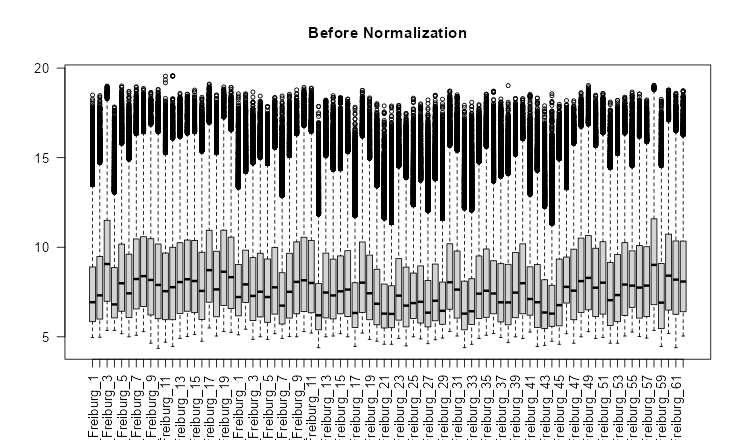

Supplement: Supplementary file 1 [file mmc1.zip › Supplementary_material/DNA-microarray/GSE70866/GSE70866_boxplot_before_normalization_freiburgh.png]

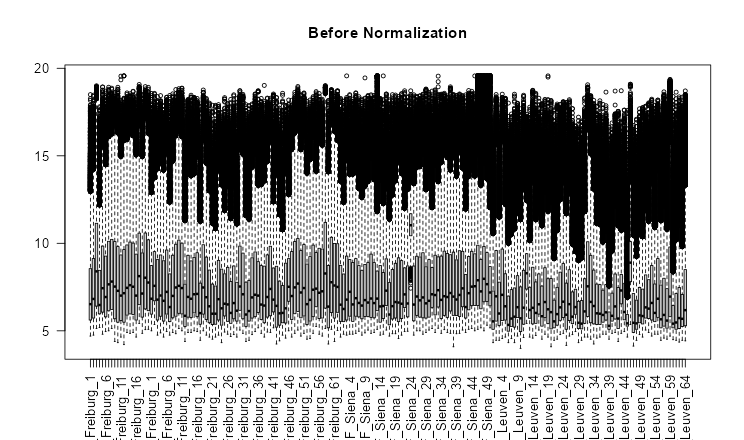

Supplement: Supplementary file 1 [file mmc1.zip › Supplementary_material/DNA-microarray/GSE70866/GSE70866_boxplot_before_normalization_all_samples.png]

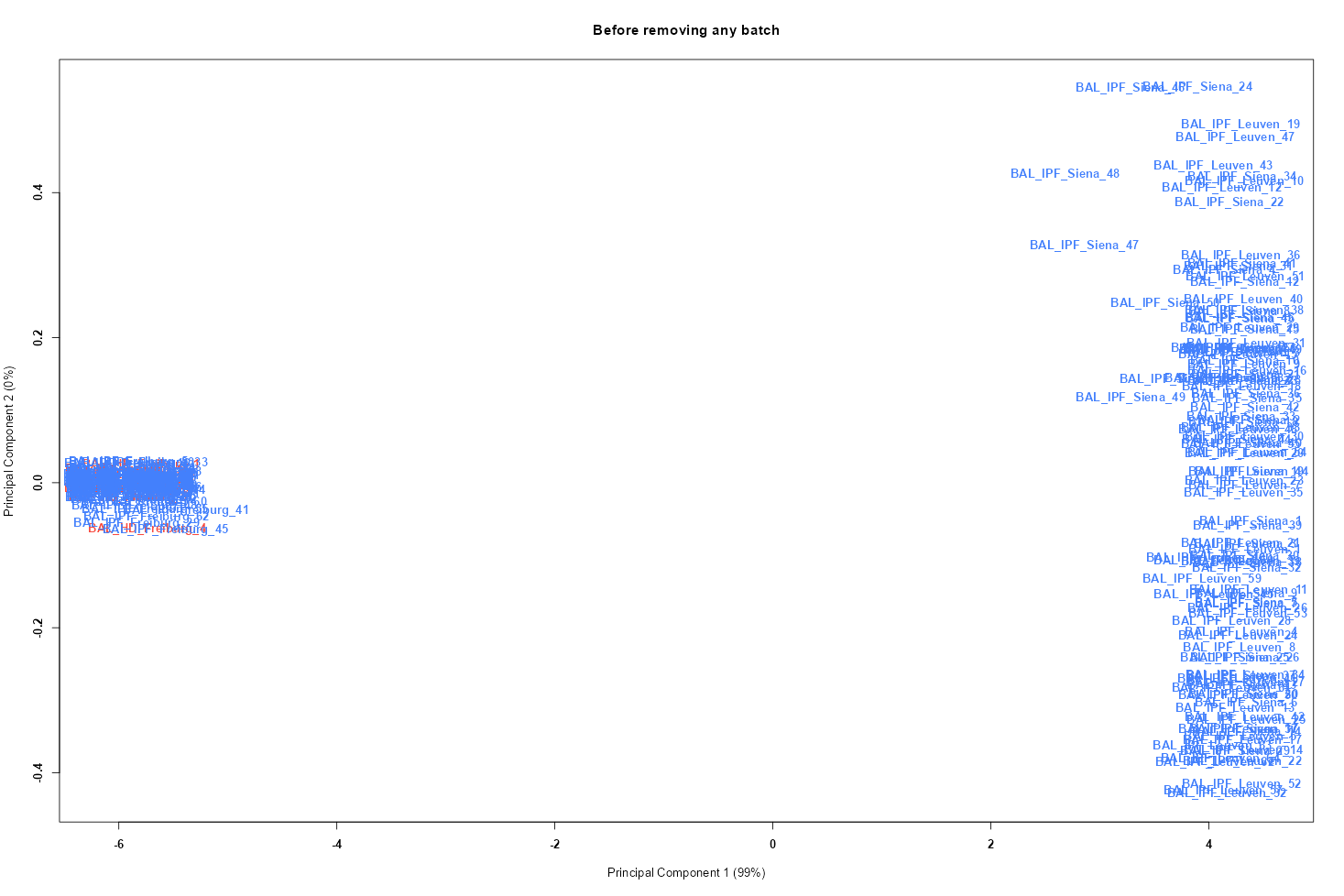

Supplement: Supplementary file 1 [file mmc1.zip › Supplementary_material/DNA-microarray/GSE70866/GSE70866_MDS_before_correction_all_samples.png]
